# Ion-Pair Breakers and Anionic Brønsted Acids for Helmholtz-Layer Catalysis with External Electric Fields in Microfluidic Capacitors

**DOI:** 10.1021/jacsau.5c01705

**Published:** 2026-04-10

**Authors:** Miguel Paraja, Stefan Matile

**Affiliations:** † Department of Organic Chemistry, 27212 University of Geneva, 1211 Geneva, Switzerland; ‡ National Centre of Competence in Research (NCCR) Molecular Systems Engineering, 4002 Basel, Switzerland

**Keywords:** electric-field catalysis, catalytic Helmholtz layers, supramolecular catalysis, Brønsted acid catalysis, anion receptors, flow chemistry, electric double layers

## Abstract

Despite strong support from theory and biological systems, catalysis with externally applied electric fields remains difficult to implement for practical organic synthesis purposes. We approached this central challenge last year with catalytic Helmholtz layers in microfluidic capacitors: in response to applied electric fields (AEFs), specifically designed ion pairs were found to separate and assemble into Helmholtz layers, generating strong, effective electric fields (EEFs) that catalyze reactions. The objective of this study was to identify fundamental principles to construct catalytic Helmholtz layers, focusing on ion-pair separation and the integration of functional motifs. Epoxide-opening polyether cascade cyclizations are used as benchmark reactions because they work in apolar solvents and offer mechanistic diversity and thus a wide responsive product space. With tight ion pairs like tetrabutylammonium bisulfate in apolar solvents, reaction yields Y increase nonlinearly with applied voltage V, starting from 0%. We attributed these nonlinear YV curves to the formation of catalytic Helmholtz layers with high EEFs above a critical voltage required for ion-pair separation. Electric-field catalysis (EFC) then proceeds through stabilization of the transition state by the EEFs, assisted by anionic Brønsted acids that are acidified by the EEFs of their own Helmholtz layers. Inactivity with nonfunctional ion pairs, the absence of detectable currents ≥1 mA up to 20 V, and insensitivity to radical scavengers support this mechanism. To facilitate ion-pair separation and integrate molecular recognition motifs into the catalytic Helmholtz layers, anion-binding ion-pair breakers are introduced. Centered around Schreiner thioureas, these breakers lower the critical voltage, increase maximal yields at saturation to completion, and influence selectivity. With the concepts of voltage-gated EFC, ion-pair breakers, and anionic Brønsted acids in functional Helmholtz layers, the general understanding reached in this study provides the conceptual mechanistic framework for the development of practical electric-field organic synthesis at high voltage.

## Graphical abstract

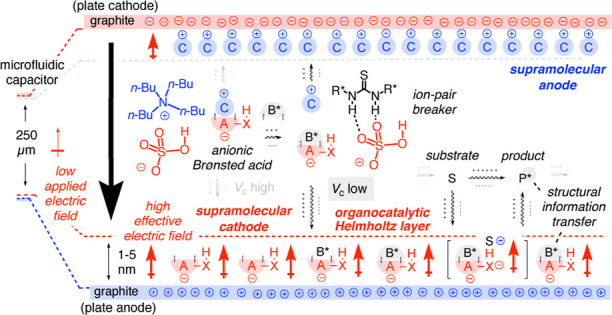

## Introduction

The appeal to enable, accelerate, and direct electron movements during organic reactions with externally applied electric fields is irresistible.^1–7^ Support from theory, intrinsic internal electric fields in enzymes,^6–11^ enzyme mimics,^9,12^ droplets,^3,13–17^ bubbles,^18^ coacervates,^19^ or peeling tapes,^20^ as well as pioneering studies with STM tips,^21,22^ on-surface synthesis,^23,24^ and electrochemical cells,^25–30^ is promising. Perspectives from sustainable industrial production^1,2^ to the understanding of the origin of life are tantalizing.^3,8^ The central problem with catalysis with externally applied electric fields (AEFs), however, is their incompatibility with practical organic synthesis conditions.

Recently, we^31–34^ and others^35^ have explored microfluidic capacitors to address this problem (Figure 1A). During our initial studies, we realized that under practical organic synthesis conditions, AEFs strong enough to directly catalyze reactions will presumably never be accessible.^31^ In its simplest form, electric-field catalysis (EFC) operates by preferential stabilization of transition-state dipoles that are more polarized than those in the ground state.^1–7,31^ According to theoretical predictions, electric fields >1 V/nm are required to catalyze molecular transformations.^1–7^ Defined as voltage divided by the distance between the electrodes, electric fields >0.01 V/nm, i.e., >10 kV/mm, are already beyond reach with microfluidic capacitors under practical organic synthesis conditions. We concluded that we have to shift attention from the AEFs as such to their consequences. In capacitors, these consequences are the assembly of electric double layers. They are composed of a compact, Helmholtz or Stern layer of uniformly charged ions (or oriented polar solvents), followed by a less ordered diffuse layer. The short distance (1–5 nm) of the Helmholtz layer to the plate electrode of opposite charge generates local effective electric fields (EEFs) that can exceed the AEFs by more than one million (Figure 1B).^31^ Indeed, EEFs >1 V/nm have been measured by chronoamperometry for electric double layers formed by oriented polar MeCN solvent molecules,^35^ which is consistent with operational EFC.^1–7^

**Figure 1:**
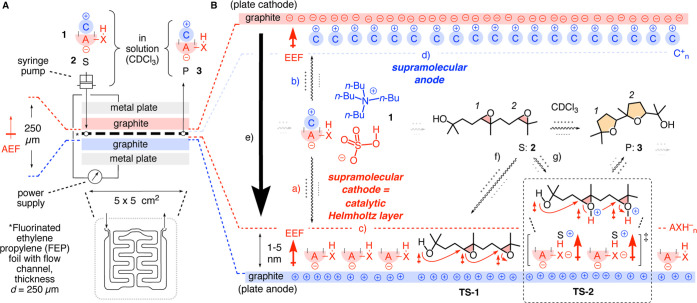
(A) The microfluidic capacitors used in schematic side view above a top view on the central FEP foil with the flow channel that separates the graphite plate electrodes by 250 μm (for photos, see Figures S1–S3). (B) Proposed mechanism of EFC with anionic Brønsted acids: upon introduction of a solution of ion pair **1**, composed of the anionic acid AXH^–^ and counterion C^+^ and substrate S (**2**) in CDCl_3_, into the capacitor under an AEF, applied with a power supply, the ion pair **1** dissociates into (a) anion AXH^–^ and (b) cation C^+^. These ions assemble into Helmholtz layers (c) AXH^–^_n_ and (d) C^+^_n_ to (e) reduce the effective distances between the effective electrodes. The short distance from the supramolecular cathode AXH^–^_n_ to the plate anode (1–5 nm) results in EEFs that are strong enough to (f) directly activate nucleophile and leaving groups as in **TS**-**1** and to (g) increase the acidity of the acids AXH^–^ in their Helmholtz layers AXH^–^_n_ and enable proton transfer as in **TS**-**2**, leading to product P (**3**).

These thoughts implied that the compact Helmholtz layers could be considered as supramolecular electrodes with additional active sites that can be engineered according to the principles of supramolecular chemistry. Last year, we explored this concept with bioinspired Helmholtz layers composed of polyarginine and analogs as supramolecular cathodes and pyrenebutyrate and other hydrophobic anions as supramolecular anodes.^31^ Polyarginine Helmholtz layers in microfluidic capacitors were found to triple the yield of proline-catalyzed aldol condensations and, at best, to increase enantioselectivity depending on the layer architectures.

These findings promised access to catalysis with externally applied electric fields under practical organic synthesis conditions. They also implied that the mechanisms at work differ from those of conventional reactions in stirred solutions. Design strategies for ion-pair separation rather than ion-pair formation, for instance, or the templated assembly of uniformly charged layers with molecular recognition sites will have to be based on the principles of supramolecular chemistry. Understanding these underlying principles will be of critical importance to elaborate on the high expectations for organic synthesis under externally applied electric fields. The objective of this study was to develop this conceptual and methodological framework, focusing on ion-pair separation and the integration of functional motifs into the catalytic Helmholtz layers.

## Results and Discussion

### Systems Design

Commercially available microfluidic capacitors were used as described (Figure 1A, photos: Figures S1–S3). They consist of two 5 × 5 cm^2^ plate electrodes between outer metal plates that are screwed together. The plate electrodes are separated by a *d* = 250 μm thick fluorinated ethylene propylene (FEP) foil with a flow channel. Graphite plate electrodes were used because, compared to metallic electrodes, they show minimal structural changes at high voltage and could possibly contribute to EFC with cation–π and anion–π interactions.^33^ They were systematically and carefully polished before each experiment using abrasive paper and a diamond suspension on a dedicated polishing machine to maximize reproducibility. Voltage was applied by using a power supply, which also reported the resulting current in real time. Solutions of substrates, ion pairs, and additional components were introduced into the microfluidic capacitor with a syringe pump (Figures 1A and S3).

Toward understanding catalysis by externally applied electric fields, the use of apolar solvents such as chloroform was important. They will prevent contributions, or at worst competition, from Helmholtz layers formed by oriented polar solvent molecules.^31^ Most importantly, these solvents enable the application of high voltages in microfluidic capacitors without generating currents that could trigger electron-transfer radical redox chemistry.

The commercially available tetrabutylammonium (TBA) bisulfate **1** was selected as the primary ion pair because we expected the acidity of anionic Brønsted acids to increase in the Helmholtz layers they produce, thereby contributing to EFC (Figure 1B, p*K*_a_ = 2 in water).^36–39^ The ion-pair **1** should separate within the microfluidic capacitor under AEFs to bisulfate anions AXH^–^ and ammonium cations C^+^ (Figure 1Ba,b). The anions should migrate to the plate anode surface, where they assemble into Helmholtz layers AXH^–^_n_ as the most relevant part of the electric double layers for EFC (Figure 1Bc). Because of their short distance to the plate anode, these supramolecular cathodes AXH^–^_n_ should produce EEFs >1 V/nm^35^ that are strong enough to catalyze reactions (Figure 1Bc). At the plate cathode, the ammonium cations C^+^ should form the complementary supramolecular anode generating equally strong EEFs (Figure 1Bd).

Epoxide-opening polyether cyclizations were selected as benchmark reactions of significance in chemistry and biology.^40–48^ These reactions were attractive because they can occur in apolar solvents like chloroform. Diepoxides **2** have served particularly well in the past to elaborate supramolecular catalysis because the wide variety of mechanisms accessible provides a rich product space that reports with high sensitivity to changes in the system of interest, covering chemo-, diastereo-, and potentially, enantioselectivity (Figure 1B).^49^ Product **3** from *exo* cascades is intrinsically favored (see below). Single epoxide-opening monoether cyclizations of the corresponding, pyrene-interfaced monoepoxides^33,34^ and related ring-opening reactions^17,30^ have been used before to explore EFC in several different systems, including carbon nanotubes and droplets. Cyclization cascades from diepoxides **2** were of particular interest in the context of this study because of their revealing product space. Substrates **2** could orient in catalytic Helmholtz layers in a manner that the strong EEF activates both the epoxide opening and the alcohol nucleophile, as outlined in transition state **TS**-**1** (Figure 1Bf). The EEF in the catalytic Helmholtz layer AXH^–^_n_ could further increase the acidity of its Brønsted acid components AXH^–^. Brønsted acid catalysis promised to be particularly responsive to externally applied electric fields.^25^ Proton transfer from acidified anionic acids AXH^–^ to the epoxides in substrate **2** should then activate their opening (Figure 1Bg). The activation of more than one epoxide in a cascade cyclization could be particularly attractive because multiple substrate protonation as in **TS**-**2** is disfavored in solution due to charge repulsion but should be unproblematic in supramolecular cathodes AXH^–^_n_ formed by closely packed anions AXH^–^. Direct activation of the nucleophile and leaving group by the EEFs as in **TS**-**1** should further contribute to cascade cyclizations along **TS**-**2** (Figure 1Bg).

### Methods Development

Solutions of ion pairs like **1**, substrates like **2**, and additional components in CDCl_3_ were introduced into the microfluidic capacitor at constant flow rate at room temperature (Figures 1A and S3). Consistent with an operational capacitor, *I* = 0.000 A was recorded in all experiments at the steady state. Without significant currents, temperatures stayed constant.

Reactions were characterized with their microfluidic yields, *Y*_m_ (Figure 2), which is the yield determined by ^1^H NMR spectroscopy after one passage through the microfluidic capacitor. *Y*_m_ can be considered as a single data point in the course of the reaction and can be varied by varying the flow rate. Despite the wide product space, the reaction products can be easily identified by comparing the ^1^H NMR spectrum of the crude reaction mixture to those of all possible products that were previously identified.^49^ The integrals of baseline-separated signature peak clusters from substrate and products were measured and compared to unreactive internal standards (mesitylene, Figure S5; see below). Consistent with the cleanliness of the reaction, the yields determined from the increasing product peak and the decreasing substrate peak were nearly identical, so the latter was used as *Y*_m_ for convenience. Microfluidic yields *Y*_m_ without applied voltage were described as *Y*_m_^0^, microfluidic yields *Y*_m_ at saturation at high voltage were described as *Y*_m_^max^, and the dependence of *Y*_m_ on applied voltage *V* is described as YV curves.

**Figure 2:**
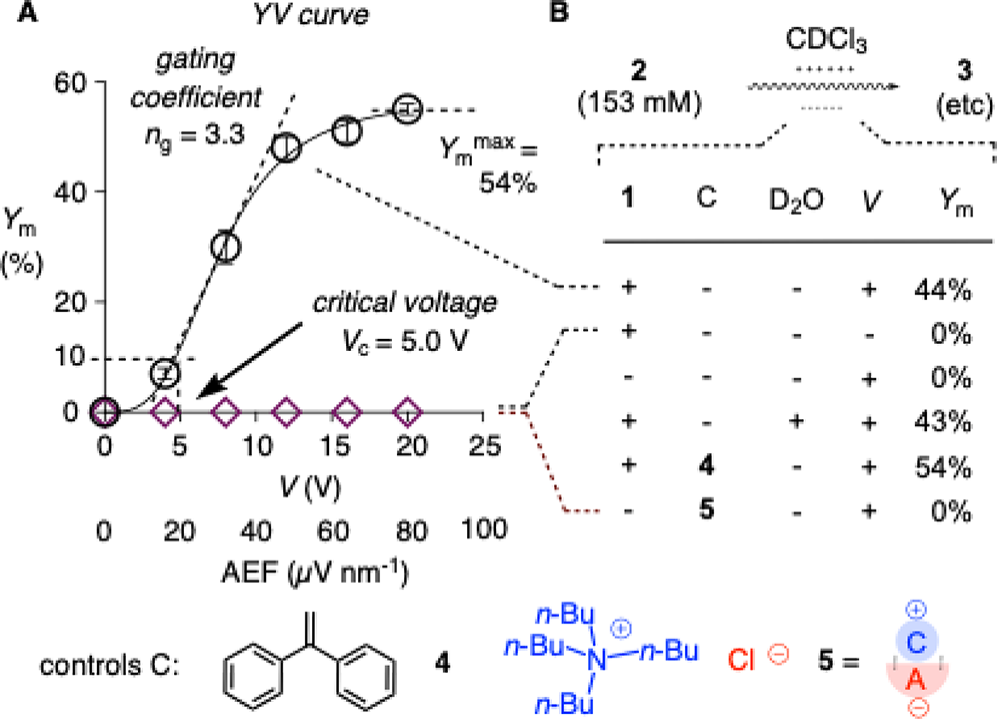
(A) Dependence of the yield *Y* after one passage through the microfluidic capacitor (microfluidic yield *Y*_m_) on the applied voltage *V* (YV curve) for substrate **2** (153 mM) with acid **1** (circles) or **5** (diamonds, 0.25 mol %, 38 μM) in CDCl_3_ at room temperature. (B) Control experiments at *V* = 12 V (+) or *V* = 0 V (−) covering controls C (radical scavenger **4**, nonacidic TBACl ion pair **5** at 0 (−) or 0.25 mol % (+)) and D_2_O content in commercial (−) and D_2_O-saturated CDCl_3_ (+); steady-state *I* = 0.000 A in all experiments.

With 153 mM substrate **2** and a low 0.25 mol % (38 μM) of anionic acid **1** in CDCl_3_ at room temperature, no conversion was observed without applied voltage (*Y*_m_^0^ = 0%, Figure 2). With increasing voltage, the conversion of substrate **2** increased substantially to *Y*_m_^max^ = 54% at 20 V. The YV curve showed sigmoidal behavior. Adapting the terminology of voltage-gated ion channels,^50^ Hill analysis gave a gating (Hill) coefficient *n*_g_ = 3.3 and a critical voltage *V*_c_ = 5.0 V as the voltage that gives *Y*_m_ = 10% (Figures 2 and S4–S6 and Table S1). A plausible interpretation of these YV characteristics was that

(i) the critical voltage *V*_c_ describes the voltage or AEF needed to separate the ion pairs (the lower the easier, Figure 1Ba,b);
(ii) the gating coefficient *n*_g_ describes the AEF needed to assemble the best-performing catalytic Helmholtz layer (the higher the easier, Figure 1Bc,e);
(iii) the maximal microfluidic yield *Y*_m_^max^ describes the catalytic activity of this best-performing catalytic Helmholtz layer (the higher the better, Figure 1Bf,g).

Control experiments confirmed that the presence of trace D_2_O had little influence on the reaction (Figures 2B and S42). This was reasonable because D_3_O^+^ (p*K*_a_ = 0) is more acidic than bisulfate (p*K*_a_ = 2), misses the negative charge to integrate into catalytic Helmholtz layers at the plate anode, and would be rather inactivated than activated if formed in Helmholtz layers at the plate cathode (Figure 1B).

Consistent with *I* = 0.000 A recorded in all experiments at steady state after formation of the electric double layers and the low ion-pair concentrations (38 μM), the presence of radical scavenger **4** ^51^ did not inhibit the reaction (Figure 2B). With TBA chloride **5** in place of TBA bisulfate **1**, the cascade cyclization of **2** did not occur, independent of the applied voltage (Figure 2A, diamonds, B, S40–S41). These results provided corroborative support that the redox chemistry from parasitic faradaic processes is not significant under the developed conditions. More importantly, the inactivity of TBA chloride **5** suggested that Helmholtz layers formed by chloride and their EEFs only are insufficient to catalyze the cyclization of diepoxide **2** along **TS**-**1** (Figure 1Bf). This finding could support that the acidification of bisulfate AXH^–^ by the EEFs from their own Helmholtz layers AXH^–^_*n*_ contributes to EFC in the presence of TBA bisulfate **1**, as outlined in **TS**-**2** (Figure 1Bg).

### Ion-Pair Breakers

Definition and interpretation of YV characteristics implied that the emergence of voltage-gated EFC originates from the critical voltage *V*_c_ needed to separate the tight ion pairs in apolar solvents (Figures 2A and 1Ba,b). If so, then the critical voltage could be lowered by facilitating ion-pair separation. Neutral anion-binding molecules like activated thioureas, frequently used in synthetic methodology,^52–54^ and phenols were considered to serve as ion-pair breakers B* (Figure 3).^52–58^ Binding of the breaker to anionic acid AXH^–^ in **1** should loosen the ion pair and thus lower the critical AEF needed to separate anions from cations (Figure 3a) and drive them to the plate anode (Figure 3b). Integration of B* into the supramolecular cathodes B*AXH^–^ (Figure 3c) could further aide the acidification of the anionic acids AXH^–^ by the EEF in this catalytic Helmholtz layer and add molecular recognition motifs for structural information transfer to the product P* (Figure 3d).

**Figure 3:**
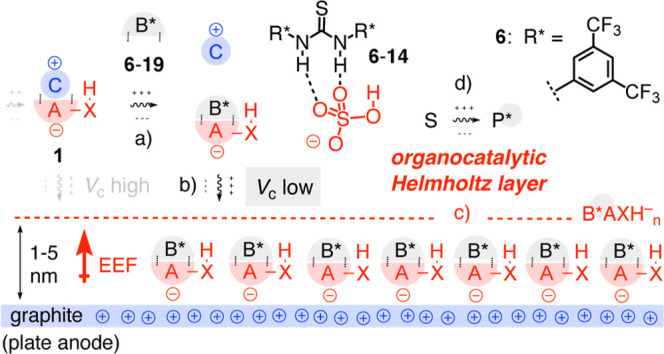
Concept of ion-pair breakers: (a) neutral breakers B* bind to anions AXH^–^ to (b) facilitate their separation from cations C^+^ by AEFs in microfluidic capacitors. (c) Integrated into Helmholtz layers B*AXH^–^, breakers B* are expected to contribute recognition motifs for (d) the transfer of structural information (*, gray circles) to product P* (compare Figure 1 for the general EFC mechanism).

The presence of 0.25 mol % of Schreiner thiourea catalyst^59^ **6** lowered the critical voltage for the cyclization of **2** with 0.25 mol % bisulfate **1** from *V*_c_ = 5.0 V to *V*_c_ = 2.3 V (Figures 4, squares, S7, and S8, Table S2). The coincident slight decrease in the gating coefficient to *n*_g_ = 2.6 indicated that breakers facilitate the charge separation at lower fields but complicate the assembly of anionic acids into catalytic 2D Helmholtz architectures. The jump of *Y*_m_^max^ from 54% to 92% demonstrated that this integration of thiourea **6** nearly doubled the catalytic activity of the 2D architecture of the final, best-performing Helmholtz layer (Figure 4, empty circles vs squares). Control experiments demonstrated that thiourea **6** without bisulfate **1** was unable to catalyze the reaction even at 20 V (Figures 4, diamonds, S38, and S39, Table S16).

**Figure 4:**
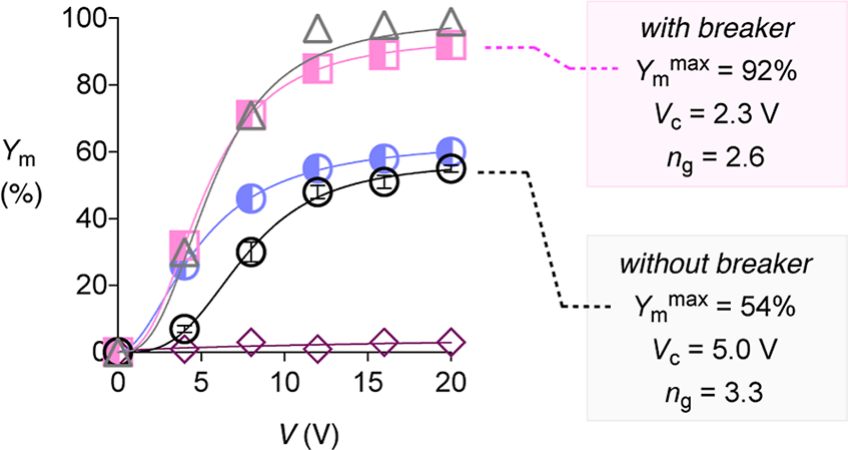
Dependence of the microfluidic yields (*Y*_m_) on the applied voltage *V* (YV curve) for substrate **2** (153 mM) with acid **1** (0.25 mol %) in CDCl_3_ at room temperature in the absence (empty circles, error bars represent SEM of duplicate) and the presence of ion-pair breakers (0.25 mol %) **15** (half-full circles), **6** (squares; control without **1**, diamonds), and **11** (triangles).

Weaker thioureas **7** and **8** gave intermediate YV curves between breaker **6** and breaker-free EFC, characterized by *Y*_m_^max^ = 69% at preserved low *V*_c_ and *n*_g_ (Figures 5, S9–S12 and Tables S3 and S4). Even weaker thioureas **9** and **10** were essentially inactive and possibly not binding with enough strength to the anionic acid **1** (Figures 5, S13–S16 and Tables S5 and S6). Dithioureas **11**–**13** provided reliable access to quantitative yields *Y*_m_^max^ ≥ 97% (Figures 4, triangles, 5, S17–S22 and Tables S7–S9). With extra phenolic hydrogen-bond donors, thiourea **14** exhibited a unique profile that combines powerful ion-pair breaking (*V*_c_ = 2.6 V) and the highest performance of catalytic Helmholtz layers among monothioureas (*Y*_m_^max^ = 97%, Figures 5, S23, and S24, and Table S10).

**Figure 5:**
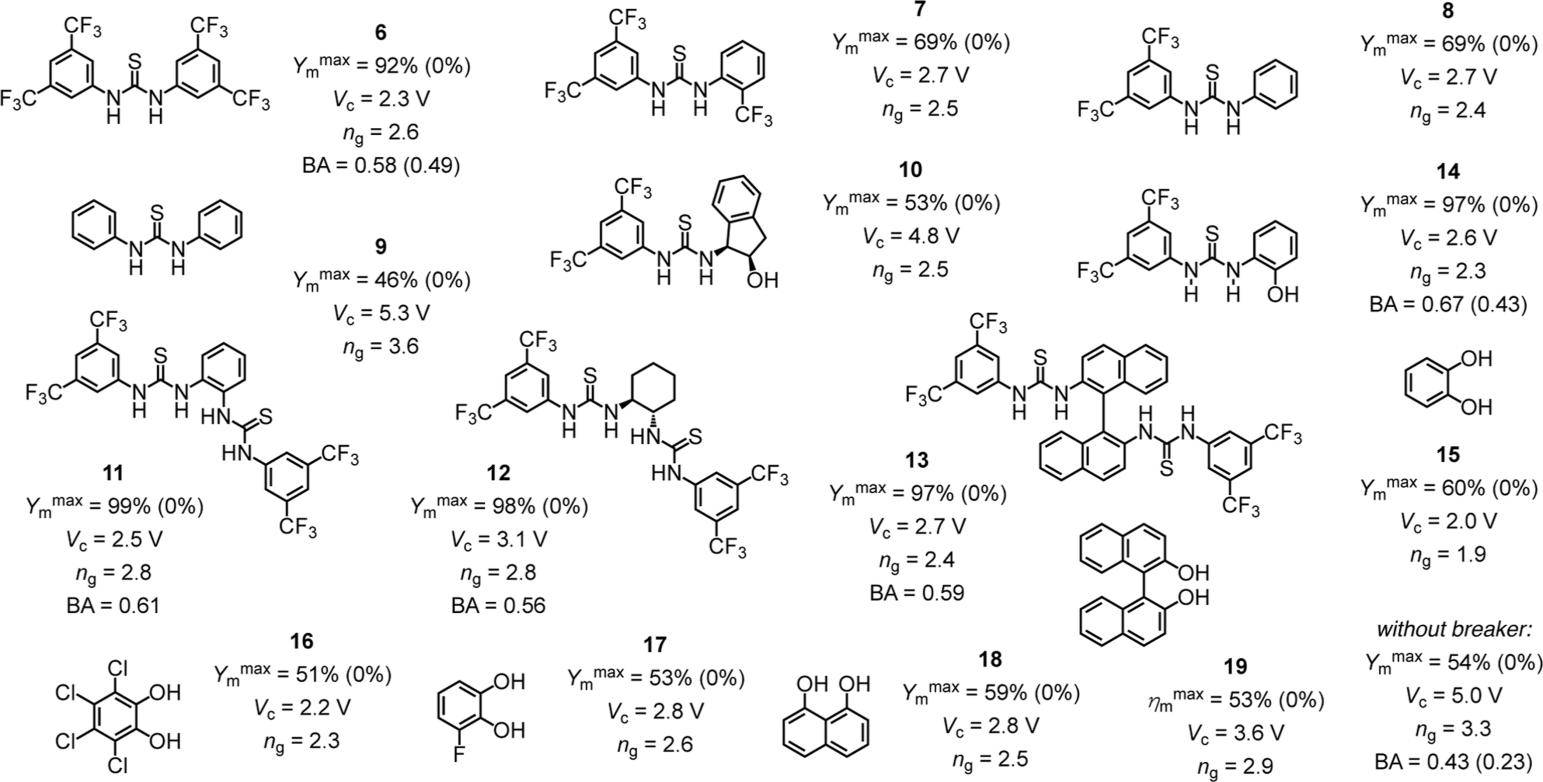
Maximal microfluidic yield *Y*_m_^max^ (*Y*_m_^0^ in parentheses), critical voltage *V*_c_, and gating coefficient *n*_g_ extracted from the YV curves of breakers **6**–**19** screened with substrate **2** and acid **1** (Figures 2 and 4), and BA selectivity at 20 V (BA^0^ without applied voltage at higher catalyst loading in parentheses, Figure 6d).

Minimalist catechols **15** were with a record *V*_c_ = 2.0 V even slightly better ion-pair breakers, but their integration into the catalytic compact layers did not increase performance significantly (*Y*_m_^max^ = 60%, Figures 4, half-filled circles, 5, S25, and S26, Table S11). Withdrawing substituents in **16** and **17** weakened not only the catalytic Helmholtz layers (*Y*_m_^max^ ≤ 53%) but also ion-pair separation (*V*_c_ ≥ 2.0 V), possibly due to competing intramolecular hydrogen bonding (Figure 5, S27–S30, Tables S12 and S13). Expansion of the catechol motif in naphthalene-1,8-diol **18** gave overall disappointing activities (*Y*_m_^max^ = 59%, *V*_c_ = 2.8 V), and BINOL **19** was a weak ion-pair breaker (*V*_c_ = 3.6 V) without contributions to catalysis (*Y*_m_^max^ = 53%, Figures 5, S31–S34, Tables S14 and S15).

Overall, anion binding strength of the breaker appeared to determine the catalytic performance *Y*_m_^max^ of the final Helmholtz layers, with activated dithioureas **11**–**13** all achieving full conversion (Figures 4 and 5). All activated thioureas were efficient ion-pair breakers, lowering the critical voltage from *V*_c_ = 5.0 V to *V*_c_ = 2.3–2.8 V. Catechols were also efficient ion-pair breakers (*V*_c_ = 2.0–2.8 V) but contributed so far little to the activity of the final Helmholtz layers (*Y*_m_^max^ = 51–60%). In catechol-thiourea hybrid **14**, these trends were additive to afford the only thiourea monomer capable of full conversion, slightly exceeding Schreiner thiourea **6**, also with regard to chemoselectivity (see below).

### Other Substrates and Chemoselectivity

The combination of acid **1** and breaker **6** was also able to catalyze the extended cascade cyclization from triepoxide^60,61^ **20** into mostly product **21** to completion in one passage through the microfluidic capacitor at 5 V (*Y*_m_ = 93%, Figures 6a and S57, Table S20). Without an applied electric field, the same reaction did not occur (*Y*_m_^0^ = 0%). The same enabling “on–off” EFC was obtained for cascade cyclization of tetraepoxide^60,61^ **22** into mostly product **23** (*Y*_m_ = 92%, *Y*_m_^0^ = 0%, Figures 6b and S58). Removal of breaker **6** reduced the yields by more than half to *Y*_m_ ≤ 40% (Figures 6a,b, S55, and S56).

**Figure 6:**
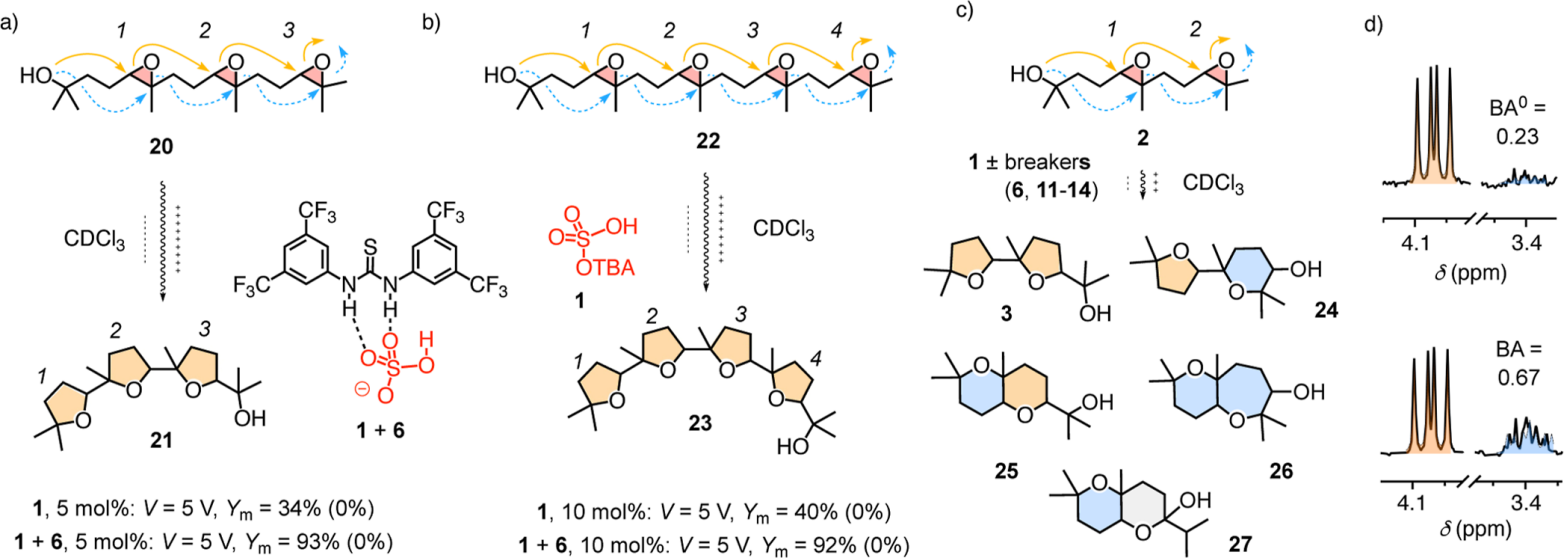
Microfluidic yield *Y*_m_ of the cascade cyclization of (a) triepoxide **20** (with 5 mol % **1**) and (b) tetraepoxide **22** (with 10 mol % **1**, mixtures of *E* and *Z* isomers) at 5 V with and without breaker **6** (*Y*_m_^0^ in parentheses). (c) Possible cyclization products of diepoxide **2** highlighting violations of the Baldwin (B) rules (anti-Baldwin (A) components, blue) in **24**–**27**. (d) ^1^H NMR signatures for B (4.1 ppm) and A cyclizations (3.4 ppm) in representative product mixtures without (top) and with breaker **14** (bottom) at 0 V (top) and 20 V (bottom; for full BA data for **6** and **11**–**14**, see Figure 5).

Cascade cyclizations of diepoxide **2** that follow the Eschenmoser–Dunitz–Baldwin (EDB) rules^62,63^ to product **3** are intrinsically favored (Figure 6c, solid arrows, orange).^40–49^ This Baldwin (B) or *exo* selectivity is as in polyketide natural products of the monensin A family. *Endo* cyclizations that violate the EDB rules would lead to fused polyether products, as in brevetoxin B, for instance. For diepoxide **2**, cascades that include at least one *endo* cyclization lead to the anti-Baldwin (A) products **24**–**27** as a mixture of stereoisomers (Figure 6c, dashed arrows, blue).^49^

A comprehensive study of the dependence of the cyclization selectivity on the EFC was beyond the scope of this study. We have shown that the complexity of the product space requires the use of pure stereoisomers as substrates to accurately interpret results.^49^ Nevertheless, chemoselectivity can be deduced by comparing the ^1^H NMR spectrum of the crude mixture with previously recorded spectra of all possible products.^49^ Even with *E*/*Z* mixtures of diepoxide **2**, signature signals from all-Baldwin product **3** were clearly visible and resolved from the rest just below 4.1 ppm (Figure 6d,c, orange, S45). Violations of the EDB rules could be detected at 3.4 ppm with a resolved signal cluster that contains only contributions from products with at least one expanded ring generated by anti-Baldwin (A) cyclization (Figure 6d,c, blue, S45).^40–48^ To quantify the ability of EFC and particularly ion-pair breakers in catalytic Helmholtz layers to violate the EDB rules and catalyze the formation of intrinsically disfavored products, the integral of the anti-Baldwin cluster divided by the integral of the Baldwin cluster was defined as the BA ratio with and BA^0^ ratio without applied voltage (Figure 5).

Without electric fields, access to the cyclization of diepoxide **2** in CDCl_3_ required catalyst concentrations higher than the 0.25 mol % used to elaborate on EFC (Figures 2 and 4). With 10 mol % of acid **1**, the ^1^H NMR signature gave a BA^0^ = 0.23, that is high selectivity for the intrinsically favored EDB product **3** (Figures 6d, top, 5, and S47, Table S19). This was as expected; violations of the EDB rules have been rare and minor with Brønsted acid catalysis, while pnictogen-bonding catalysis led to a complete breakdown of the EDB rules.^49,60,64^ Application of 20 V at a lower catalyst loading increased the violations of the EDB rule to BA = 0.43 (Figures 5 and S46). In the additional presence of ion-pair breaker **6**, this ratio was already surpassed without an EFC (BA^0^ = 0.49) and increased to an AB = 0.58 at 20 V (Figures 5, S48, and S49). The highest BA = 0.67 was obtained with catechol-thiourea hybrid breaker **14**, which was already among the best for ion-pair breaking (*V*_c_ = 2.6 V) and performance of the catalytic Helmholtz layers (*Y*_m_^max^ = 97%, Figures 6d, bottom, 5, S50, and S51). Equally performant in catalytic Helmholtz layers (*Y*_m_^max^ > 97%), dithioureas **11**–**13** closely followed **14** in violating the EDB rules (BA = 0.56–0.61, Figures 5, S52–S54). These diverse influences of ion-pair breakers on chemoselectivity supported the occurrence of operational information transfer from B* to P* in catalytic Helmholtz layers (Figure 3) and the insignificant contributions of water traces to catalysis (Figure 2B).

## Conclusions

While external electric fields are unlikely to ever become directly applicable,^31^ this study provides the conceptual and methodological understanding to use their consequences to catalyze reactions under organic synthesis conditions. Anionic Brønsted acids are introduced to assemble into catalytic Helmholtz layers that serve as supramolecular cathodes on plate anodes in microfluidic capacitors. Their Helmholtz layers produce the effective electric fields needed to increase their acidity and to enable, accelerate, and direct reactions. A nonlinear dependence of the reaction yield on the applied voltage reveals the existence of a critical voltage needed to separate the anionic acids from their counterions in apolar solvents. To facilitate this ion-pair separation under the applied electric fields, anion-binding ion-pair breakers are introduced. Their integration into the catalytic Helmholtz layers formed by the separated ions provides molecular recognition motifs that contribute to catalysis and selectivity by the transfer of structural information to the product. This emerging understanding of EFC in microfluidic capacitors operates with mechanisms that differ from conventional methodology and is based on principles of supramolecular chemistry. It provides the conceptual framework for an organic synthesis at high voltage that is waiting to be discovered.

## Supplementary Material



## Data Availability

The data that support the findings of this study are openly available in Zenodo at 10.5281/zenodo.19152190.

## Notes

Detailed experimental procedures, materials and methods, procedures for the synthesis of substrates and breakers, configuration of microfluidic capacitors, procedures to record and analyze voltage dependence for reactions, references, and original NMR spectra (PDF)
