# Supplementary material for: Ion-Pair Breakers and Anionic Brønsted Acids for Helmholtz-Layer Catalysis with External Electric Fields in Microfluidic Capacitors
Source: JACS Au. 2026 Apr 10;6(4):2337–44. doi: 10.1021/jacsau.5c01705 (PMC13126189; doi:10.1021/jacsau.5c01705)
Supplement: Supplementary file 1 [file au5c01705_si_001.pdf]

Supporting Information

Ion-Pair Breaker and Anionic Brønsted Acids for Helmholtz-Layer Catalysis with External Electric Fields in Microfluidic Capacitors

Miguel Paraja* and Stefan Matile*

Department of Organic Chemistry, University of Geneva, 1211 Geneva, Switzerland

National Centre of Competence in Research (NCCR) Molecular Systems Engineering, 4002 Basel, Switzerland

*E-mail: miguel.parajaramos@unige.ch, stefan.matile@unige.ch

Table of Contents

1. Materials and Methods S3

2. Synthesis S4

 2.1. Synthesis of Diepoxides, Triepoxides and Tetraepoxides S4

 2.2. Synthesis of Ion-Pair Breakers S5

3. Electric-Field Catalysis S9

 3.1. Microfluidic Capacitors S9

 3.2. General Procedure for Diepoxide Cyclization S13

 3.3. Voltage Dependence of Diepoxide Cyclizations without Ion-Pair Breakers S15

 3.4. Voltage Dependence of Diepoxide Cyclizations with Ion-Pair Breakers S17

 3.5. Control Experiments S46

  3.5.1. Ion-Pair Breakers without Anionic Brønsted Acids S46

  3.5.2. Non-Acidic Anions in Place of Anionic Brønsted Acids S48

  3.5.3. Water Sensitivity S50

  3.5.4. Radical Scavengers S52

 3.6. Voltage Dependence of Regioselectivity of Diepoxide Cyclizations S54

 3.7. Voltage Dependence of Triepoxide and Tetraepoxide Cyclizations S65

4. Supporting References S70

5. NMR Spectra S71

1. Materials and Methods

As described in S1. Reagents for synthesis were purchased from Fluka, Sigma-Aldrich, Alfa Aesar, TCI and Across. Salts of the best grade available from Fluka or Sigma-Aldrich were used as received. Column chromatography was carried out on silica gel 60 (SiliaFlash P60, 40-63 μm). Analytical (TLC) was performed on silica gel 60 (Merck, 0.2 mm). Melting points (Mp) were recorded using a Melting Point M-565 (BUCHI). IR spectra were obtained using a Perkin Elmer Spectrum Two™ FT-IR spectrometer (ATR, Golden Gate), reported as wavenumbers (*ν*) in cm^{-1} with intensities described as broad (br), strong (s), medium (m), or weak (w). All ^{1}H, ^{13}C and ^{19}F spectra were recorded (as indicated) on a Bruker 300 MHz or 400 MHz spectrometer at room temperature (25 °C) and are reported as chemical shifts (δ) in ppm relative to TMS (δ = 0). Spin multiplicities are reported as a singlet (s), doublet (d), triplet (t) and quartet (q) with coupling constants (*J*) given in Hz, or multiplet (m). Broad peaks are marked as br. ^{1}H and ^{13}C resonances were assigned with the aid of additional information from 1D and 2D NMR spectra (H,H-NOESY, H,H-COSY, DEPT-135, HSQC and HMBC). ESI-HRMS was measured on Xevo G2-S Tof (Waters). All mass data are reported as mass-per-charge ratio *m/z* (intensity in %, [assignment]).

Abbreviations: EF: Electric field; EFC: Electric-field catalysis; I: Current; P: Product; RT: Room temperature; SM: Starting material; TBA: Tetrabutyl ammonium; THF: Tetrahydrofuran.

2. Synthesis

2.1. Synthesis of Diepoxides, Triepoxides and Tetraepoxides

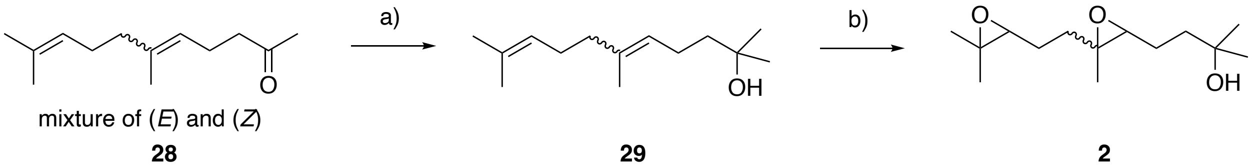

Scheme S1. (a) MeMgBr, Et_2O, 0 °C to rt, 1.5 h, 92%; (b) *m*-CPBA, $NaHCO_3$, CH_2Cl_2, 0 °C to rt, 15 min, 87%.

Diepoxide 2 was prepared following the previously reported procedure.[S2]

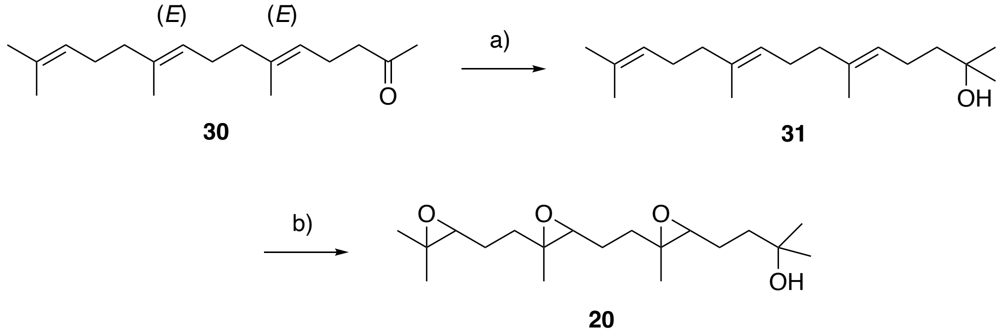

Scheme S2. (a) MeMgBr, Et_2O, 0 °C to rt, 1.5 h, 92%; (b) *m*-CPBA, $NaHCO_3$, CH_2Cl_2, 0 °C to rt, 15 min, 77%.

Triepoxide 20 was prepared following the previously reported procedure.[S2]

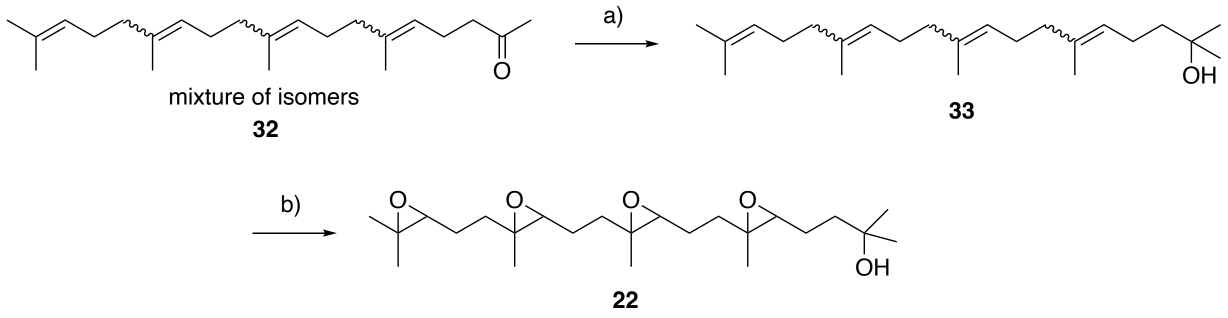

Scheme S3. (a) MeMgBr, Et_2O, 0 °C to rt, 1.5 h, 99%; (b) *m*-CPBA, $NaHCO_3$, CH_2Cl_2, 0 °C to rt, 15 min, 69%.

Tetraepoxide 22 was prepared following the previously reported procedure.[S2]

2.2. Synthesis of Thiourea Ion Pair Breakers

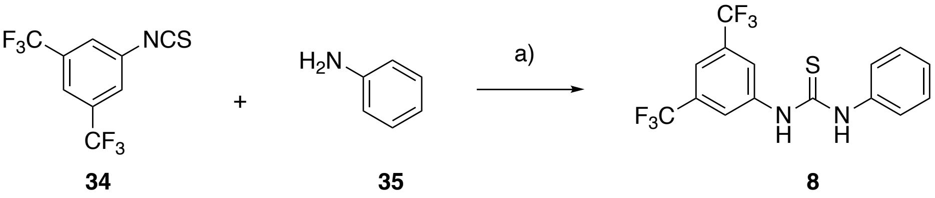

Scheme S4. (a) THF, 0 ºC to rt, overnight, 87%.

Thiourea 8 was prepared following the previously reported procedure.[S3]

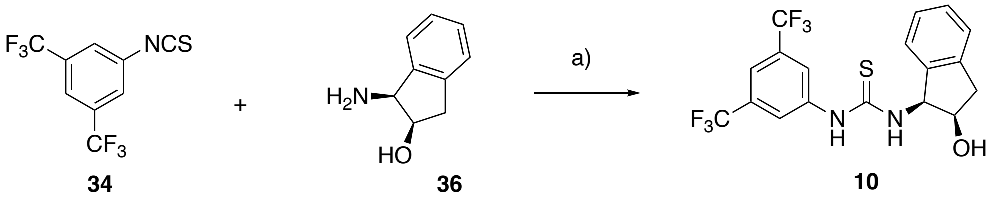

Scheme S5. (a) THF, 0 ºC to rt, overnight, 77%.

Thiourea 10 was prepared following the previously reported procedure.[S4]

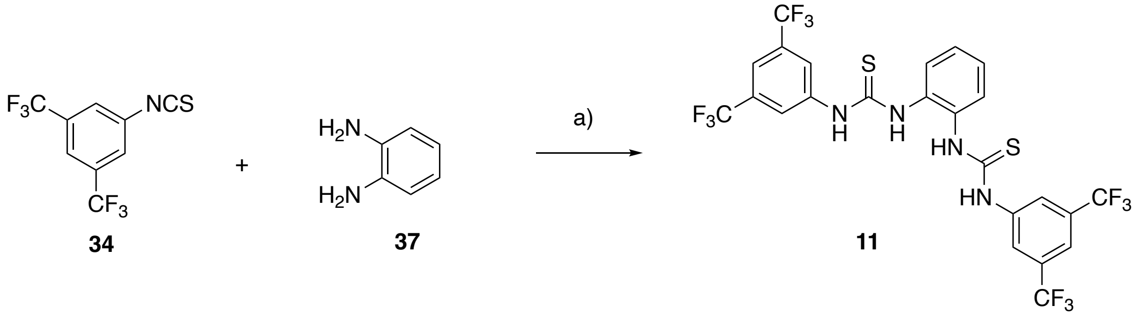

Scheme S6. (a) THF, 0 ºC to rt, overnight, 73%.

Thiourea 11 was prepared following the previously reported procedure.[S5]

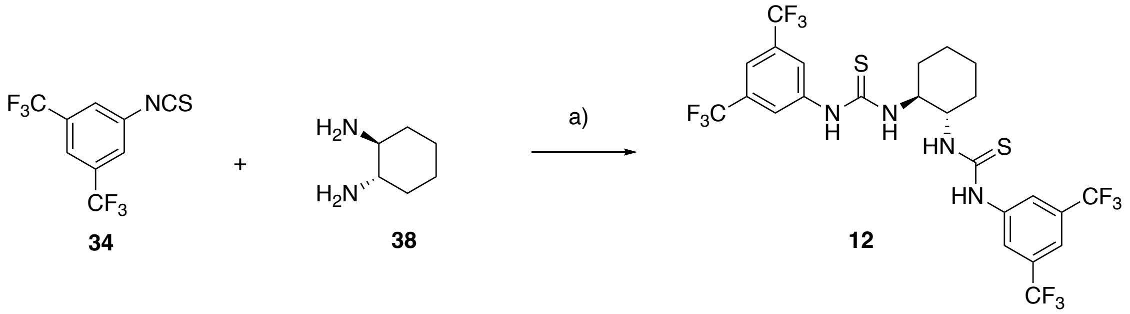

Scheme S7. (a) THF, 0 ºC to rt, overnight, 55%.

Thiourea 12 was prepared following the previously reported procedure.[S6]

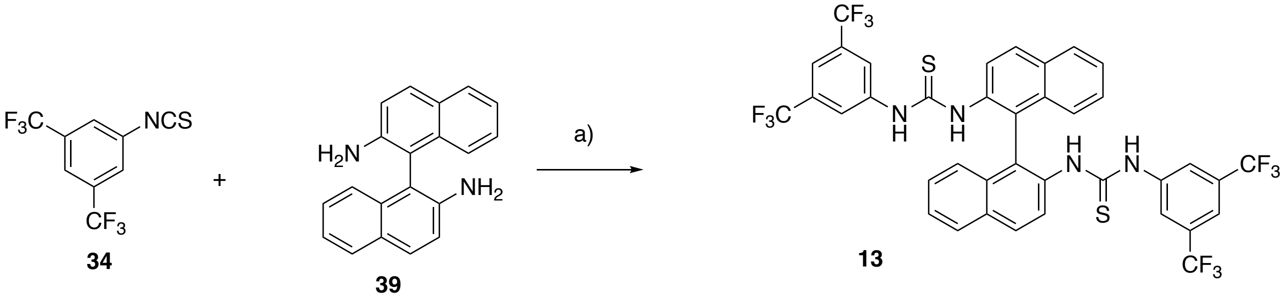

Scheme S8. (a) THF, 0 ºC to rt, overnight, 49%.

Thiourea 13 was prepared following the previously reported procedure.[S7]

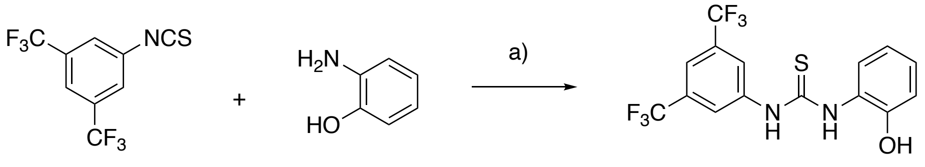

Scheme S9. (a) THF, 0 ºC to rt, overnight, 84%.

Thiourea 14. To a stirred solution of **40** (116 mg, 1.06 mmol) in THF (1.5 mL) at 0 ºC was added **34** (194 µL, 1.06 mmol). Then, the ice-water bath was removed, and the reaction mixture was stirred overnight at rt. The resulting reaction mixture was concentrated under reduced pressure. The residue was then dissolved in CH_2Cl_2 (5 mL) and precipitated by the addition of pentane (20 mL). Finally, the crude product was filtered and washed, first with pentane (3 x 20 mL) and then with a mixture of pentane/CH_2Cl_2 4:1 (2 x 25 mL) to give **14** as a colorless solid (338 mg, 84%), which was used without further purification. IR (neat): 3279 (w, O-H), 3041 (m, ar C-H), 1557 (w, ar C-C), 1539 (w, ar C-C), 1499 (w, ar C-C), 1465 (w, ar C-C), 1384 (w), 1354 (w), 1309 (w), 1273 (s, ar C-F), 1173 (m, C-O), 1127 (s, C=S), 981 (m), 882 (m), 849 (w), 759 (m), 711 (m), 680 (m); ^{1}H NMR (300 MHz, $CDCl_3$): 7.95 (br s, 2H), 7.88 (br s, 1H), 7.76 (br s, 1H), 7.73 (br s, 1H), 7.39 – 7.26 (m, 2H), 7.09 (dd, $^3J_{HH}$ = 8.0, $^4J_{HH}$ = 0.9 Hz, 1H), 7.08 – 7.02 (m, 1H), 6.13 (br s, 1H); ^{13}C NMR (101 MHz, $CDCl_3$): 180.5 (C), 151.0 (C), 139.1 (C), 132.3 (q, $^2J_{CF}$ = 33.8 Hz, 2C), 130.4 (CH), 127.2 (CH), 124.9 (q, $^3J_{CF}$ = 4.0 Hz, 2CH), 122.8 (q, $^1J_{CF}$ = 272.9 Hz, 2C), 122.7 (C), 122.1 (CH), 120.0 (hept, $^3J_{CF}$ = 3.8 Hz, CH), 118.2 (CH); ^{19}F NMR (282 MHz, $CDCl_3$): -63.0 (6F); HRMS (ESI): calcd. for $[C_{15}H_{10}F_6N_2OS+H]^+$: 381.0491, found: 381.0488.

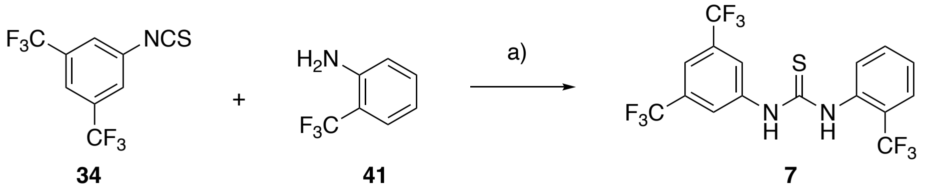

Scheme S10. (a) THF, 0 ºC to rt, overnight, 48%.

Thiourea 7. To a stirred solution of **41** (97 µL, 1.06 mmol) in THF (1.5 mL) at 0 ºC was added **34** (194 µL, 1.06 mmol). Then, the ice-water bath was removed, and the reaction mixture was stirred overnight at rt. The resulting reaction mixture was concentrated under reduced pressure. The residue was then dissolved in CH_2Cl_2 (5 mL) and precipitated by the addition of pentane (20 mL). Finally, the crude product was filtered and washed, first with pentane (3 x 20 mL) and then with a mixture of pentane/CH_2Cl_2 4:1 (2 x 25 mL) to give **7** as a colorless solid (222 mg, 48%), which was used without further purification. IR (neat): 3048 (w, ar C-H), 1527 (m, ar C-C), 1470 (w, ar C-C), 1385 (m), 1320 (m), 1274 (m, ar-C-F), 1161 (m), 1120 (w, C=S), 1061 (m), 1035 (m), 980 (m), 889 (m), 720 (m), 681 (m), 658 (m); ^{1}H NMR (300 MHz, $CDCl_3$): 7.94 (br s, 2H), 7.81 (d, $^{3}J_{HH}$ = 7.8 Hz, 1H), 7.75 (br s, 2H), 7.72 – 7.65 (m, 2H), 7.61 (br s, 1H), 7.57 – 7.49 (m, 1H); ^{13}C NMR (101 MHz, $CDCl_3$): 180.7 (C), 138.9 (C), 134.0 (C), 133.5 (CH), 132.6 (q, $^{2}J_{CF}$ = 34.0 Hz, 2C), 129.4 (CH), 128.5 (CH), 127.5 (q, $^{3}J_{CF}$ = 5.0 Hz, CH), 126.6 (q, $^{2}J_{CF}$ = 33.6 Hz, C), 125.0 (q, $^{3}J_{CF}$ = 3.1 Hz, 2CH), 123.1 (q, $^{1}J_{CF}$ = 273.2 Hz, C), 122.7 (q, $^{1}J_{CF}$ = 272.9 Hz, 2C), 120.2 (hept, $^{3}J_{CF}$ = 3.8 Hz, CH); ^{19}F NMR (282 MHz, $CDCl_3$): -61.4 (3F), -63.0 (6F); HRMS (ESI): calcd. for $[C_{16}H_9F_9N_2S+H]^+$: 433.0416, found: 433.0407.

3. Electric-Field Catalysis

3.1. Microfluidic Capacitors

Electric-field catalysis studies were performed using a setup similar to those described in reference[S1] using a stand-alone Vapourtec Ion Electrochemical Reactor, with an Aim-TTi EX354RD Dual Power Supply from Thurlbym Thandar Instruments Ltd. Chemyx Fusion 100 Touch Syringe Pumps were used in the flow set-ups. Electrode materials employed were rigid graphite foil (Gr, Goodfellow, 99.95%, 1.0 mm thickness). The electrodes (5 x 5 cm^2) were separated by a 0.25 mm FEP spacer, resulting in a reactor volume of 0.3 mL, with an exposed electrode surface area of 12 cm^2.

The graphite plates were polished using a Presi Le Cube polishing machine, in sequential steps: 5 minutes with 6 µm abrasive paper and diamond suspension, followed by 3 minutes with 3 µm, 3 minutes with 1 µm, and finally 5 minutes with 1/4 µm particles.

After every reaction was completed, the system was flushed with chloroform (5.0 mL) at a flow rate of 0.4 mL/min. The electrodes and FEP spacer were then cleaned with acetone. The graphite electrodes were further immersed in chloroform overnight, followed by 30 minutes of sonication. Before reuse, the electrodes were repolished to restore surface quality.

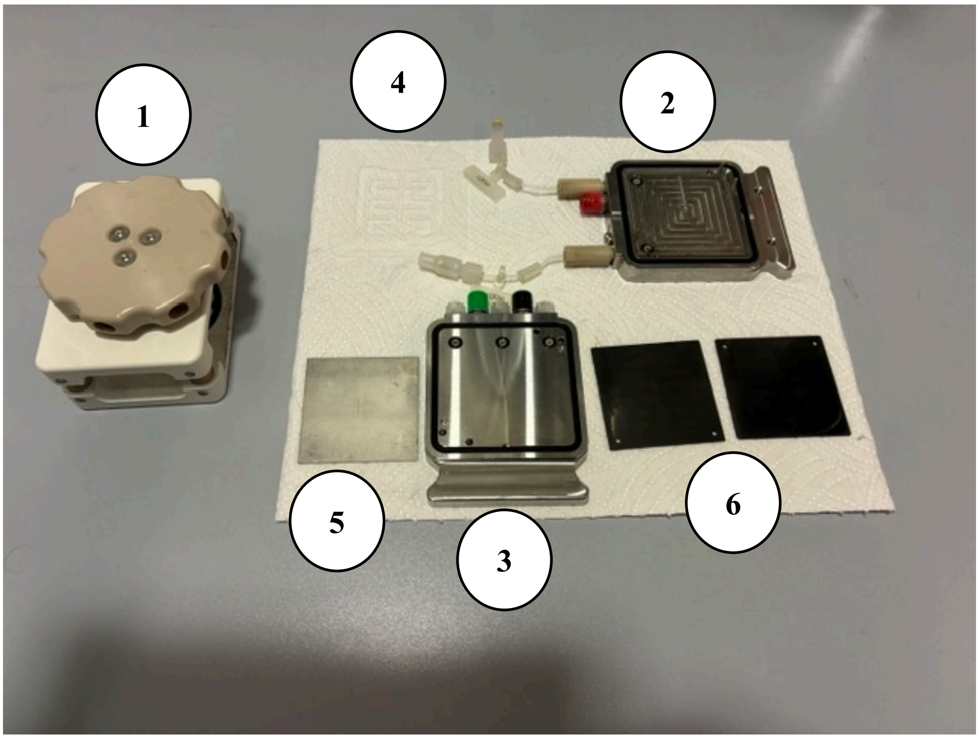

Figure S1. Disassembled microfluidic capacitor. 1. External housing. 2. Metallic bottom electrode holder. 3. Metallic top electrode holder. 4. 0.25 mm FEP spacer. 5. Metallic spacer. 6. Graphite electrodes.

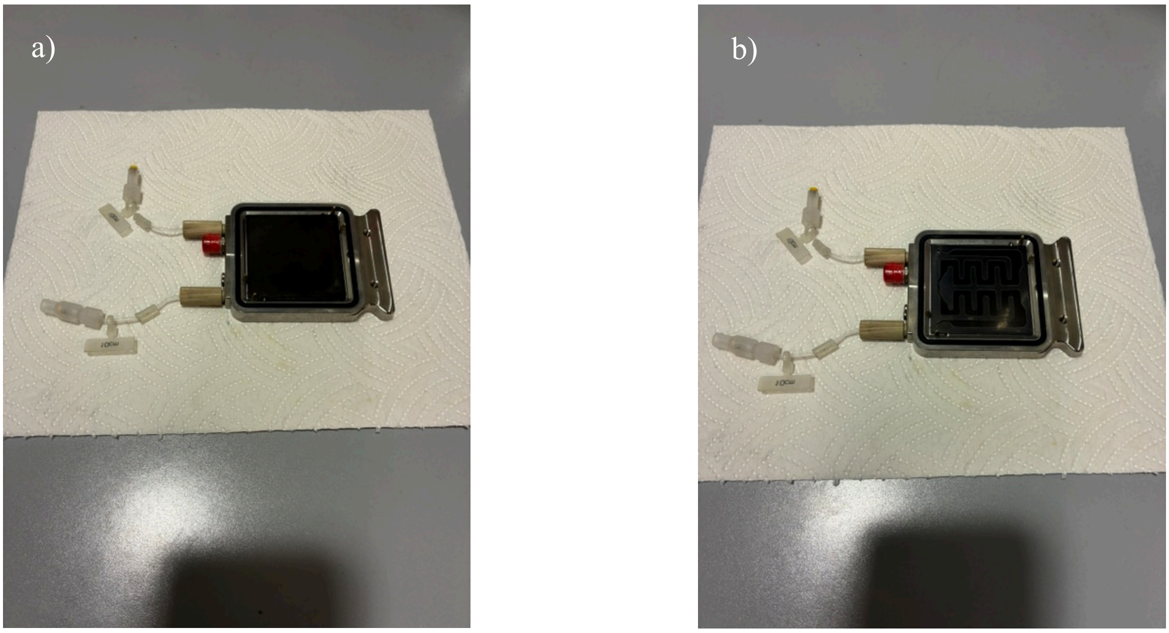

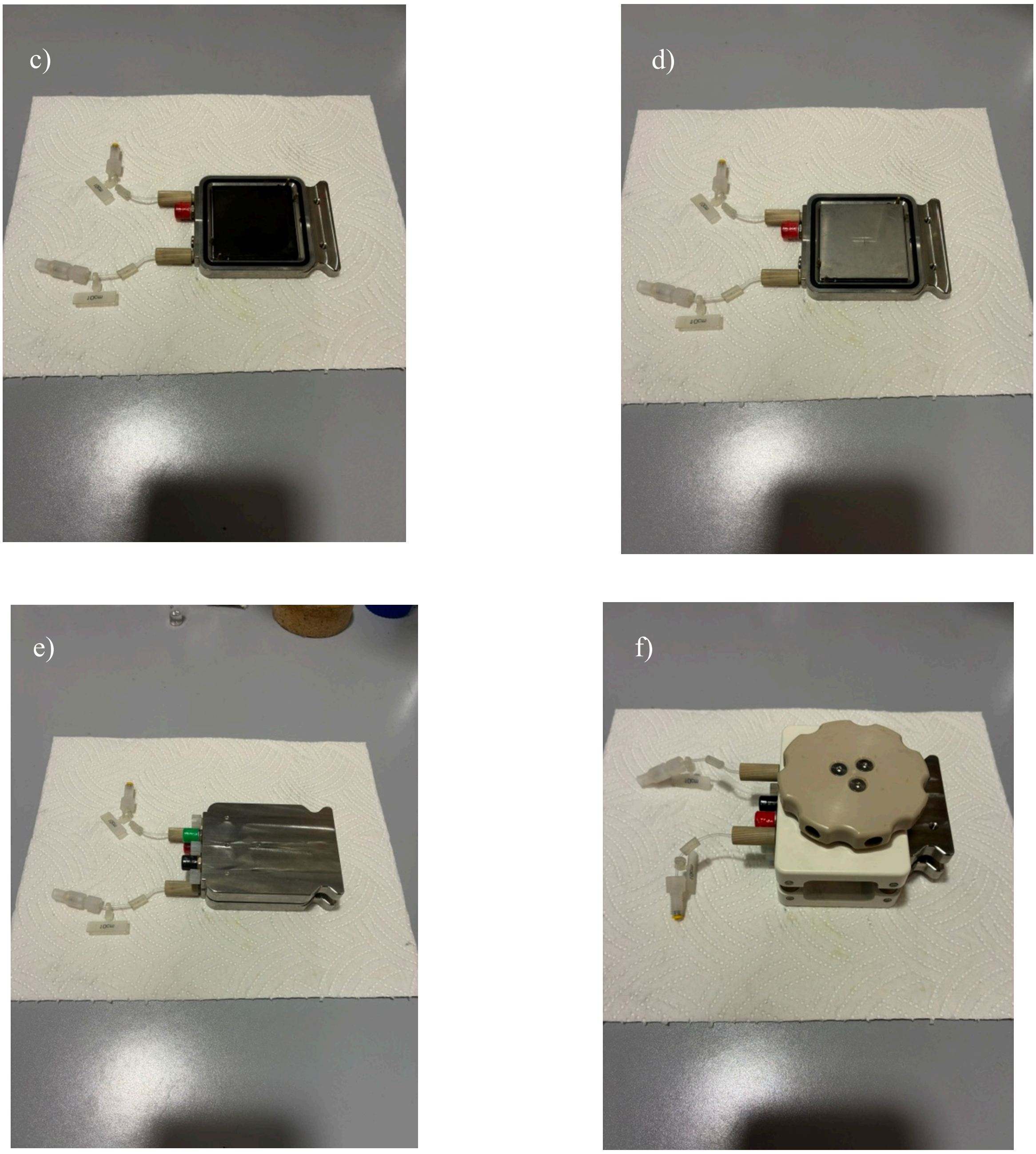

Figure S2. Assembly of the microfluidic capacitor. a) One of the graphite electrodes is placed in the metallic bottom electrode holder. b) The 0.25 mm FEP spacer is fitted on top of the graphite electrode. c) The second graphite electrode is placed on the FEP spacer. d) The metallic spacer is placed on top of the second graphite electrode. e) The metallic top electrode holder is fitted with the bottom part. f) The metallic unit is placed inside the housing, and pressure is applied until the capacitor is sealed.

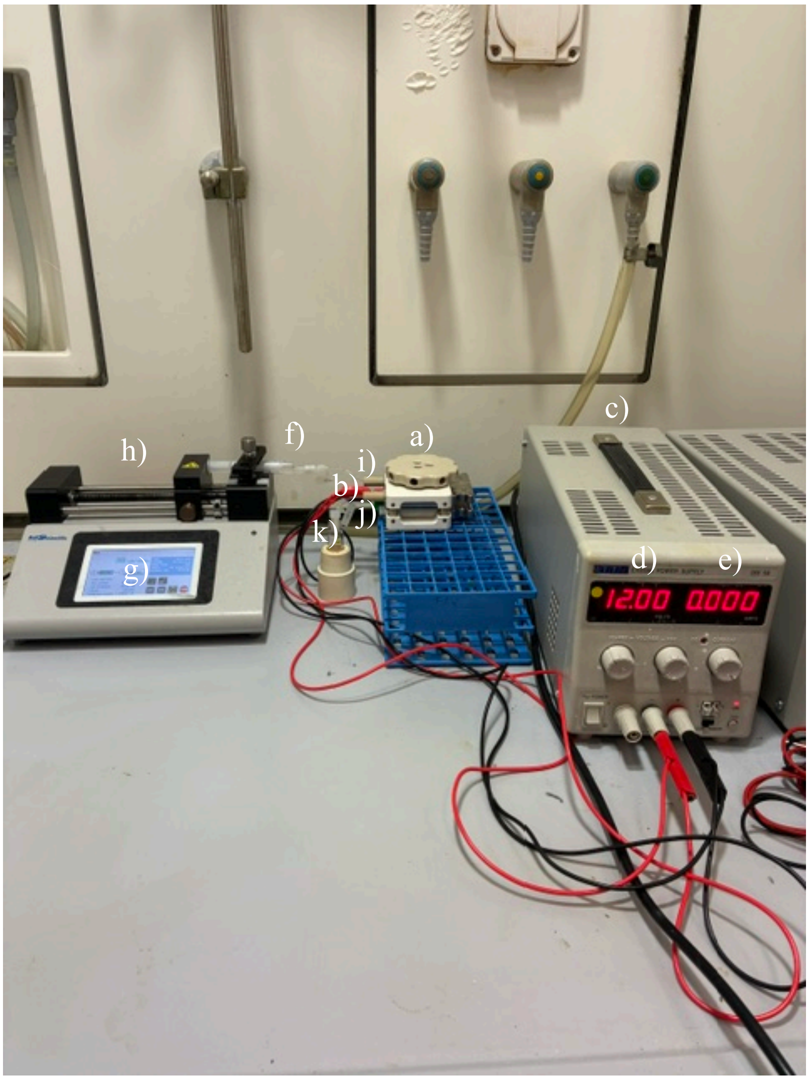

Figure S3. The microfluidic capacitor at work in simplest possible configuration. Once assembled, the (a) microfluidic capacitor is (b) connected to (c) a power supply, where (d) the voltage is set and (e) the current is recorded. The (f) reaction mixture of interest in the solvent of interest is injected at (g) a set flowrate using (h) a syringe pump, into (i) the microfluidic capacitor. At (j) the solution exit from the microdluidic capacitor, where a (k) vial is placed to collect the reaction mixture for analysis. The reaction solution is pumped through the capacitor at a fixed speed and applied voltage. The example shows one of the reactions at V = 12 V reported in Figure 2B, with I = 0.000 A measured at steady state during the reaction, supporting that parasitic faradaic processes are not involved, and thus the occurrence of electric-field catalysis.

3.2. General Procedure for Diepoxide Cyclization

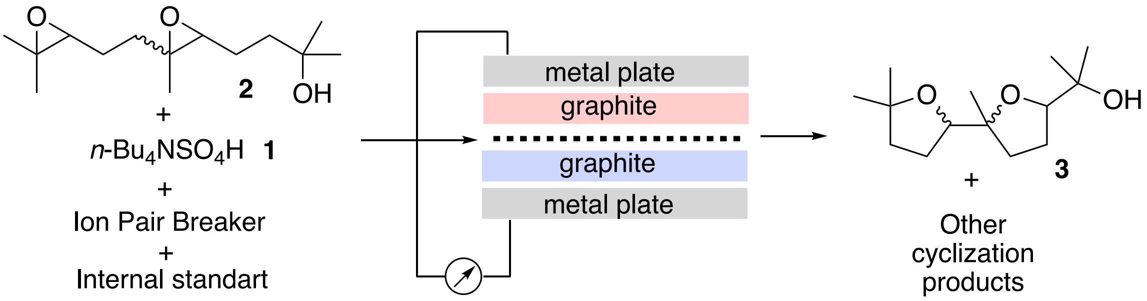

Scheme S11. Microfluidic EF catalyzed intramolecular cyclization of diepoxide **2**.

A solution containing substrate **2** (153 mM), mesitylene as internal standard (IS, 153 mM), *n*-Bu_4NSO_4H **1** (0.25 mol%) and (if it is used) ion pair breaker (0.25 mol%) in $CDCl_3$, was loaded in a syringe. The solution was infused at 30 µL/min using a syringe pump into the microfluidic capacitor under constant voltage. The initial 0.45 mL, corresponding to one and a half reactor volumes, was discarded before collecting each sample to ensure that a steady state of the system had been reached at the desired voltage. Then, 150 µL samples were collected over 5 min. Samples were diluted with additional $CDCl_3$ and the ^1H NMR spectrum was recorded right after every experiment. Yields (η_m) were determined by comparing the integrals of the epoxide signal to that of the IS. All experiments were conducted under $I = 0.000$ A.

The resulting dependence of the microfluidic yield (Y_m) on the applied voltage (V) was plotted as YV curves and fitted with Equation (S1) to retrieve the half effective voltage (V_{50}), the gating coefficient (n_g), and the theoretical maximum yield (Y_m^{∞}).

$$Y_m = Y_m^{\infty} / (1 + (V_{50} / V)^{n_g}) \qquad \text{(S1)}$$

Critical voltages V_c were estimated from the fit curve as the voltage at which $\eta_m = 10\%$ was observed.

Diepoxide contained about 12% of cyclization product, which was not considered in the reported EFC reaction yields. A small amount of residual CH_2Cl_2 also remained in the epoxide substrates.

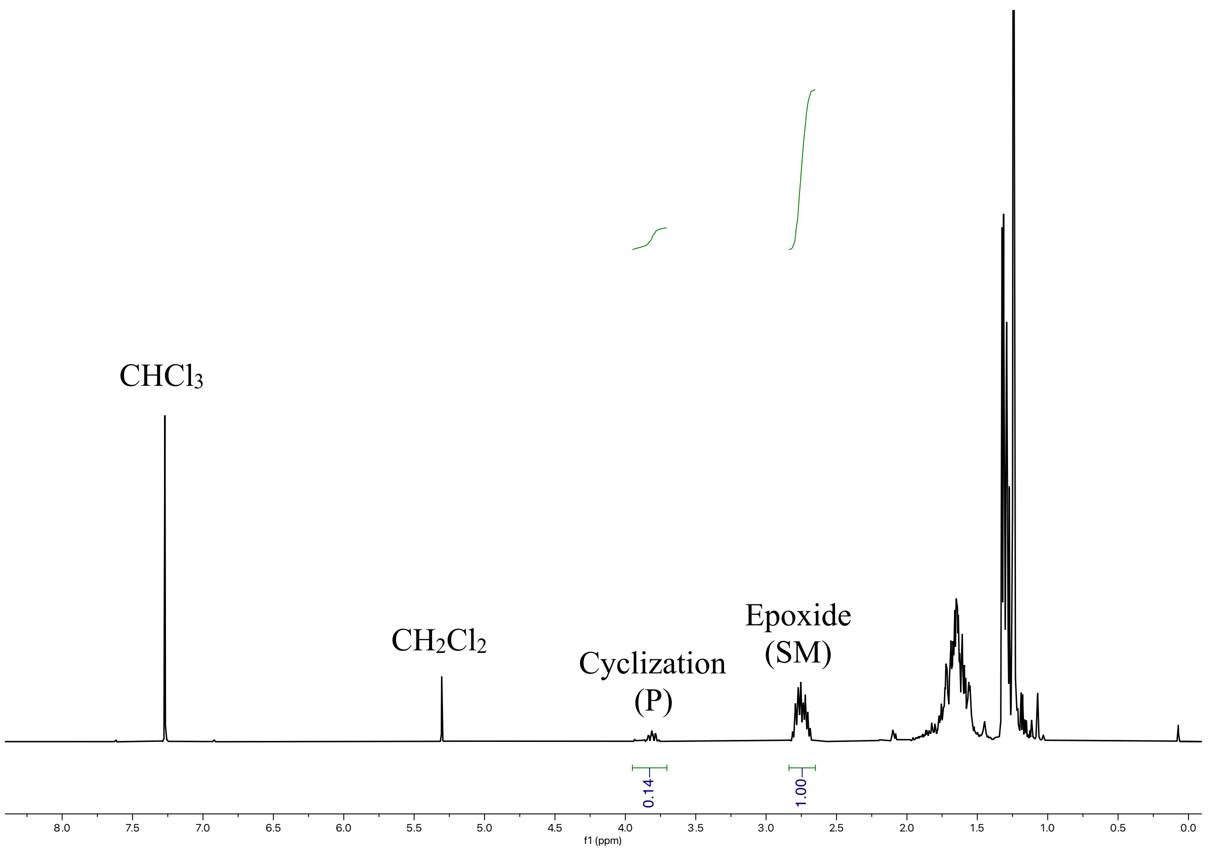

Figure S4. ^{1}H NMR spectrum of compound **2** in $CDCl_3$.

3.3. Voltage Dependence of Diepoxide Cyclizations without Ion-Pair Breakers

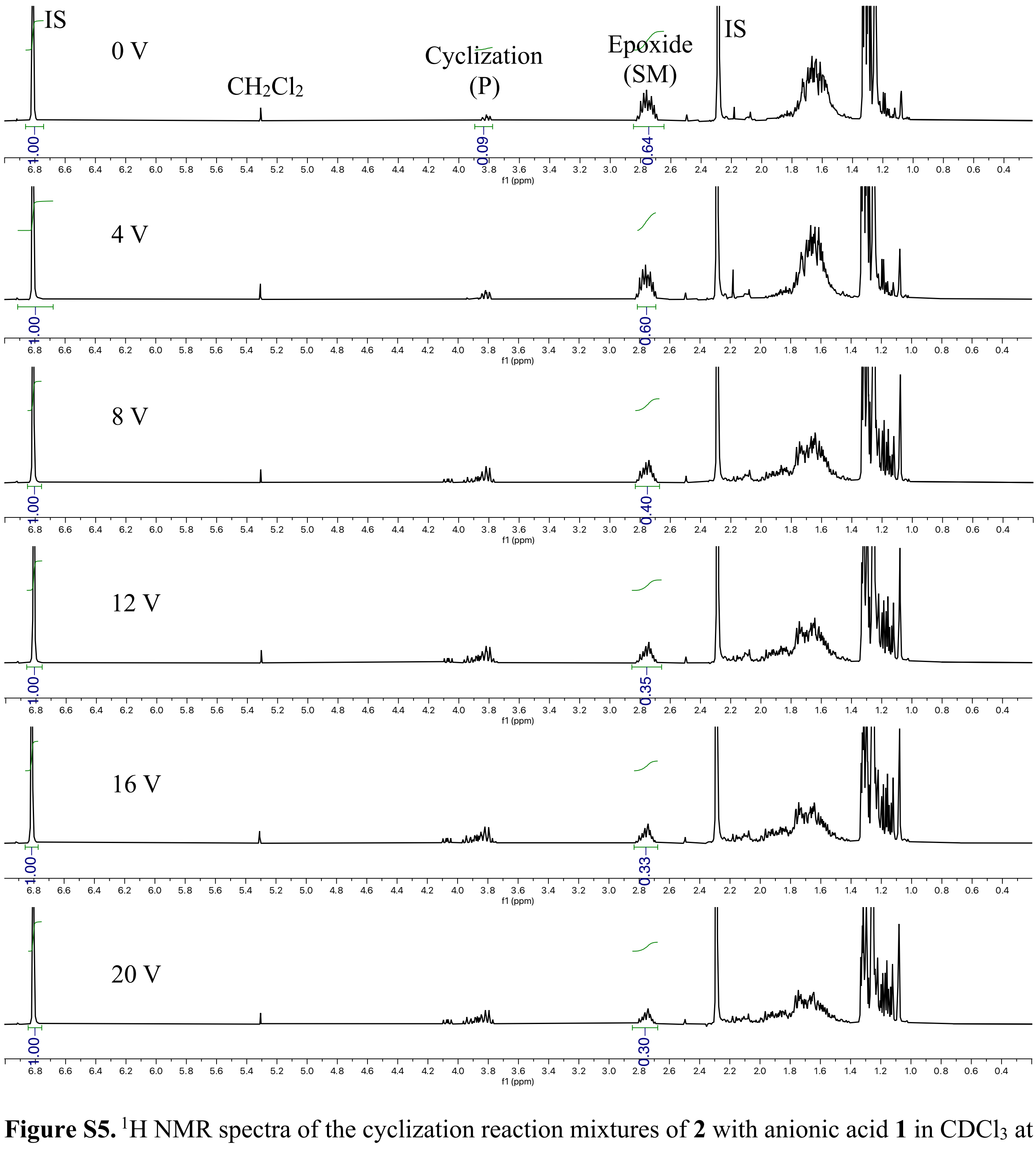

Figure S5. ^{1}H NMR spectra of the cyclization reaction mixtures of **2** with anionic acid **1** in $CDCl_3$ at rt in microfluidic capacitors (30 µL/min) with different voltages applied.

Table S1. EFC with substrate **2**, anionic acid **1** and without ion-pair breakers.[a]

Entry	S (mM)[b]	C (mol%)[c]	B (mol%)[d]	*V* (V)[e]	*I* (A)[f]	Y_m (%)[g]
1	153	0.25	0	0	0.000	0
2	153	0.25	0	4	0.000	6
3	153	0.25	0	8	0.000	27
4	153	0.25	0	12	0.000	44
5	153	0.25	0	16	0.000	49
6	153	0.25	0	20	0.000	54

[a]In microfluidic capacitor in $CDCl_3$, rt, flowrate 30 µL/min. [b]Concentration of substrate **2**, in millimolar. [c]Concentration of catalyst **1**, in mol% of substrate. [d]Concentration of ion-pair breaker, in mol% of substrate. [e]Applied voltage, in volts. [f]Current measured, in amperes. [g]Microfluidic yield of total cyclization products, in percent.

Y_m^{max} = 54% V_c = 5.0 V

n_g = 3.3

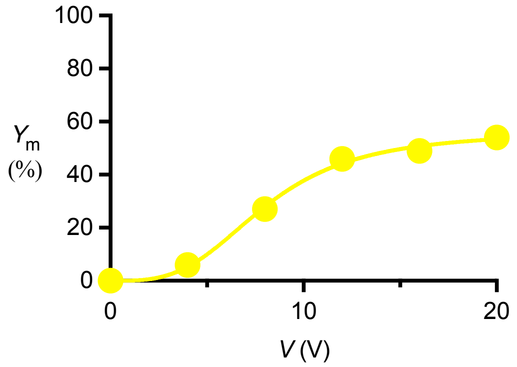

Figure S6. *YV* Curve for **3** obtained with anionic acid **1** in $CDCl_3$, with Y_m^{max} as maximal microfluidic yield, V_c as critical voltage and n_g as gating coefficient.

3.4. Voltage Dependence of Diepoxide Cyclizations with Ion-Pair Breakers

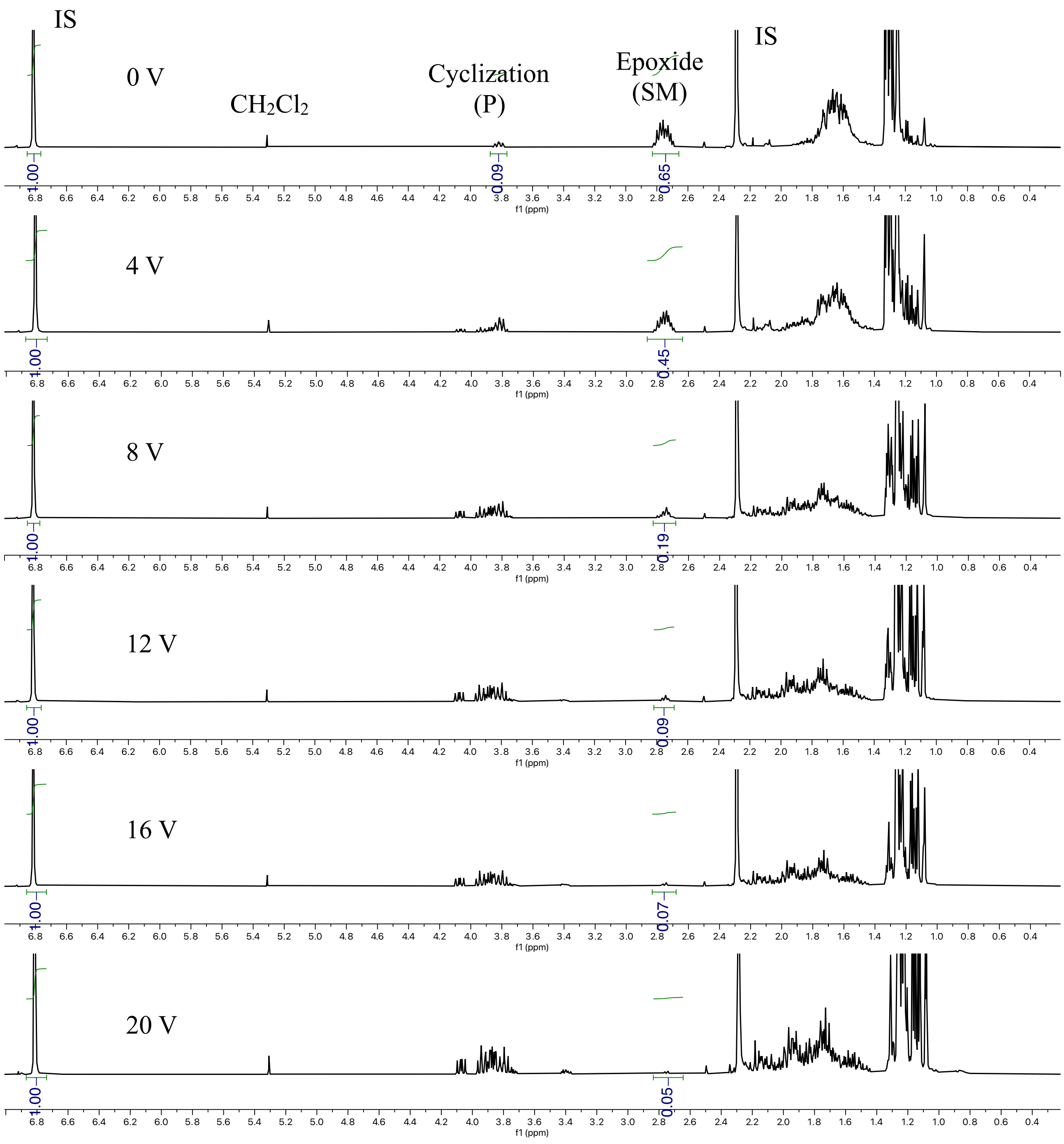

Figure S7. ^{1}H NMR spectra of the cyclization reaction mixtures of **2** under EFC with anionic acid **1** and thiourea **6** in $CDCl_3$ at different voltages.

Table S2. EFC with substrate **2**, anionic acid **1** and with thiourea **6**.[a]

Entry	S (mM)[b]	C (mol%)[c]	B (mol%)[d]	V (V)[e]	I (A)[f]	Y_m (%)[g]
1	153	0.25	0.25	0	0.000	0
2	153	0.25	0.25	4	0.000	31
3	153	0.25	0.25	8	0.000	71
4	153	0.25	0.25	12	0.000	86
5	153	0.25	0.25	16	0.000	89
6	153	0.25	0.25	20	0.000	92

[a]In microfluidic capacitor in $CDCl_3$, rt, flowrate 30 µL/min. [b]Concentration of substrate **2**, in millimolar. [c]Concentration of catalyst **1**, in mol% of substrate. [d]Concentration of ion-pair breaker, in mol% of substrate. [e]Applied voltage, in volts. [f]Current measured, in amperes. [g]Microfluidic yield of total cyclization products, in percent.

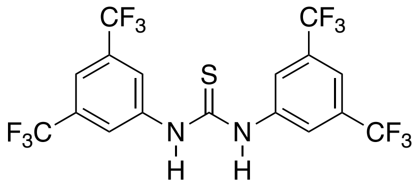

6

Y_m^{max} = 92% V_c = 2.3 V

n_g = 2.6

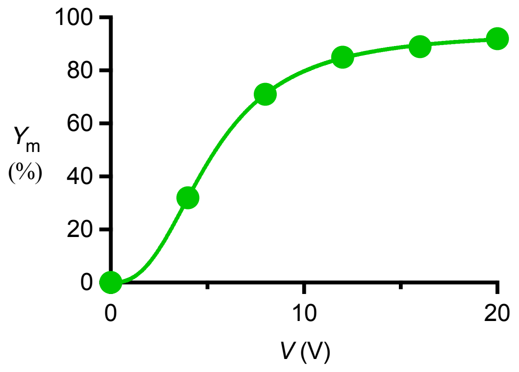

Figure S8. Yield (Y_m) of **3** under EFC with anionic acid **1** and thiourea **6** in $CDCl_3$ as a function of the voltage. With Y_m^{max} as maximum microfluidic yield, V_c as critical voltage and n_g as gating coefficient.

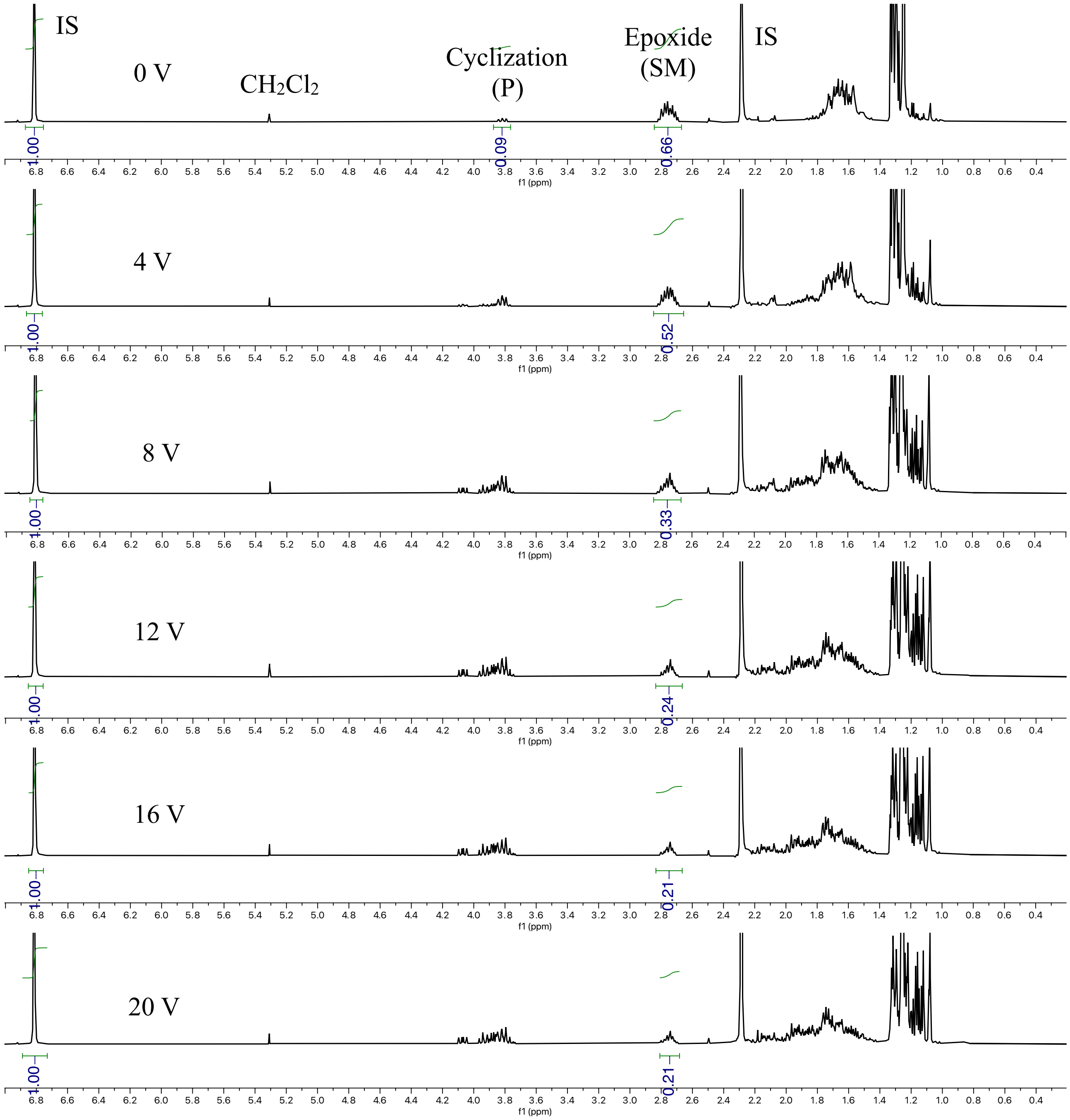

Figure S9. ^{1}H NMR spectra of the cyclization reaction of **2** under EFC with anionic acid **1** and thiourea **7** in $CDCl_3$ at different voltages.

Table S3. EFC with substrate **2**, anionic acid **1** and thiourea **7**.[a]

Entry	S (mM)[b]	C (mol%)[c]	B (mol%)[d]	V (V)[e]	I (A)[f]	Y_m (%)[g]
1	153	0.25	0.25	0	0.000	0
2	153	0.25	0.25	4	0.000	22
3	153	0.25	0.25	8	0.000	50
4	153	0.25	0.25	12	0.000	64
5	153	0.25	0.25	16	0.000	69
6	153	0.25	0.25	20	0.000	69

[a]In microfluidic capacitor in $CDCl_3$, rt, flowrate 30 µL/min. [b]Concentration of substrate **2**, in millimolar. [c]Concentration of catalyst **1**, in mol% of substrate. [d]Concentration of ion-pair breaker, in mol% of substrate. [e]Applied voltage, in volts. [f]Current measured, in amperes. [g]Microfluidic yield of total cyclization products, in percent.

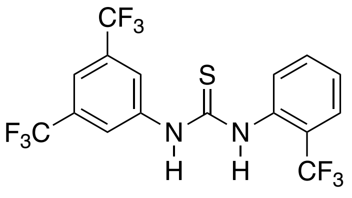

7

$Y_m^{max} = 69\%$ $V_c = 2.7$ V

$n_g = 2.5$

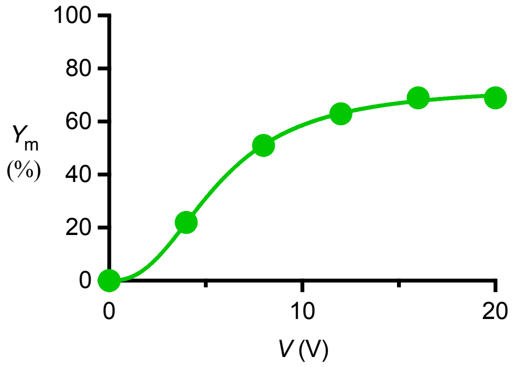

Figure S10. Yield (Y_m) of **3** under EFC with anionic acid **1** and thiourea **7** in $CDCl_3$ as a function of the voltage. With Y_m^{max} as maximum microfluidic yield, V_c as critical voltage and n_g as gating coefficient.

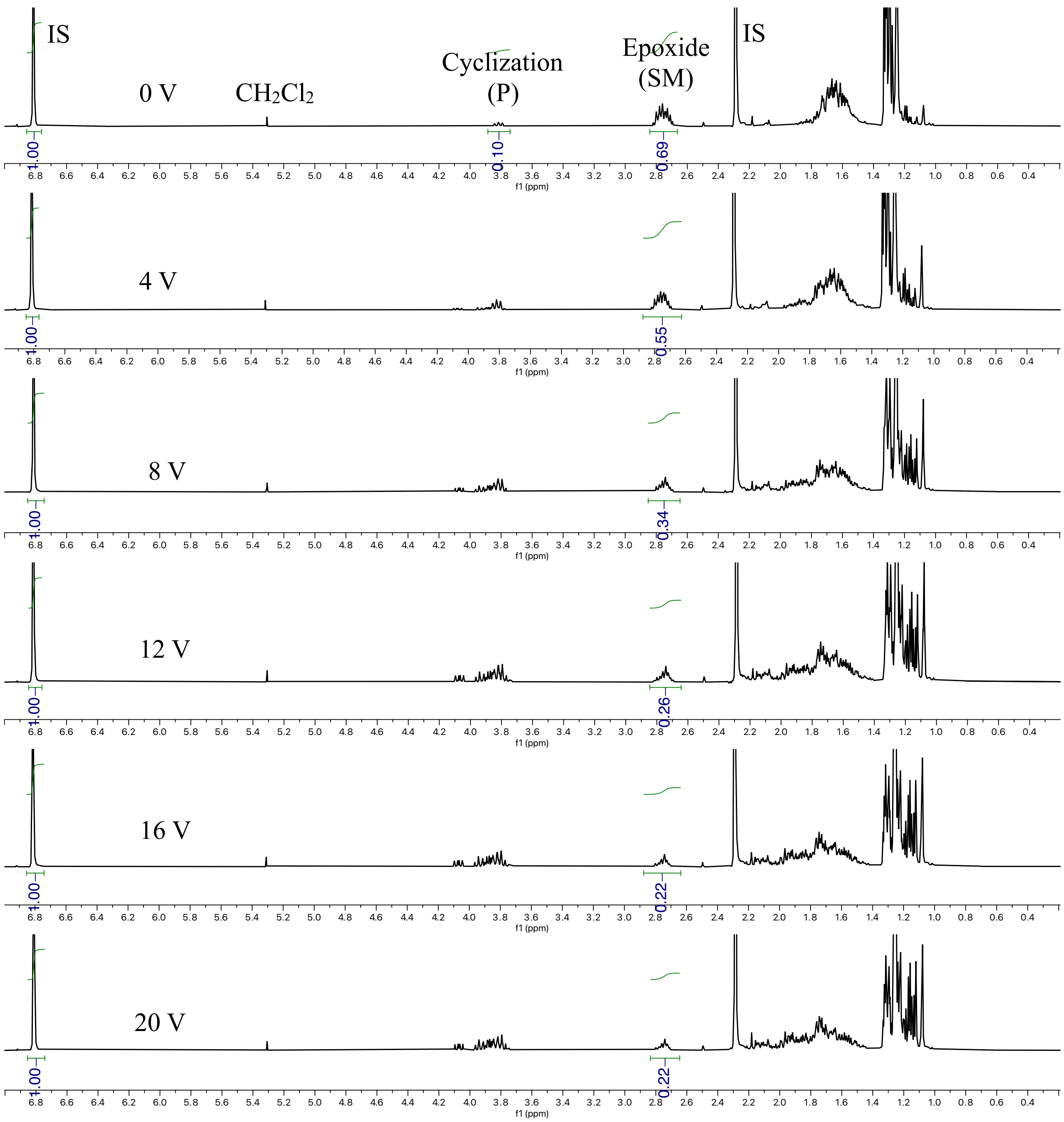

Figure S11. ^{1}H NMR spectra of the cyclization reaction of **2** under EFC with anionic acid **1** and thiourea **8** in $CDCl_3$ at different voltages.

Table S4. EFC with substrate **2**, anionic acid **1** and thiourea **8**.[a]

Entry	S (mM)[b]	C (mol%)[c]	B (mol%)[d]	V (V)[e]	I (A)[f]	Y_m (%)[g]
1	153	0.25	0.25	0	0.000	0
2	153	0.25	0.25	4	0.000	21
3	153	0.25	0.25	8	0.000	49
4	153	0.25	0.25	12	0.000	62
5	153	0.25	0.25	16	0.000	68
6	153	0.25	0.25	20	0.000	69

[a]In microfluidic capacitor in $CDCl_3$, rt, flowrate 30 µL/min. [b]Concentration of substrate **2**, in millimolar. [c]Concentration of catalyst **1**, in mol% of substrate. [d]Concentration of ion-pair breaker, in mol% of substrate. [e]Applied voltage, in volts. [f]Current measured, in amperes. [g]Microfluidic yield of total cyclization products, in percent.

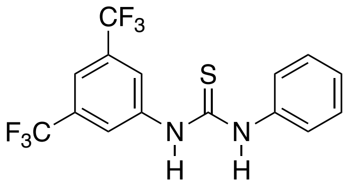

8

$Y_m^{max} = 69\%$ $V_c = 2.7$ V

$n_g = 2.4$

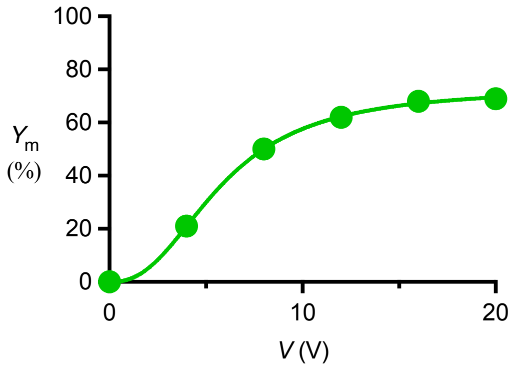

Figure S12. Yield (Y_m) of **3** under EFC with anionic acid **1** and thiourea **8** in $CDCl_3$ as a function of the voltage. With Y_m^{max} as maximum microfluidic yield, V_c as critical voltage and n_g as gating coefficient.

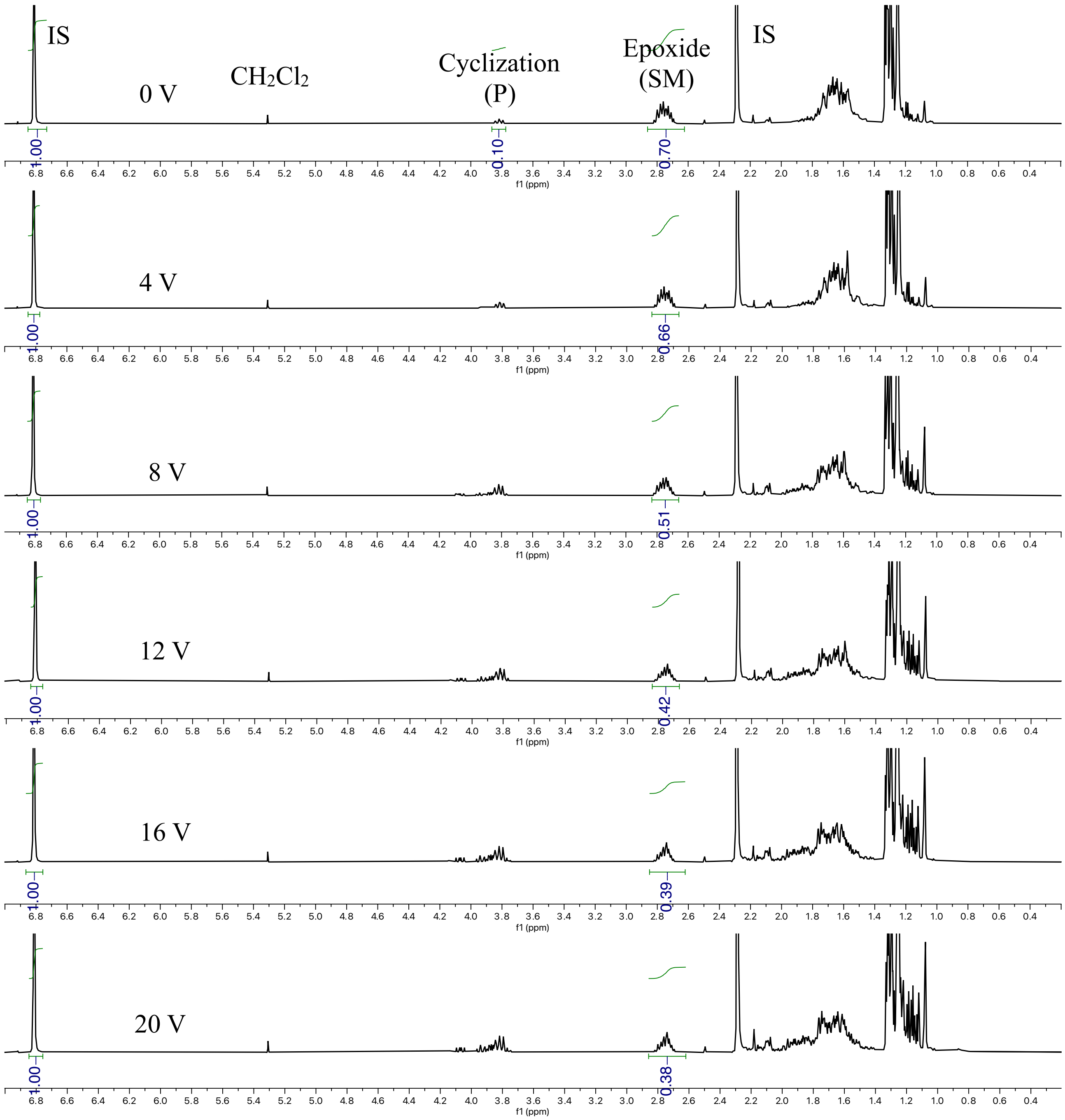

Figure S13. ^{1}H NMR spectra of the cyclization reaction of **2** under EFC with anionic acid **1** and thiourea **9** in $CDCl_3$ at different voltages.

Table S5. EFC with substrate **2**, anionic acid **1** and thiourea **9**.[a]

Entry	S (mM)[b]	C (mol%)[c]	B (mol%)[d]	*V* (V)[e]	*I* (A)[f]	Y_m (%)[g]
1	153	0.25	0.25	0	0.000	0
2	153	0.25	0.25	4	0.000	6
3	153	0.25	0.25	8	0.000	27
4	153	0.25	0.25	12	0.000	40
5	153	0.25	0.25	16	0.000	45
6	153	0.25	0.25	20	0.0000	46

[a]In microfluidic capacitor in $CDCl_3$, rt, flowrate 30 μL/min. [b]Concentration of substrate **2**, in millimolar. [c]Concentration of catalyst **1**, in mol% of substrate. [d]Concentration of ion-pair breaker, in mol% of substrate. [e]Applied voltage, in volts. [f]Current measured, in amperes. [g]Microfluidic yield of total cyclization products, in percent.

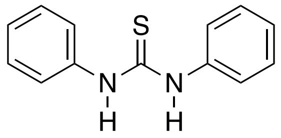

9

$Y_m^{max} = 46\%$ $V_c = 5.3$ V

$n_g = 3.6$

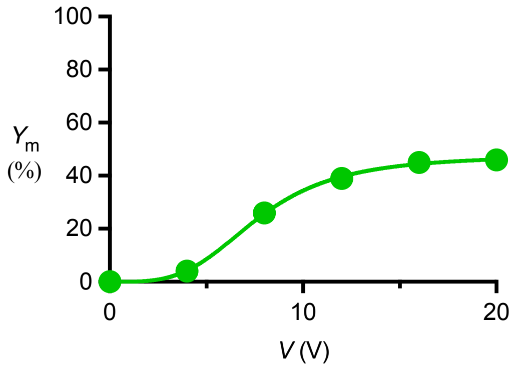

Figure S14. Yield (Y_m) of **3** under EFC with anionic acid **1** and thiourea **9** in $CDCl_3$ as a function of the voltage. With Y_m^{max} as maximum microfluidic yield, V_c as critical voltage and n_g as gating coefficient.

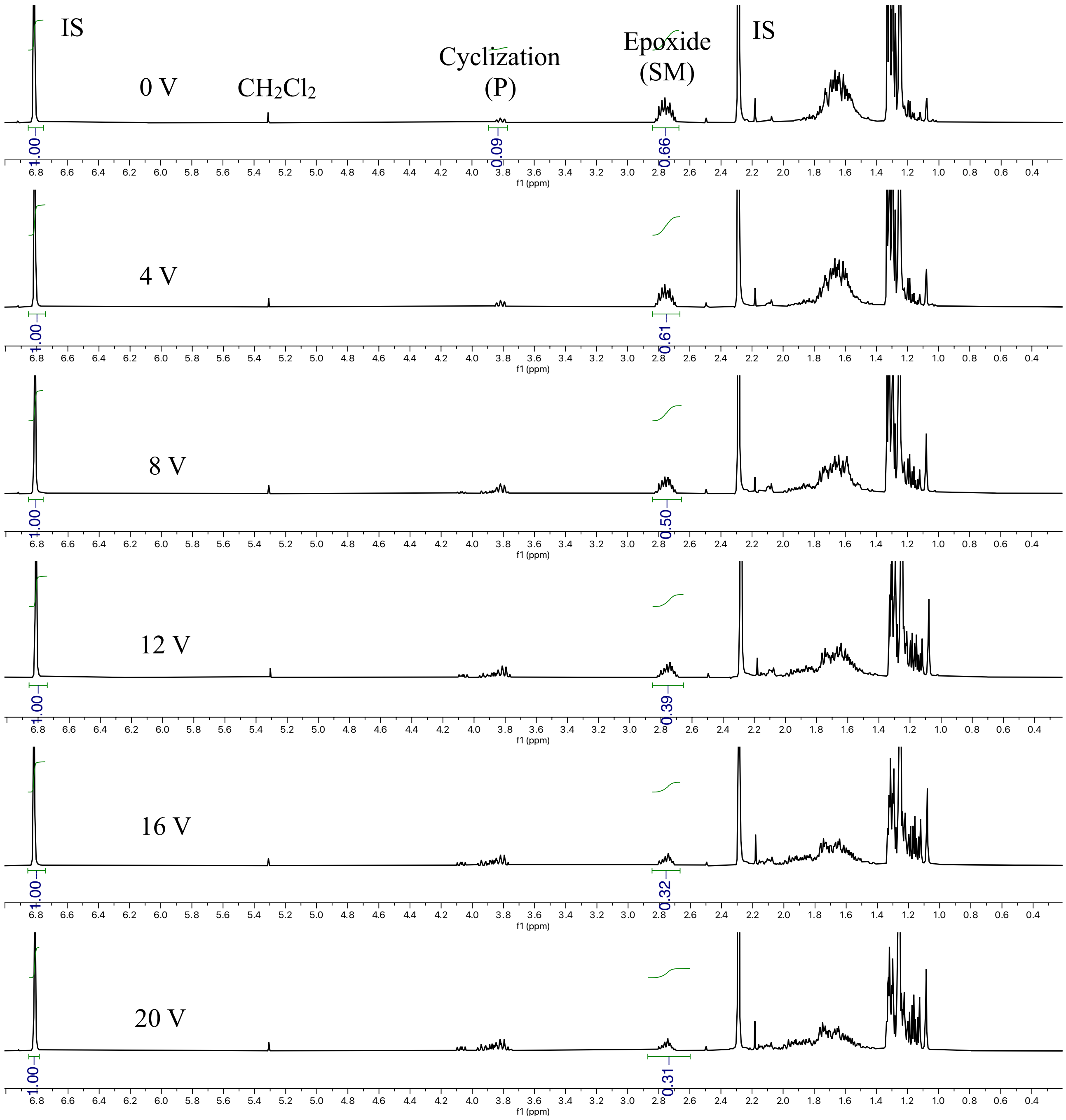

Figure S15. ^{1}H NMR spectra of the cyclization reaction of **2** under EFC with anionic acid **1** and thiourea **10** in $CDCl_3$ at different voltages.

Table S6. EFC with substrate **2**, anionic acid **1** and thiourea **10**.[a]

Entry	S (mM)[b]	C (mol%)[c]	B (mol%)[d]	V (V)[e]	I (A)[f]	Y_m (%)[g]
1	153	0.25	0.25	0	0.000	0
2	153	0.25	0.25	4	0.000	8
3	153	0.25	0.25	8	0.000	24
4	153	0.25	0.25	12	0.000	41
5	153	0.25	0.25	16	0.000	51
6	153	0.25	0.25	20	0.000	53

[a]In microfluidic capacitor in $CDCl_3$, rt, flowrate 30 µL/min. [b]Concentration of substrate **2**, in millimolar. [c]Concentration of catalyst **1**, in mol% of substrate. [d]Concentration of ion-pair breaker, in mol% of substrate. [e]Applied voltage, in volts. [f]Current measured, in amperes. [g]Microfluidic yield of total cyclization products, in percent.

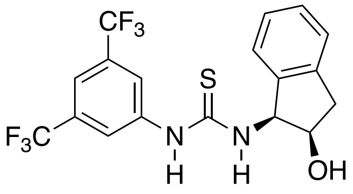

10

Y_m^{max} = 53% V_c = 4.8 V

n_g = 2.5

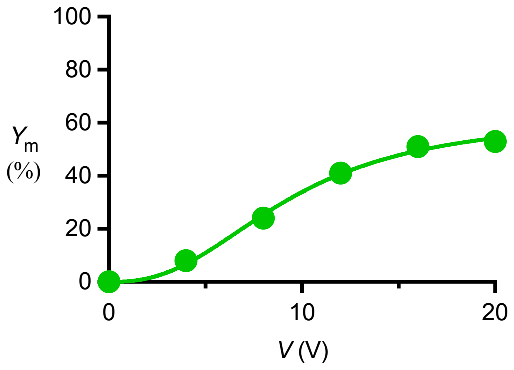

Figure S16. Yield (Y_m) of **3** under EFC with anionic acid **1** and thiourea **10** in $CDCl_3$ as a function of the voltage. With Y_m^{max} as maximum microfluidic yield, V_c as critical voltage and n_g as gating coefficient.

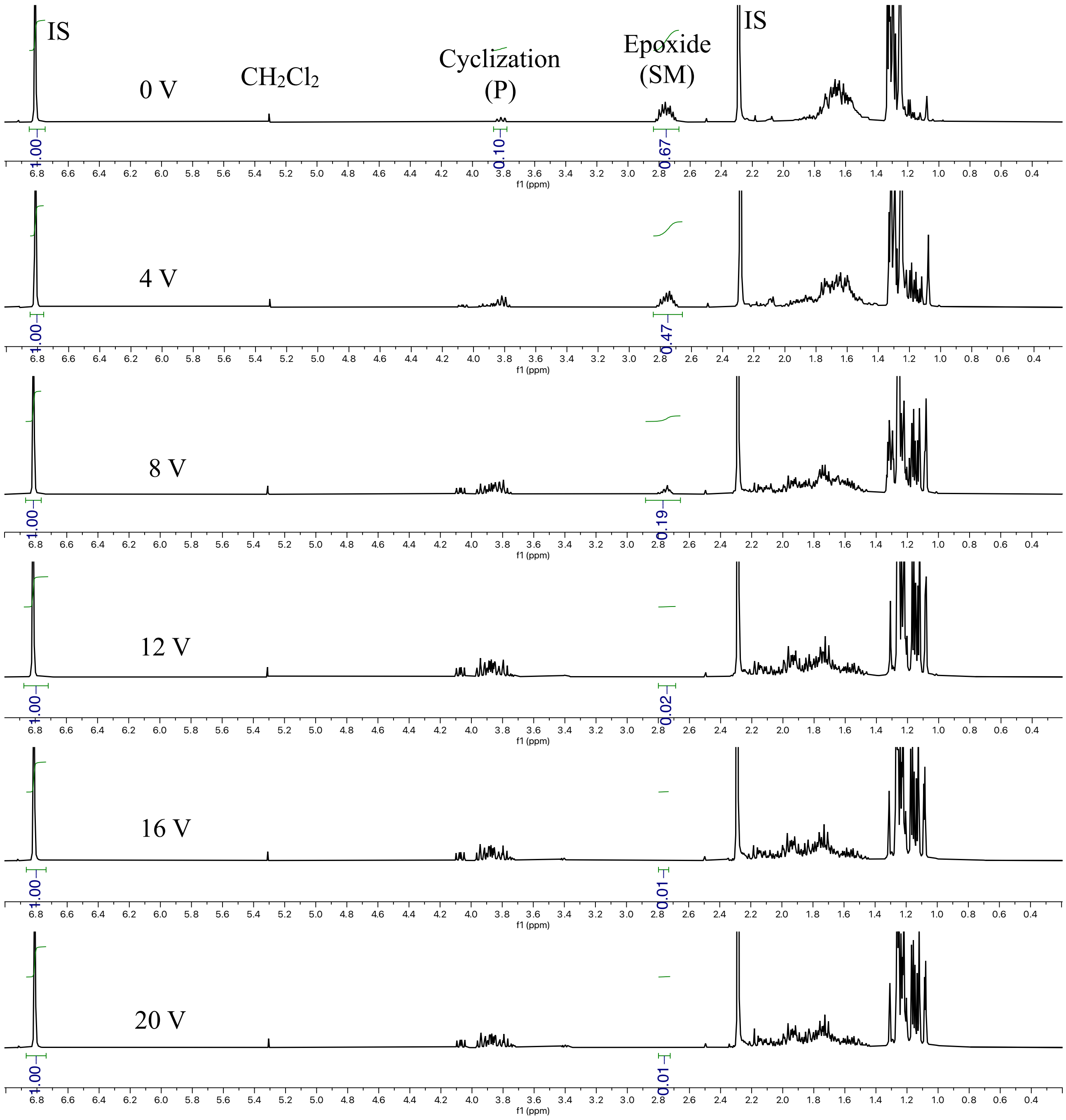

Figure S17. ^{1}H NMR spectra of the cyclization reaction of **2** under EFC with anionic acid **1** and thiourea **11** in $CDCl_3$ at different voltages.

Table S7. EFC with substrate **2**, anionic acid **1** and thiourea **11**.[a]

Entry	S (mM)[b]	C (mol%)[c]	B (mol%)[d]	V (V)[e]	I (A)[f]	Y_m (%)[g]
1	153	0.25	0.25	0	0.000	0
2	153	0.25	0.25	4	0.000	30
3	153	0.25	0.25	8	0.000	71
4	153	0.25	0.25	12	0.000	98
5	153	0.25	0.25	16	0.000	99
6	153	0.25	0.25	20	0.000	99

[a]In microfluidic capacitor in $CDCl_3$, rt, flowrate 30 µL/min. [b]Concentration of substrate **2**, in millimolar. [c]Concentration of catalyst **1**, in mol% of substrate. [d]Concentration of ion-pair breaker, in mol% of substrate. [e]Applied voltage, in volts. [f]Current measured, in amperes. [g]Microfluidic yield of total cyclization products, in percent.

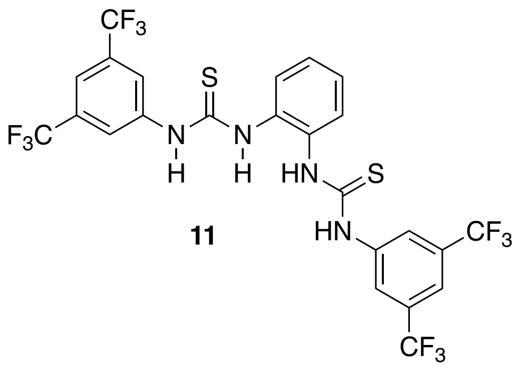

11

Y_m^{max} = 99% V_c = 2.5 V

n_g = 2.8

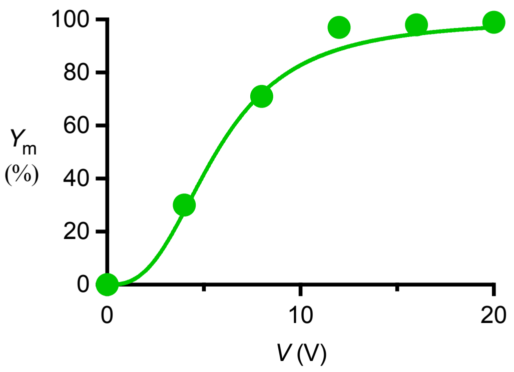

Figure S18. Yield (Y_m) of **3** under EFC with anionic acid **1** and thiourea **11** in $CDCl_3$ as a function of the voltage. With Y_m^{max} as maximum microfluidic yield, V_c as critical voltage and n_g as gating coefficient.

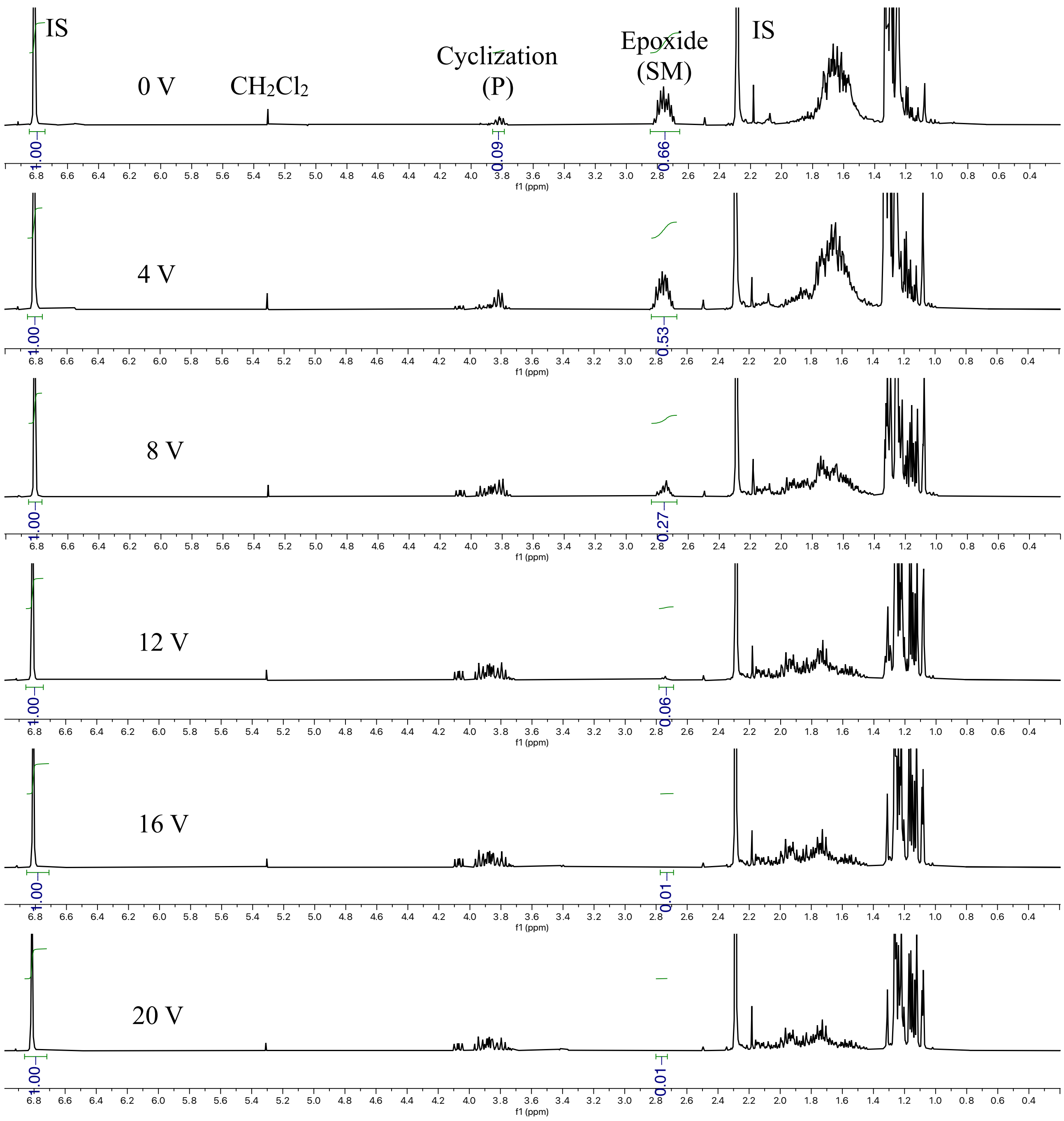

Figure S19. ^{1}H NMR spectra of the cyclization reaction of **2** under EFC with anionic acid **1** and thiourea **12** in $CDCl_3$ at different voltages.

Table S8. EFC with substrate **2**, anionic acid **1** and with thiourea **12**.[a]

Entry	S (mM)[b]	C (mol%)[c]	B (mol%)[d]	V (V)[e]	I (A)[f]	Y_m (%)[g]
1	153	0.25	0.25	0	0.000	0
2	153	0.25	0.25	4	0.000	20
3	153	0.25	0.25	8	0.000	60
4	153	0.25	0.25	12	0.000	91
5	153	0.25	0.25	16	0.000	98
6	153	0.25	0.25	20	0.000	98

[a]In microfluidic capacitor in $CDCl_3$, rt, flowrate 30 μL/min. [b]Concentration of substrate **2**, in millimolar. [c]Concentration of catalyst **1**, in mol% of substrate. [d]Concentration of ion-pair breaker, in mol% of substrate. [e]Applied voltage, in volts. [f]Current measured, in amperes. [g]Microfluidic yield of total cyclization products, in percent.

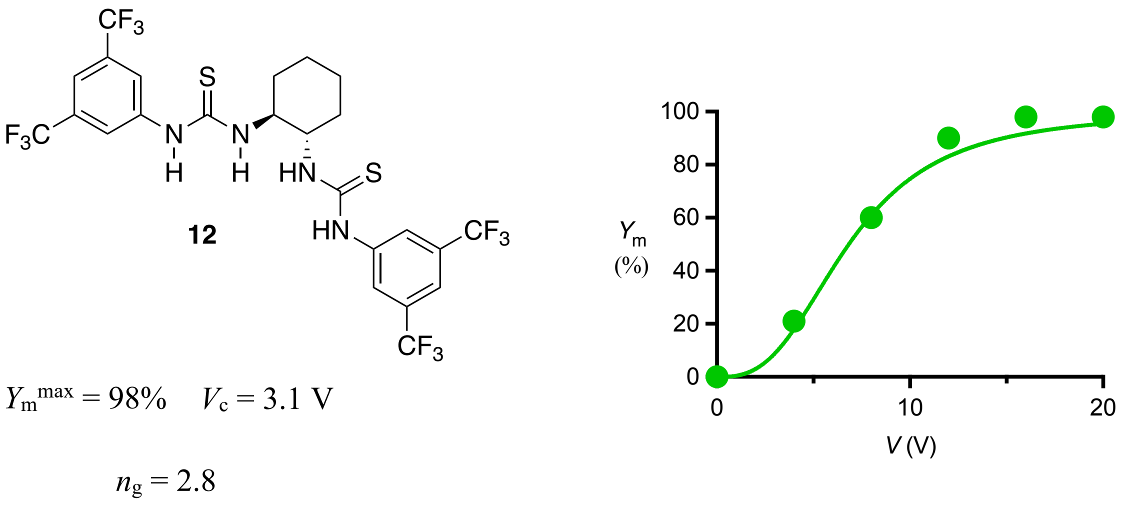

Figure S20. Yield (Y_m) of **3** under EFC with anionic acid **1** and thiourea **12** in $CDCl_3$ as a function of the voltage. With Y_m^{max} as maximum microfluidic yield, V_c as critical voltage and n_g as gating coefficient.

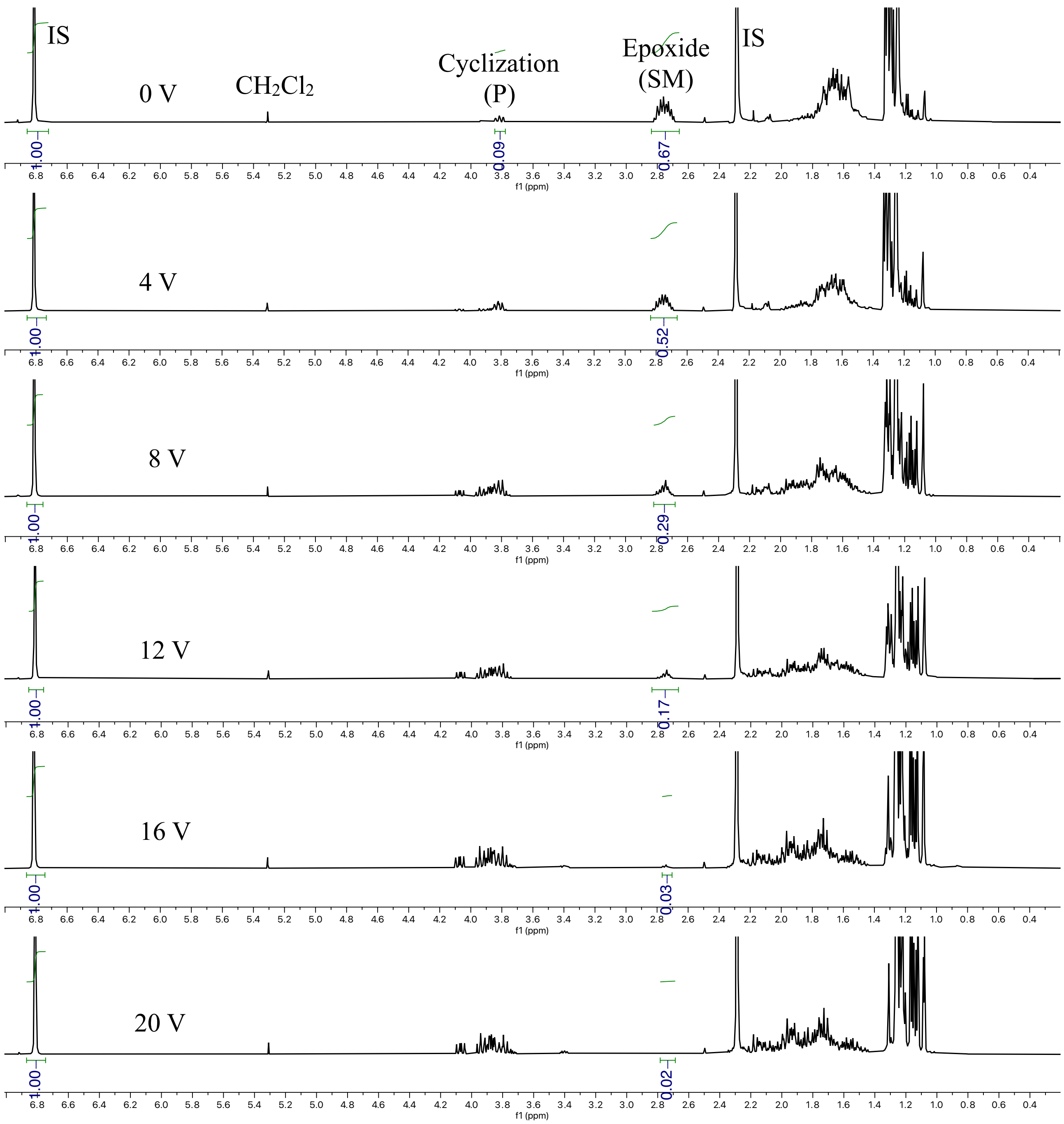

Figure S21. ^{1}H NMR spectra of the cyclization reaction of **2** under EFC with anionic acid **1** and thiourea **13** in $CDCl_3$ at different voltages.

Table S9. EFC with substrate **2**, anionic acid **1** and thiourea **13**.[a]

Entry	S (mM)[b]	C (mol%)[c]	B (mol%)[d]	V (V)[e]	I (A)[f]	Y_m (%)[g]
1	153	0.25	0.25	0	0.000	0
2	153	0.25	0.25	4	0.000	23
3	153	0.25	0.25	8	0.000	57
4	153	0.25	0.25	12	0.000	75
5	153	0.25	0.25	16	0.000	96
6	153	0.25	0.25	20	0.000	97

[a]In microfluidic capacitor in $CDCl_3$, rt, flowrate 30 µL/min. [b]Concentration of substrate **2**, in millimolar. [c]Concentration of catalyst **1**, in mol% of substrate. [d]Concentration of ion-pair breaker, in mol% of substrate. [e]Applied voltage, in volts. [f]Current measured, in amperes. [g]Microfluidic yield of total cyclization products, in percent.

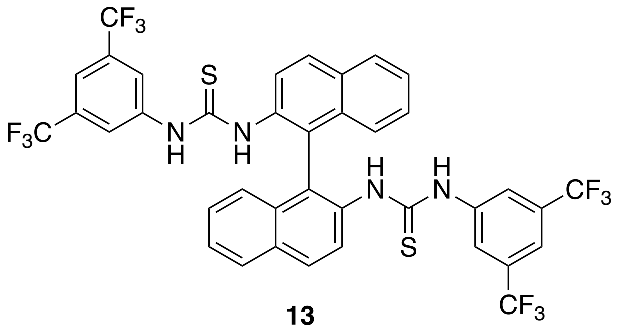

Y_m^{max} = 97% V_c = 2.7 V

n_g = 2.4

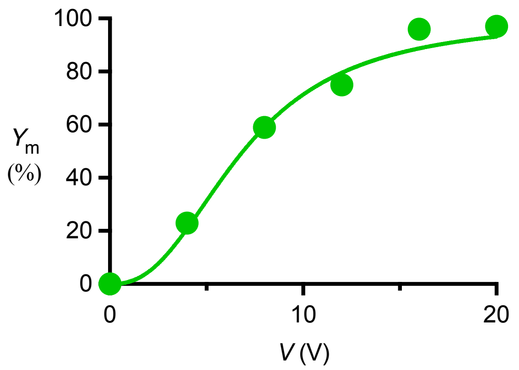

Figure S22. Yield (Y_m) of **3** under EFC with anionic acid **1** and thiourea **13** in $CDCl_3$ as a function of the voltage. With Y_m^{max} as maximum microfluidic yield, V_c as critical voltage and n_g as gating coefficient.

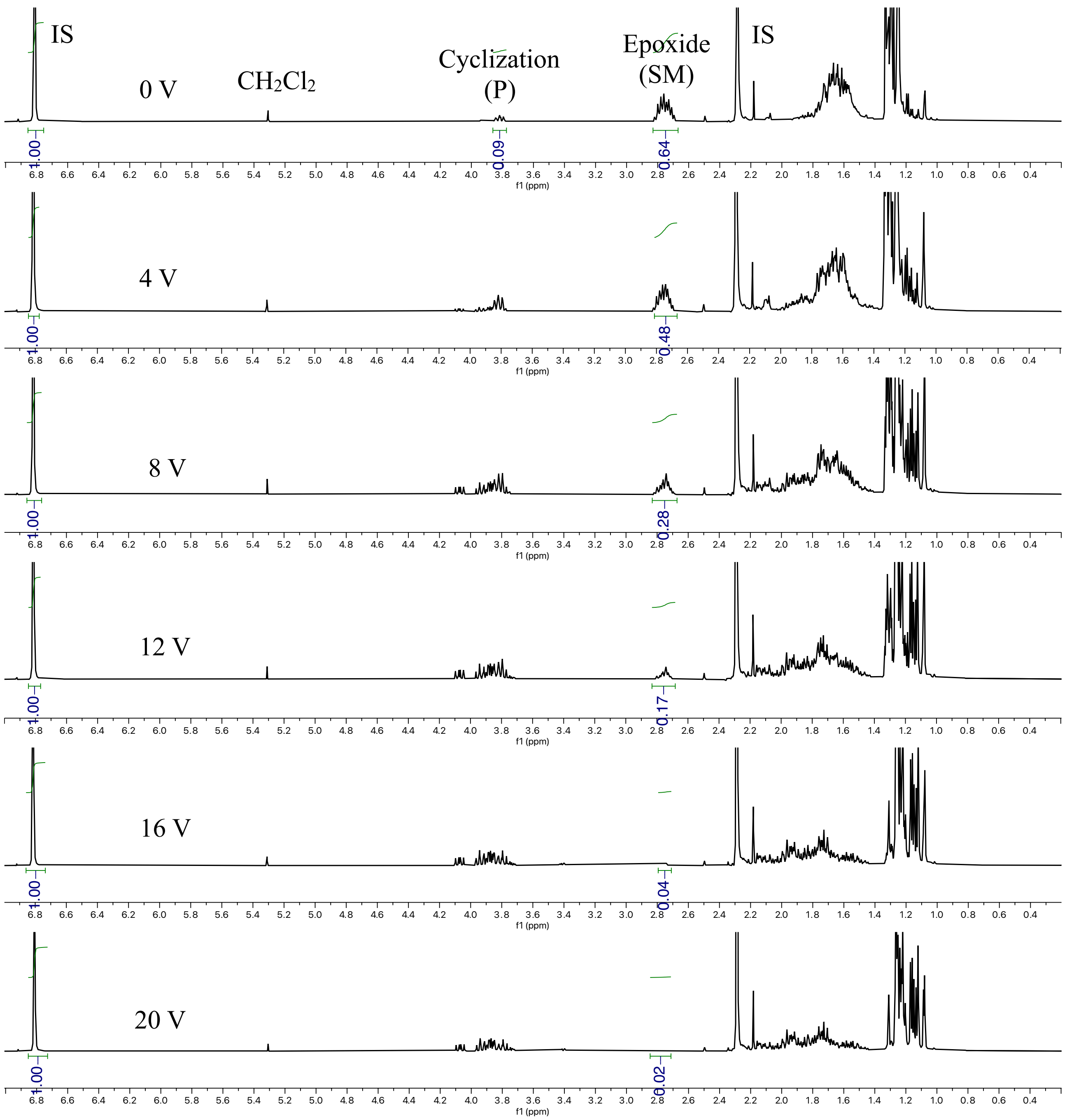

Figure S23. ^{1}H NMR spectra of the cyclization reaction of **2** under EFC with anionic acid **1** and thiourea **14** in $CDCl_3$ at different voltages.

Table S10. EFC with substrate **2**, anionic acid **1** and with thiourea **14**.[a]

Entry	S (mM)[b]	C (mol%)[c]	B (mol%)[d]	V (V)[e]	I (A)[f]	Y_m (%)[g]
1	153	0.25	0.25	0	0.000	0
2	153	0.25	0.25	4	0.000	25
3	153	0.25	0.25	8	0.000	57
4	153	0.25	0.25	12	0.000	74
5	153	0.25	0.25	16	0.000	94
6	153	0.25	0.25	20	0.000	97

[a]In microfluidic capacitor in $CDCl_3$, rt, flowrate 30 μL/min. [b]Concentration of substrate **2**, in millimolar. [c]Concentration of catalyst **1**, in mol% of substrate. [d]Concentration of ion-pair breaker, in mol% of substrate. [e]Applied voltage, in volts. [f]Current measured, in amperes. [g]Microfluidic yield of total cyclization products, in percent.

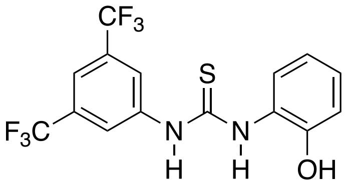

14

$Y_m^{max} = 97\%$ $V_c = 2.6$ V

$n_g = 2.3$

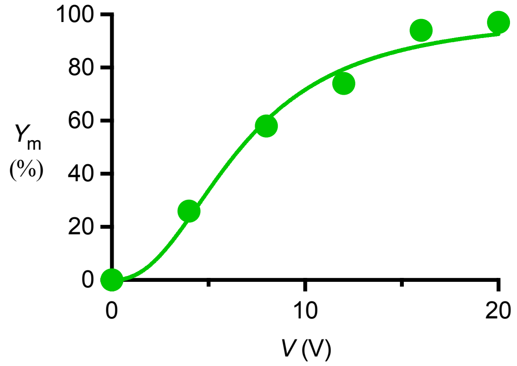

Figure S24. Yield (Y_m) of **3** under EFC with anionic acid **1** and thiourea **14** in $CDCl_3$ as a function of the voltage. With Y_m^{max} as maximum microfluidic yield, V_c as critical voltage and n_g as gating coefficient.

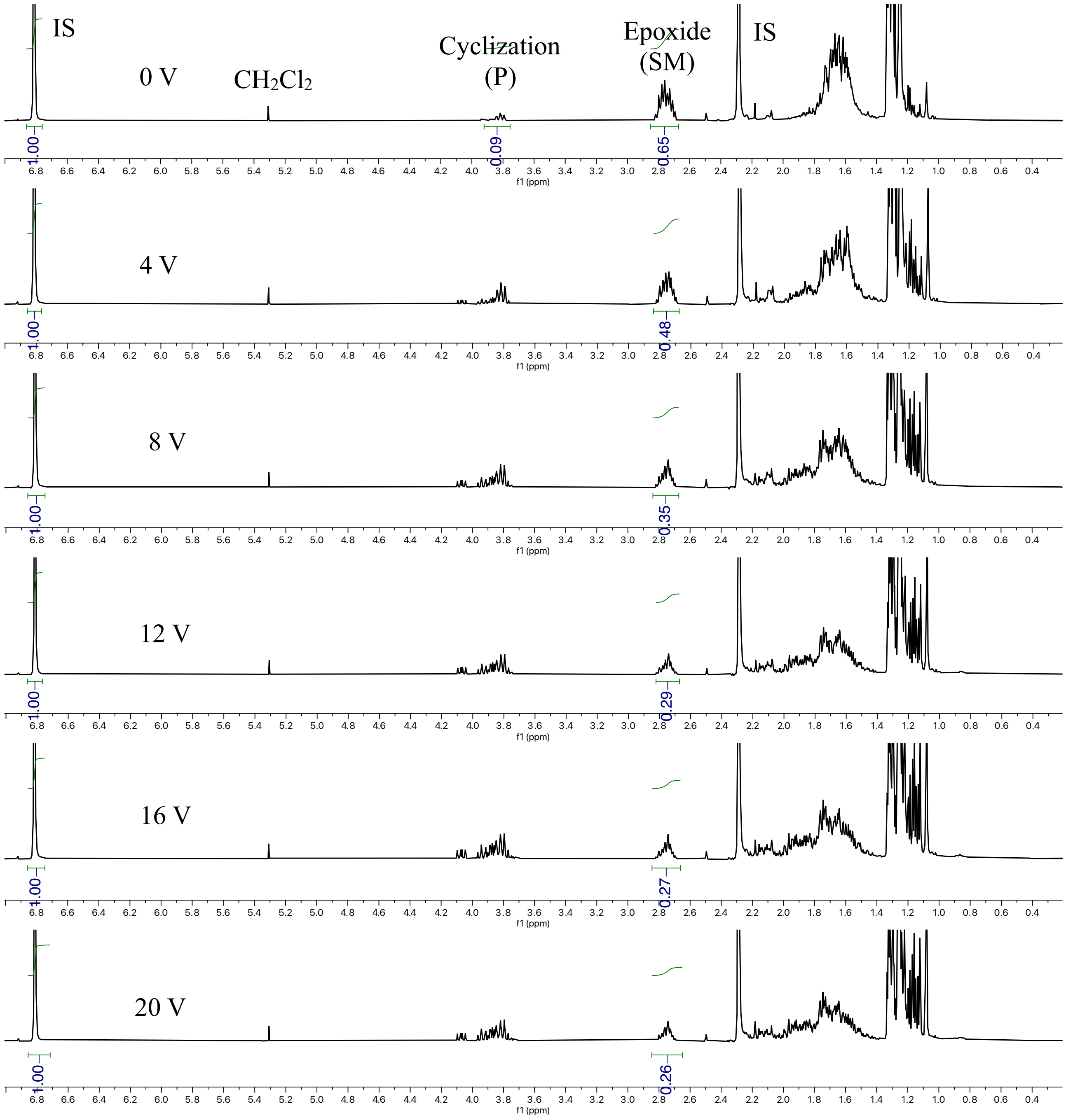

Figure S25. ^{1}H NMR spectra of the cyclization reaction of **2** under EFC with anionic acid **1** and catechol **15** in $CDCl_3$ at different voltages.

Table S11. EFC with substrate **2**, anionic acid **1** and with thiourea **15**.[a]

Entry	S (mM)[b]	C (mol%)[c]	B (mol%)[d]	V (V)[e]	I (A)[f]	Y_m (%)[g]
1	153	0.25	0.25	0	0.000	0
2	153	0.25	0.25	4	0.000	26
3	153	0.25	0.25	8	0.000	46
4	153	0.25	0.25	12	0.000	55
5	153	0.25	0.25	16	0.000	58
6	153	0.25	0.25	20	0.000	60

[a]In microfluidic capacitor in $CDCl_3$, rt, flowrate 30 μL/min. [b]Concentration of substrate **2**, in millimolar. [c]Concentration of catalyst **1**, in mol% of substrate. [d]Concentration of ion-pair breaker, in mol% of substrate. [e]Applied voltage, in volts. [f]Current measured, in amperes. [g]Microfluidic yield of total cyclization products, in percent.

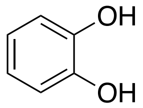

15

Y_m^{max} = 60% V_c = 2.0 V

n_g = 1.9

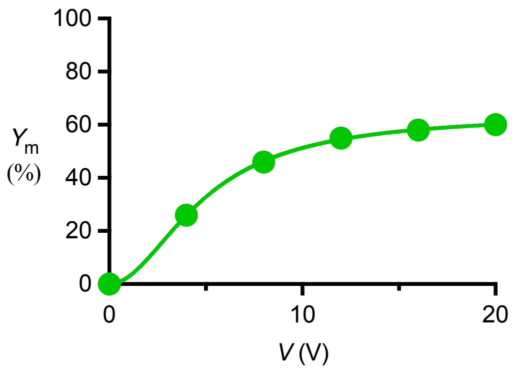

Figure S26. Yield (Y_m) of **3** under EFC with anionic acid **1** and catechol **15** in $CDCl_3$ as a function of the voltage. With Y_m^{max} as maximum microfluidic yield, V_c as critical voltage and n_g as gating coefficient.

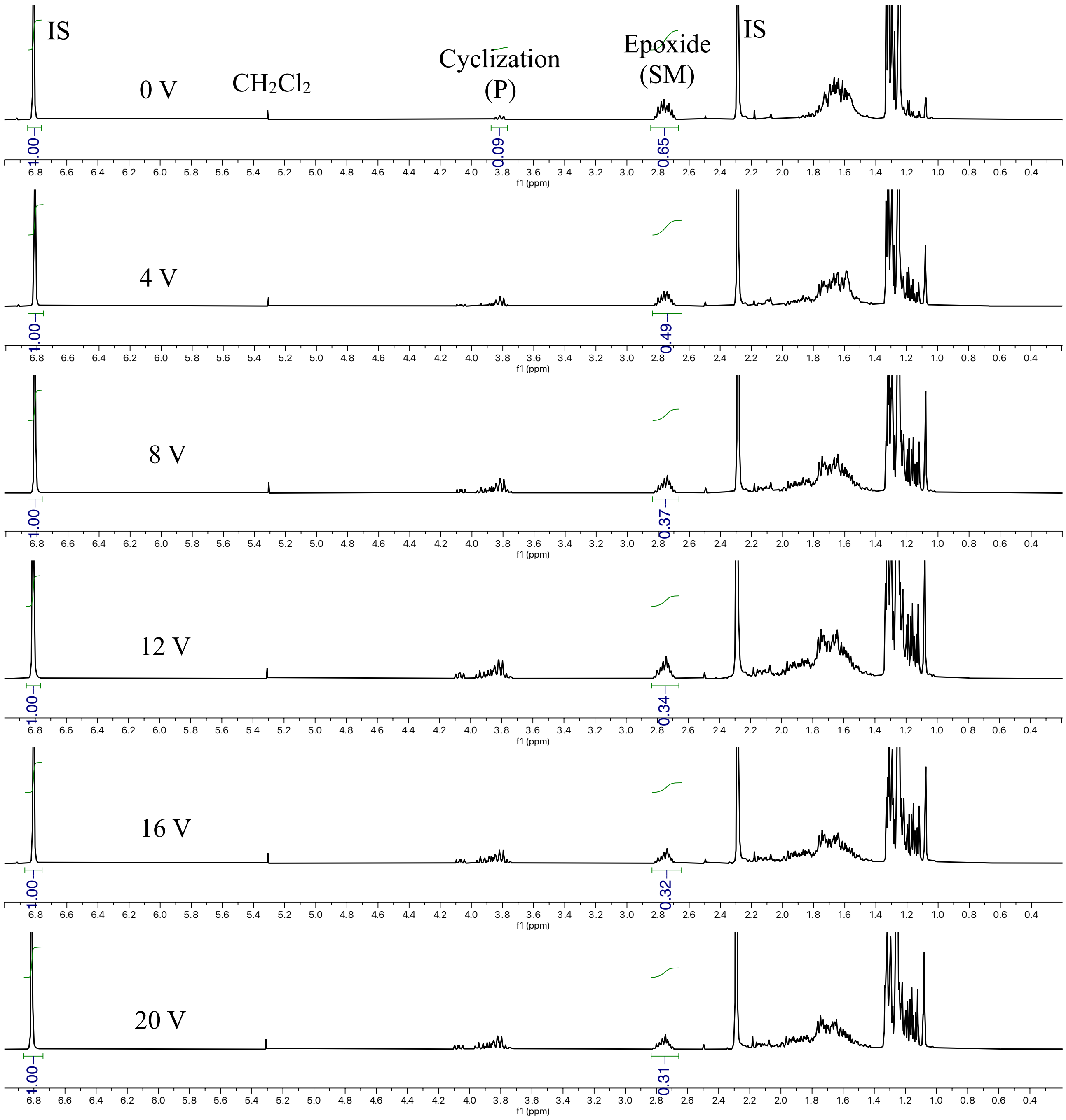

Figure S27. ^{1}H NMR spectra of the cyclization reaction of **2** under EFC with anionic acid **1** and catechol **16** in $CDCl_3$ at different voltages.

Table S12. EFC with substrate **2**, anionic acid **1** and with thiourea **16**.[a]

Entry	S (mM)[b]	C (mol%)[c]	B (mol%)[d]	V (V)[e]	I (A)[f]	Y_m (%)[g]
1	153	0.25	0.25	0	0.000	0
2	153	0.25	0.25	4	0.000	25
3	153	0.25	0.25	8	0.000	43
4	153	0.25	0.25	12	0.000	48
5	153	0.25	0.25	16	0.000	51
6	153	0.25	0.25	20	0.000	52

[a]In microfluidic capacitor in $CDCl_3$, rt, flowrate 30 µL/min. [b]Concentration of substrate **2**, in millimolar. [c]Concentration of catalyst **1**, in mol% of substrate. [d]Concentration of ion-pair breaker, in mol% of substrate. [e]Applied voltage, in volts. [f]Current measured, in amperes. [g]Microfluidic yield of total cyclization products, in percent.

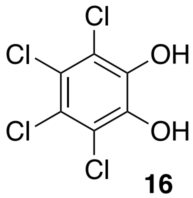

16

Y_m^{max} = 52% V_c = 2.2 V

n_g = 2.3

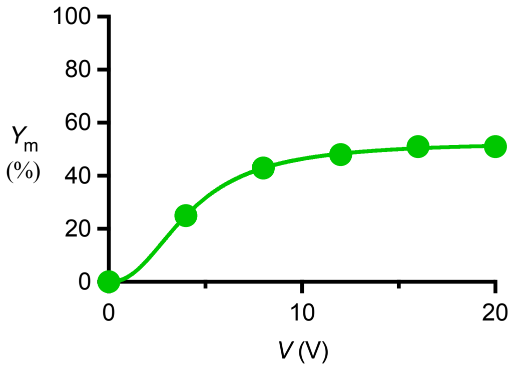

Figure S28. Yield (Y_m) of **3** under EFC with anionic acid **1** and catechol **16** in $CDCl_3$ as a function of the voltage. With Y_m^{max} as maximum microfluidic yield, V_c as critical voltage and n_g as gating coefficient.

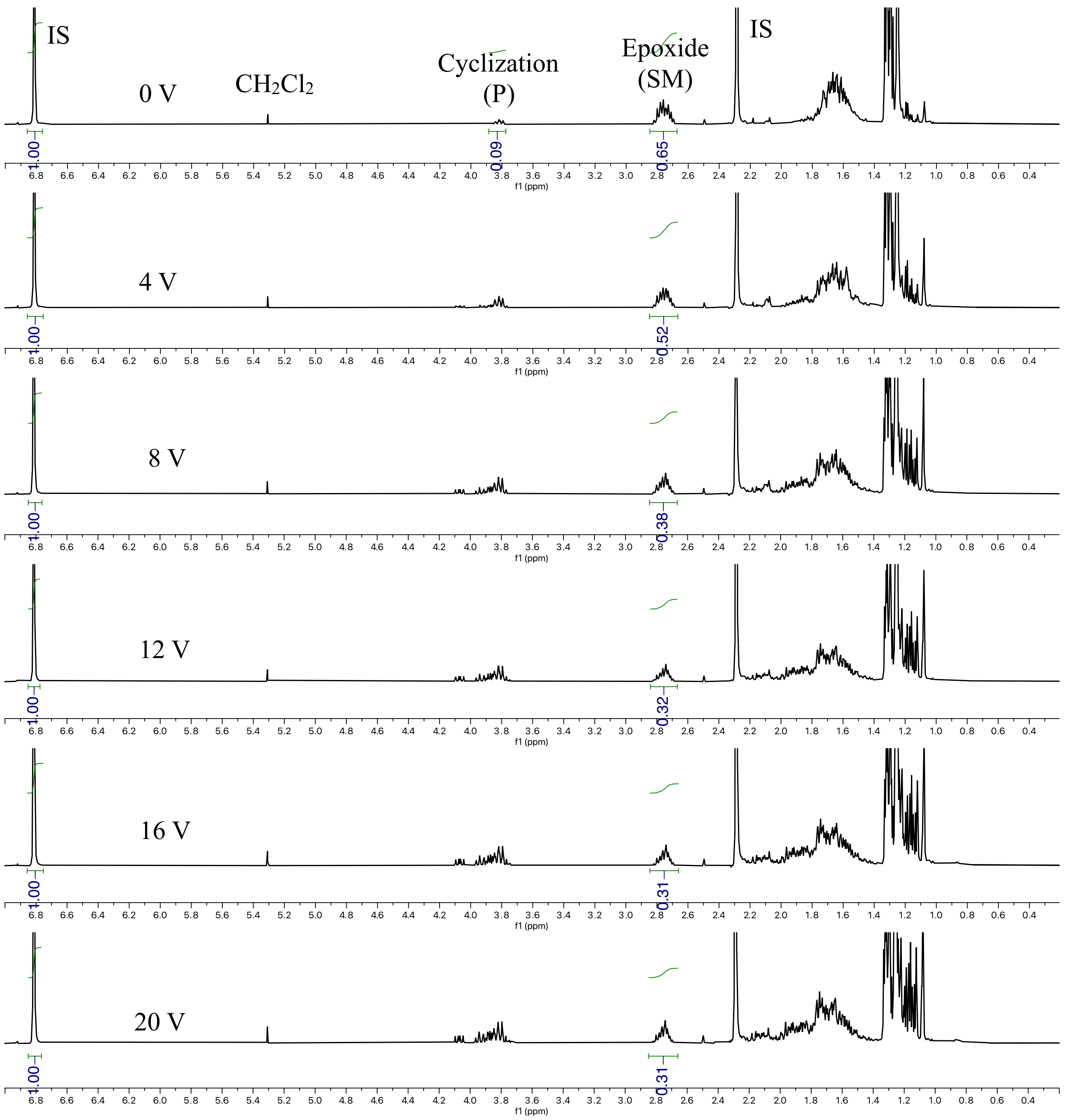

Figure S29. ^{1}H NMR spectra of the cyclization reaction of **2** under EFC with anionic acid **1** and catechol **17** in $CDCl_3$ at different voltages.

Table S13. EFC with substrate **2**, anionic acid **1** and thiourea **17**.[a]

Entry	S (mM)[b]	C (mol%)[c]	B (mol%)[d]	V (V)[e]	I (A)[f]	Y_m (%)[g]
1	153	0.25	0.25	0	0.000	0
2	153	0.25	0.25	4	0.000	20
3	153	0.25	0.25	8	0.000	42
4	153	0.25	0.25	12	0.000	51
5	153	0.25	0.25	16	0.000	53
6	153	0.25	0.25	20	0.000	53

[a]In microfluidic capacitor in $CDCl_3$, rt, flowrate 30 µL/min. [b]Concentration of substrate **2**, in millimolar. [c]Concentration of catalyst **1**, in mol% of substrate. [d]Concentration of ion-pair breaker, in mol% of substrate. [e]Applied voltage, in volts. [f]Current measured, in amperes. [g]Microfluidic yield of total cyclization products, in percent.

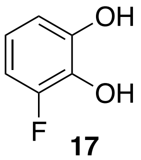

17

Y_m^{max} = 53% V_c = 2.8 V

n_g = 2.6

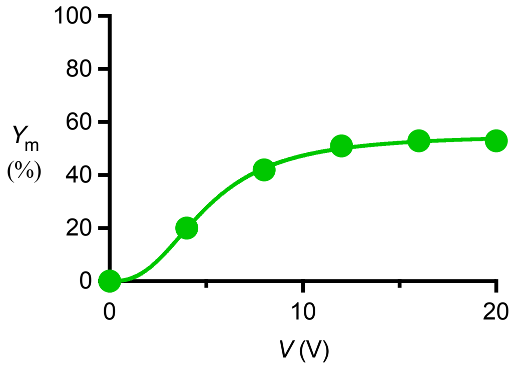

Figure S30. Yield (Y_m) of **3** under EFC with anionic acid **1** and catechol **17** in $CDCl_3$ as a function of the voltage. With Y_m^{max} as maximum microfluidic yield, V_c as critical voltage and n_g as gating coefficient.

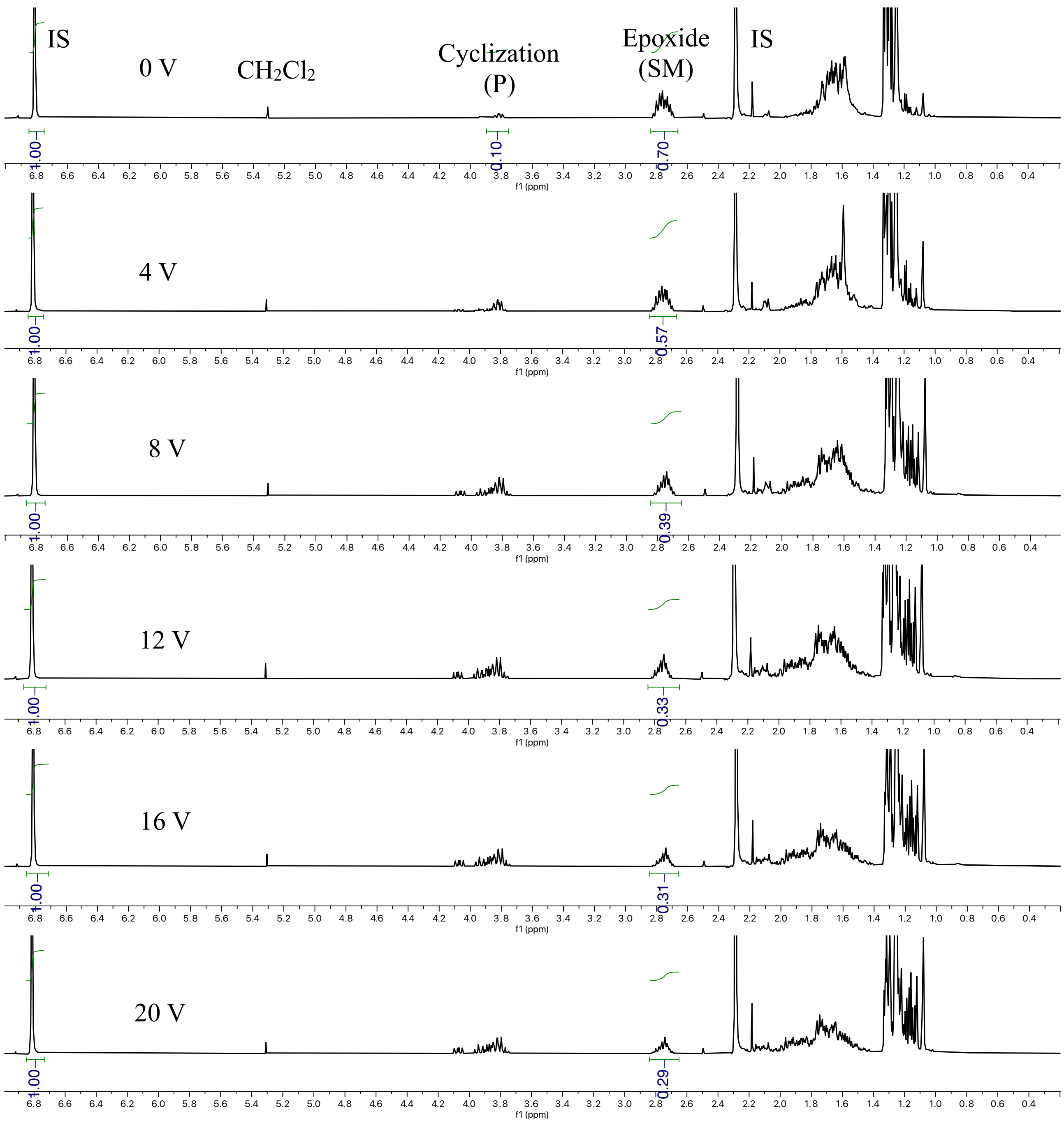

Figure S31. ^{1}H NMR spectra of the cyclization reaction of **2** under EFC with anionic acid **1** and catechol **18** in $CDCl_3$ at different voltages.

Table S14. EFC with substrate **2**, anionic acid **1** and thiourea **18**.[a]

Entry	S (mM)[b]	C (mol%)[c]	B (mol%)[d]	V (V)[e]	I (A)[f]	Y_m (%)[g]
1	153	0.25	0.25	0	0.000	0
2	153	0.25	0.25	4	0.000	19
3	153	0.25	0.25	8	0.000	44
4	153	0.25	0.25	12	0.000	53
5	153	0.25	0.25	16	0.000	56
6	153	0.25	0.25	20	0.000	59

[a]In microfluidic capacitor in $CDCl_3$, rt, flowrate 30 μL/min. [b]Concentration of substrate **2**, in millimolar. [c]Concentration of catalyst **1**, in mol% of substrate. [d]Concentration of ion-pair breaker, in mol% of substrate. [e]Applied voltage, in volts. [f]Current measured, in amperes. [g]Microfluidic yield of total cyclization products, in percent.

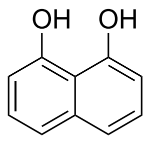

18

Y_m^{max} = 59% V_c = 2.8 V

n_g = 2.5

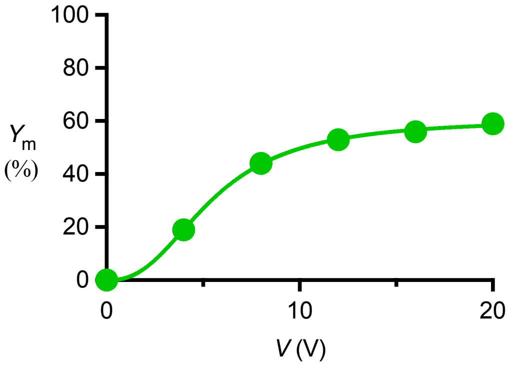

Figure S32. Yield (Y_m) of **3** under EFC with anionic acid **1** and catechol **18** in $CDCl_3$ as a function of the voltage. With Y_m^{max} as maximum microfluidic yield, V_c as critical voltage and n_g as gating coefficient.

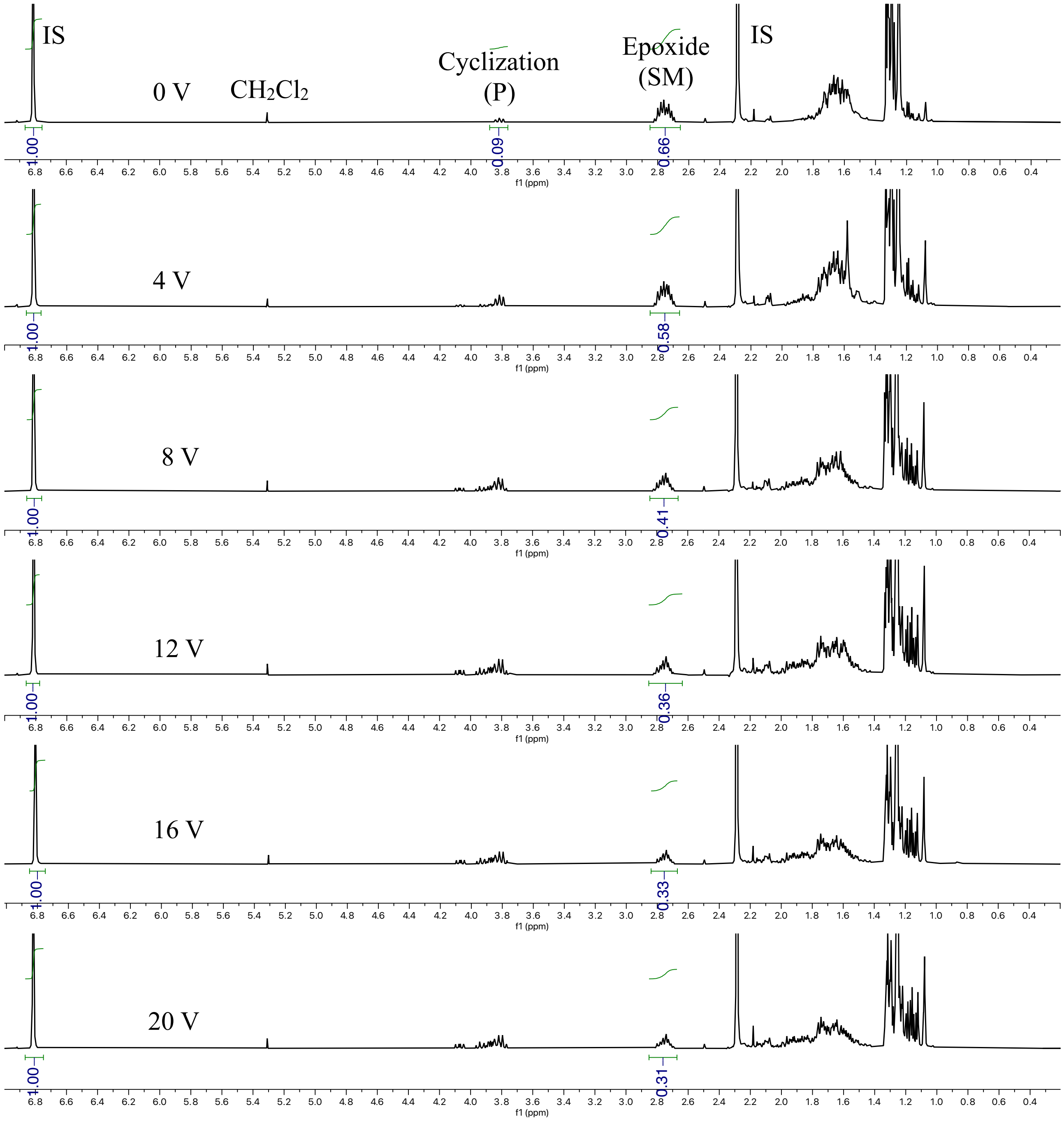

Figure S33. ^{1}H NMR spectra of the cyclization reaction of **2** under EFC with anionic acid **1** and catechol **19** in $CDCl_3$ at different voltages.

Table S15. EFC with substrate **2**, anionic acid **1** and thiourea **19**.[a]

Entry	S (mM)[b]	C (mol%)[c]	B (mol%)[d]	V (V)[e]	I (A)[f]	Y_m (%)[g]
1	153	0.25	0.25	0	0.000	0
2	153	0.25	0.25	4	0.000	12
3	153	0.25	0.25	8	0.000	38
4	153	0.25	0.25	12	0.000	45
5	153	0.25	0.25	16	0.000	50
6	153	0.25	0.25	20	0.000	53

[a]In microfluidic capacitor in $CDCl_3$, rt, flowrate 30 µL/min. [b]Concentration of substrate **2**, in millimolar. [c]Concentration of catalyst **1**, in mol% of substrate. [d]Concentration of ion-pair breaker, in mol% of substrate. [e]Applied voltage, in volts. [f]Current measured, in amperes. [g]Microfluidic yield of total cyclization products, in percent.

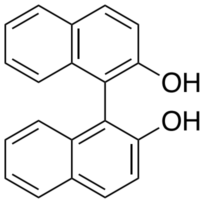

19

$Y_m^{max} = 53\%$ $V_c = 3.6$ V

$n_g = 2.9$

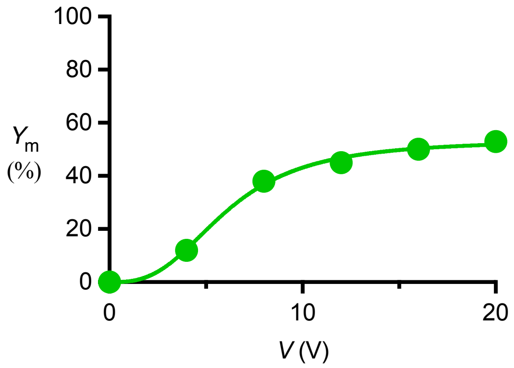

Figure S34. Yield (Y_m) of **3** under EFC with anionic acid **1** and catechol **19** in $CDCl_3$ as a function of the voltage. With Y_m^{max} as maximum microfluidic yield, V_c as critical voltage and n_g as gating coefficient.

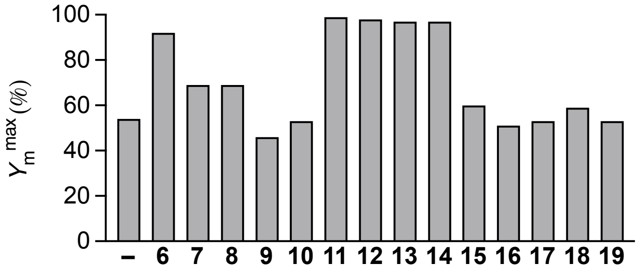

Figure S35. Maximum yields (Y_m^{max}) at 20 V with anionic acid **1**, either without (**-**) or with ion-pair breakers (**6-19**).

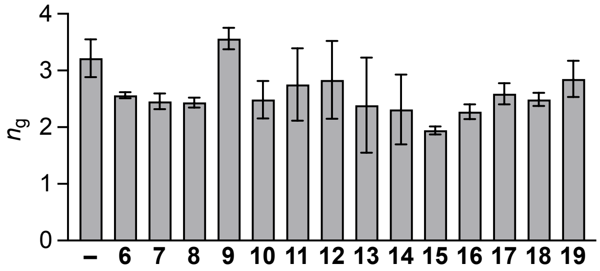

Figure S36. Gating coefficients (n_g) with anionic acid **1**, either without (**-**) or with ion-pair breakers (**6-19**). Error bars represent SEM of the curve fit.

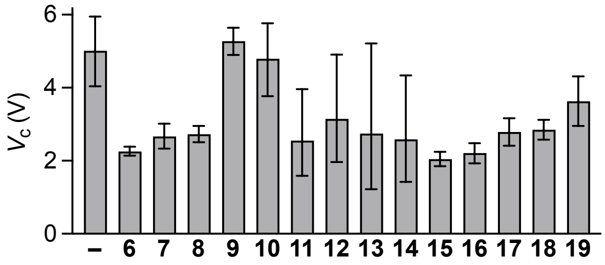

Figure S37. Critical voltage (V_c) with anionic acid **1**, either without (**-**) or with ion-pair breakers (**6-19**). Error bars represent the confidence intervals (95%) of the curve fit.

3.5. Control Experiments

3.5.1. Ion-Pair Breakers without Anionic Brønsted Acids

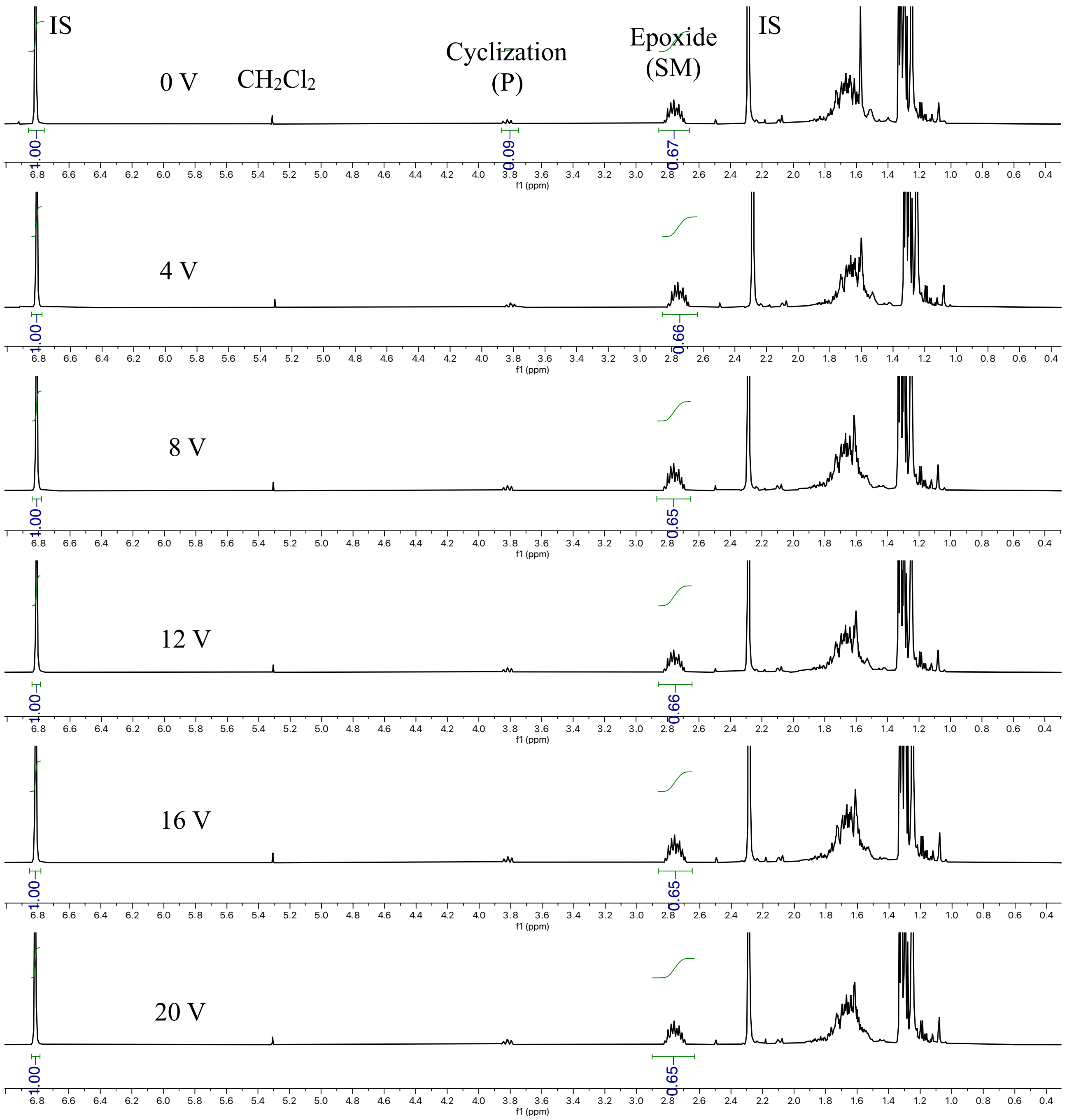

Figure S38. ^{1}H NMR spectra of the cyclization reaction of **2** under EFC without anionic acid **1** and with thiourea **6** in $CDCl_3$ at different voltages.

Table S16. EFC with substrate **2**, without anionic acid **1** and with thiourea **6**.[a]

Entry	S (mM)[b]	C (mol%)[c]	B (mol%)[d]	V (V)[e]	I (A)[f]	Y_m (%)[g]
1	153	0	0.25	0	0.000	0
2	153	0	0.25	4	0.000	1
3	153	0	0.25	8	0.000	3
4	153	0	0.25	12	0.000	1
5	153	0	0.25	16	0.000	3
6	153	0.	0.25	20	0.000	3

[a]In microfluidic capacitor in $CDCl_3$, rt, flowrate 30 µL/min. [b]Concentration of substrate **2**, in millimolar. [c]Concentration of catalyst **1**, in mol% of substrate. [d]Concentration of ion-pair breaker, in mol% of substrate. [e]Applied voltage, in volts. [f]Current measured, in amperes. [g]Microfluidic yield of total cyclization products, in percent.

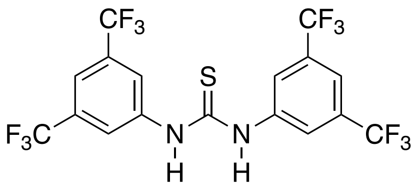

6

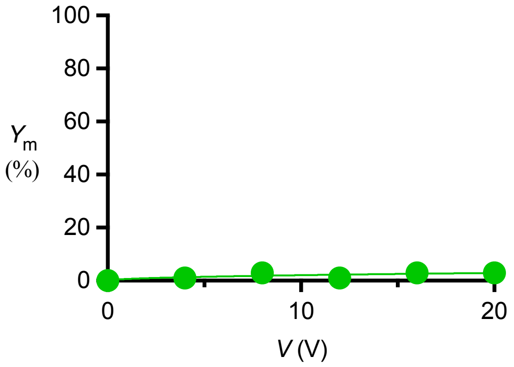

Figure S39. Yield (Y_m) of **3** under EFC with thiourea **6** in $CDCl_3$ as a function of the voltage.

3.5.2. Non-Acidic Anions in Place of Anionic Brønsted Acids

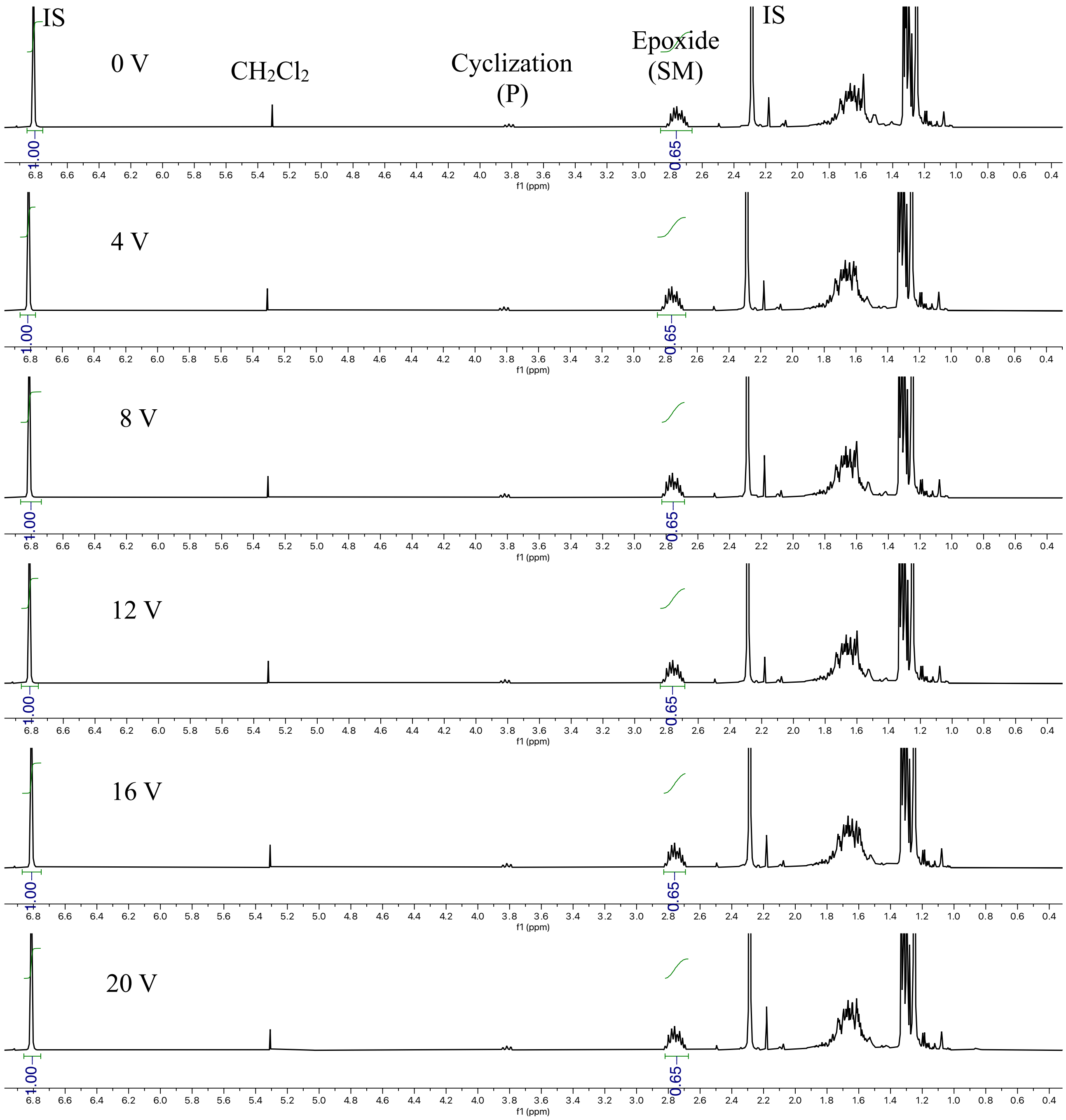

Figure S40. ^{1}H NMR spectra of the cyclization reaction of **2** under EFC without anionic acid **1** and with non-acidic TBACl **5** in $CDCl_3$ at different voltages.

Table S17. EFC with substrate **2**, with non-acidic TBACl **5**.[a]

Entry	S (mM)[b]	C (mol%)[c]	B (mol%)[d]	*V* (V)[e]	*I* (A)[f]	Y_m (%)[g]
1	153	0.25	0	0	0.000	0
2	153	0.25	0	4	0.000	0
3	153	0.25	0	8	0.000	0
4	153	0.25	0	12	0.000	0
5	153	0.25	0	16	0.000	0
6	153	0.25	0	20	0.000	0

[a]In microfluidic capacitor in $CDCl_3$, rt, flowrate 30 µL/min. [b]Concentration of substrate **2**, in millimolar. [c]Concentration of “catalyst” **5**, in mol% of substrate. [d]Concentration of ion-pair breaker, in mol% of substrate. [e]Applied voltage, in volts. [f]Current measured, in amperes. [g]Microfluidic yield of total cyclization products, in percent.

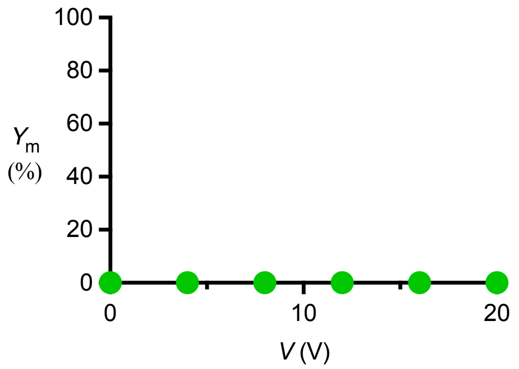

Figure S41. Yield (Y_m) of **3** under EFC with non-acidic TBACl **5** in $CDCl_3$ as a function of the voltage.

3.5.3. Water Sensitivity

Without ion-pair breakers at 12 V. A 1:1 biphasic mixture of $CDCl_3$ and D_2O was stirred for 5 min. The organic phase was taken, and a solution with substrate **2** (153 mM), mesitylene as internal standard (153 mM) and anionic acid **1** (0.25 mol%) was prepared and charged in a single syringe. The solution was infused at 30 μL/min into the microfluidic capacitor under constant voltage. One and a half reactor volumes (0.45 mL) were discarded before collecting each sample to ensure that a steady state of the system had been reached at the desired voltage. Samples of 150 μL were collected during a period of 5 min. Samples were diluted with additional $CDCl_3$, and ^1H NMR spectra were acquired immediately after each experiment. Yield was determined by comparing the integral of the epoxide signal to that of the internal standard. All experiments were conducted under I = 0.000 A.

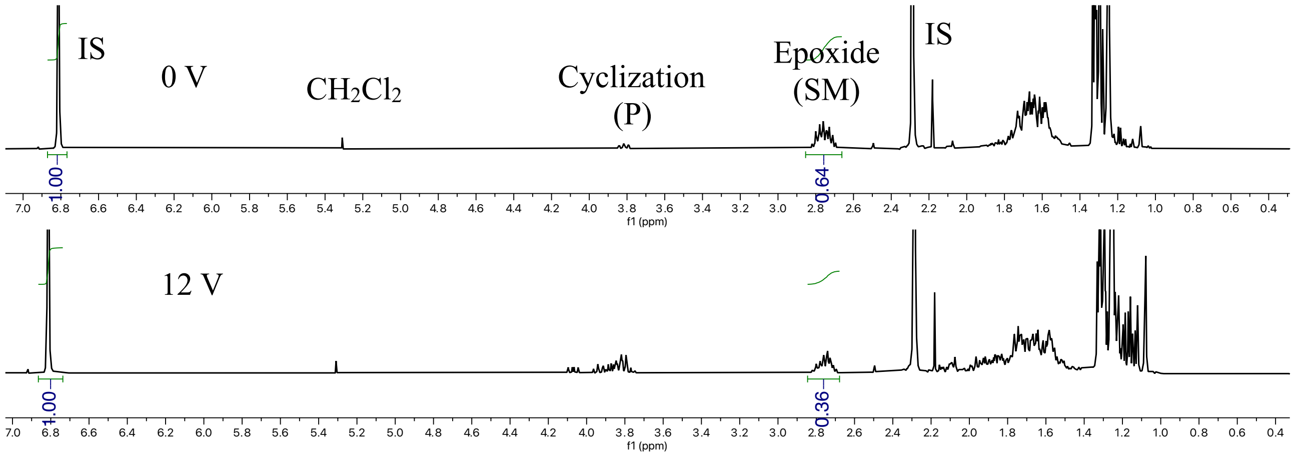

Figure S42. ^1H NMR spectra of the cyclization reaction mixture of **2** under EFC with anionic acid **1** in $CDCl_3$ at 0 and 12 V.

With ion-pair breakers at 12 V. A 1:1 biphasic mixture of $CDCl_3$ and D_2O was stirred for 5 min. The organic phase was taken, and a solution with substrate **2** (153 mM), mesitylene as internal standard (153 mM), anionic acid **1** (0.25 mol%) and thiourea **6** (0.25 mol%) was prepared and charged in a single syringe. The solution was infused at 30 µL/min into the microfluidic capacitor under constant voltage. One and a half reactor volumes (0.45 mL) were discarded before collecting each sample to ensure that a steady state of the system had been reached at the desired voltage. Samples of 150 µL were collected during a period of 5 min. Samples were diluted with additional $CDCl_3$, and ^1H NMR spectra were acquired immediately after each experiment. Yield was determined by comparing the integral of the epoxide signal to that of the internal standard. All experiments were conducted under I = 0.000 A.

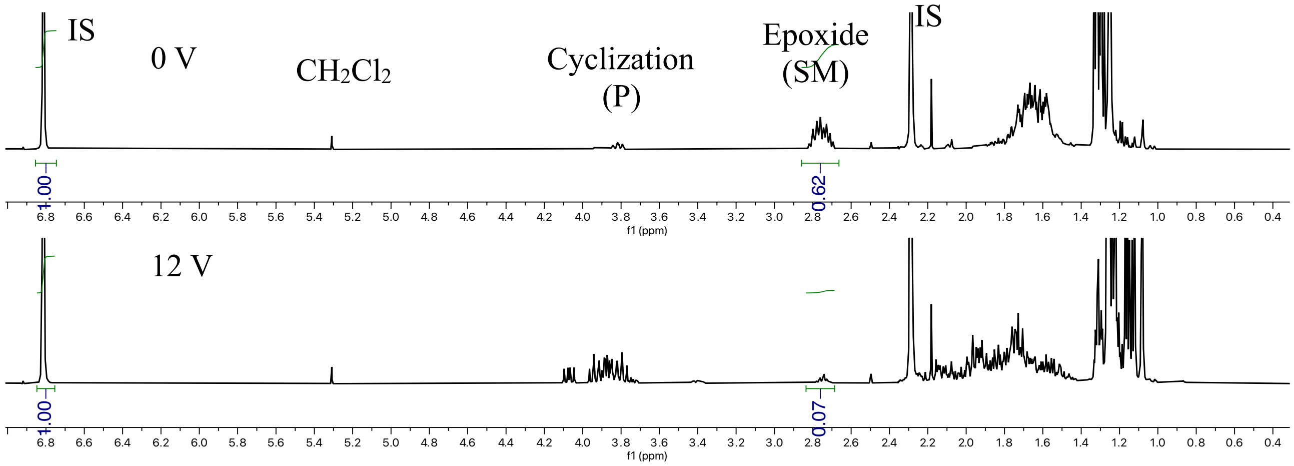

Figure S43. ^1H NMR spectra of the cyclization reaction mixture of **2** under EFC with anionic acid **1** and thiourea **6** in $CDCl_3$ at 0 and 12 V.

3.5.4. Radical Scavengers

A solution of substrate **2** (153 mM), 1,1-diphenylethylene **4** (153 mM), mesitylene as internal standard (153 mM) and anionic acid **1** (0.25 mol%) was prepared in $CDCl_3$ and charged in a single syringe. The solution was infused at 30 μL/min into the microfluidic capacitor under constant voltage. One and a half reactor volumes (0.45 mL) were discarded before collecting each sample to ensure that a steady state of the system had been reached at the desired voltage. Samples of 150 μL were collected during a period of 5 min. Samples were diluted with additional $CDCl_3$, and ^{1}H NMR spectra were acquired immediately after each experiment. Yield was determined by comparing the integral of the epoxide signal to that of the internal standard. All experiments were conducted under $I = 0.000$ A.

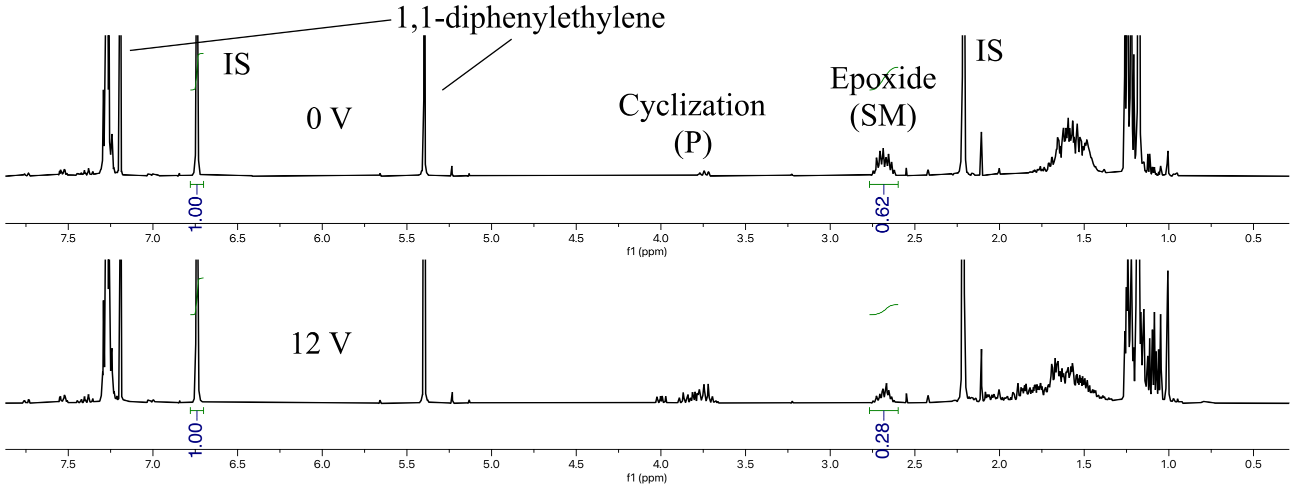

Figure S44. ^{1}H NMR spectra of the cyclization reaction mixture of **2** under EFC with anionic acid **1** and radical scavenger **4** in $CDCl_3$ at 0 and 12 V.

Table S18. EFC with diepoxide **2**, anionic acid **1** for control experiments.[a]

Entry	S (mM)[b]	C (mol%)[c]	B (mol%)[d]	D_2O[e]	RQ (mM)[f]	V (V)[g]	I (A)[h]	Y_m (%)[i]
1	153	0.25	-	+	-	0	0.000	0
2	153	0.25	-	+	-	12	0.000	43
3	153	0.25	0.25	+	-	0	0.000	0
4	153	0.25	0.25	+	-	12	0.000	89
5	153	0.25	-	-	153	0	0.000	0
6	153	0.25	-	-	153	12	0.000	54

[a]In microfluidic capacitor in $CDCl_3$, rt, flowrate 30 µL/min. [b]Concentration of substrate **2**, in millimolar. [c]Concentration of catalyst **1**, in mol% of substrate. [d]Concentration of ion-pair breaker thiourea **6**, in mol% of substrate. [e]Saturation with D_2O. [f]Concentration of radical quencher **4**. [g]Applied voltage, in volts. [h]Current measured, in Amperes. [i]Microfluidic yield of total cyclization products, in percent.

3.6. Voltage Dependence of the Regioselectivity of Diepoxide Cyclizations

Products of the intramolecular cyclization of *syn* and *anti* diastereoisomers of each *cis* and *trans* diepoxide **2** have been reported previously in the literature.[S8]

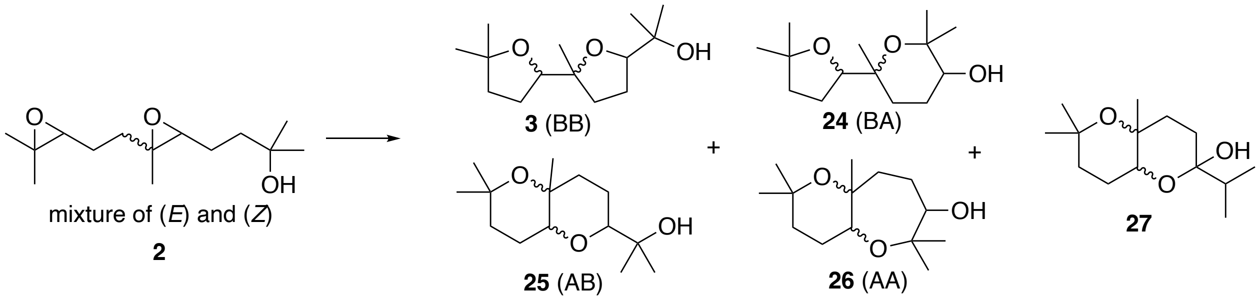

Scheme S12. All the possible products of the intramolecular cyclization of diepoxide **2**.

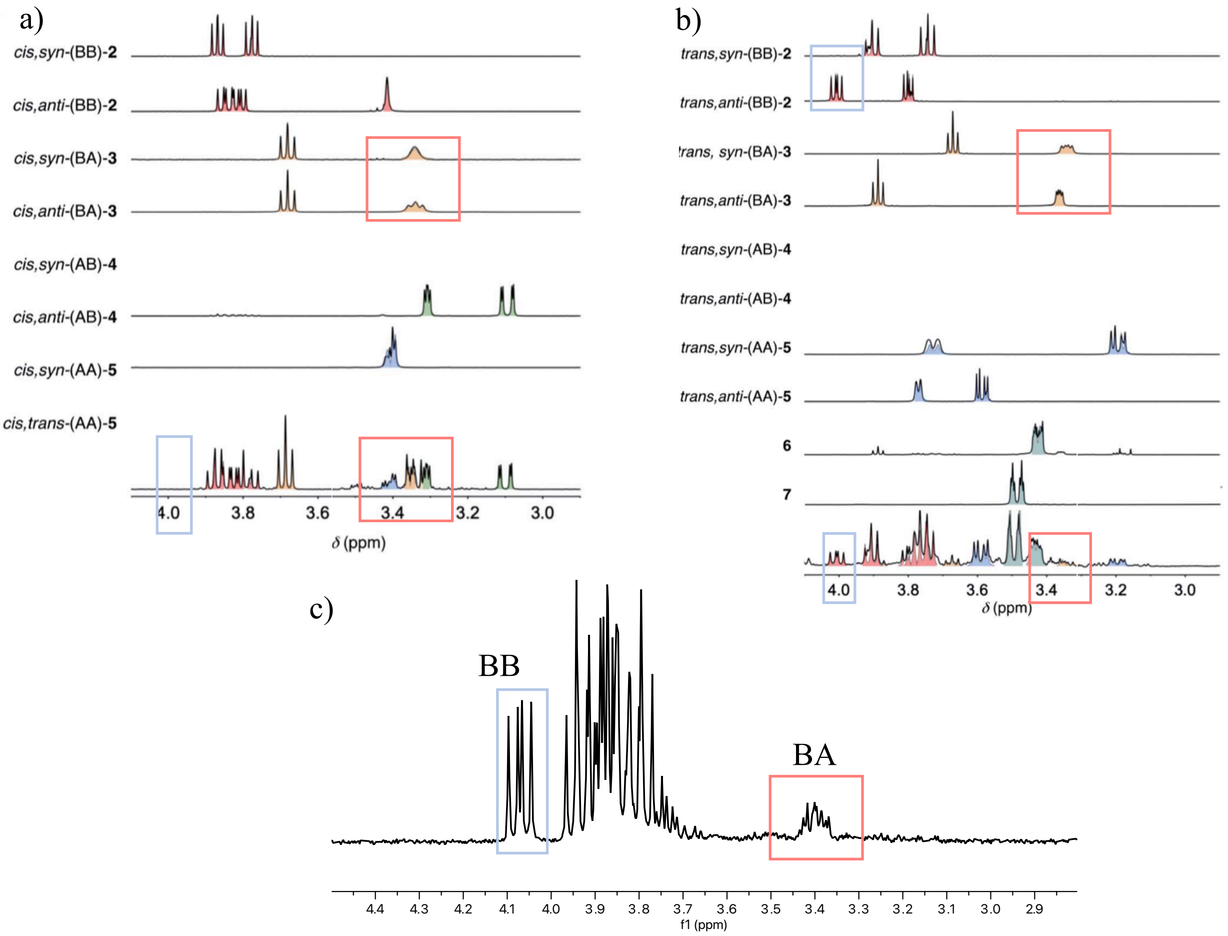

Figure S45. ^{1}H NMR signatures of characterized cyclization products of a) *Z*- and b) *E*-epoxide dimers compared with c) those from a mixture of *E* and *Z* diepoxide **2**. Figures a) and b) are from ref. S2.

Regioselectivity analysis of diepoxide cyclization with anionic acid 1 at 20 V. The sample was prepared following the general procedure for diepoxide cyclization (3.2) using anionic acid **1** (0.25 mol%) at 20 V. Regioselectivity was assessed by comparing the integral of BA cyclization products' signal at 3.4 ppm with the integral of the isolated signal of BB product at 4.1 ppm.

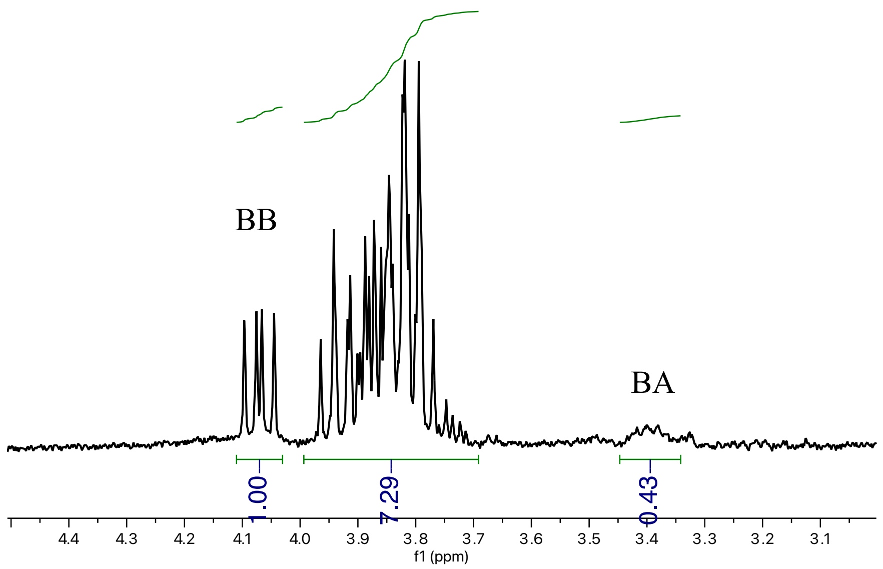

Figure S46. ^{1}H NMR regioselectivity signature region of the product mixture from cyclization of diepoxides **2** with anionic acid **1** at 20 V.

Regioselectivity analysis of diepoxide cyclization with anionic acid 1 at 0 V. A $CDCl_3$ solution of substrate **2** (153 mM), mesitylene as internal standard (153 mM), and anionic acid **1** (10 mol%) was stirred at rt until it reached complete conversion after 1 h. Reaction was followed by ^1H NMR spectroscopy. Regioselectivity was assessed by comparing the integral of BA cyclization products' signal at 3.4 ppm with the integral of the isolated signal of BB product at 4.1 ppm.

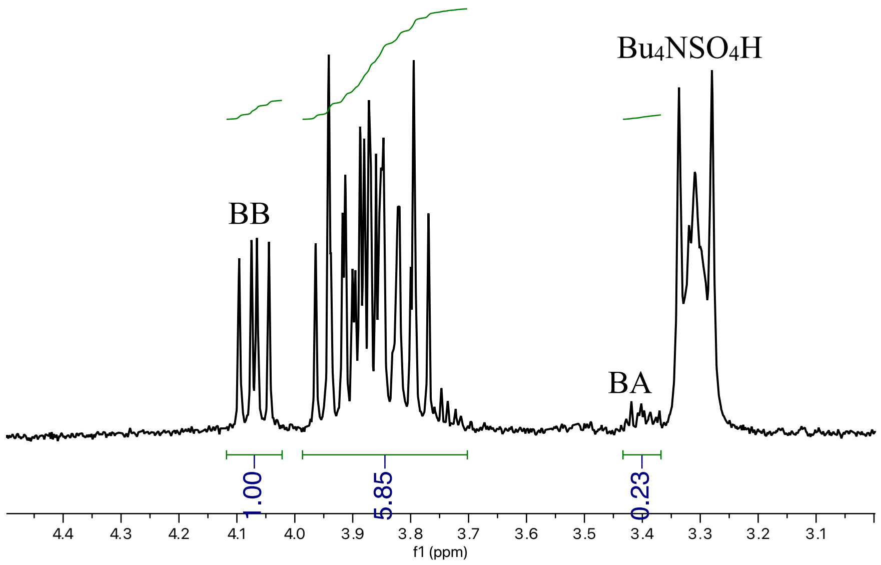

Figure S47. ^1H NMR regioselectivity signature region of the product mixture from cyclization of diepoxide **2** with anionic acid **1** at 0 V.

Regioselectivity analysis of diepoxide cyclization with anionic acid 1 and thiourea 6 at 20 V. The sample was prepared following the general procedure for diepoxide cyclization (3.2) using anionic acid **1** (0.25 mol%) and thiourea **6** (0.25 mol%) at 20 V. Regioselectivity was assessed by comparing the integral of BA cyclization products' signal (3.4 ppm) to the integral of the isolated signal of BB product (4.1 ppm).

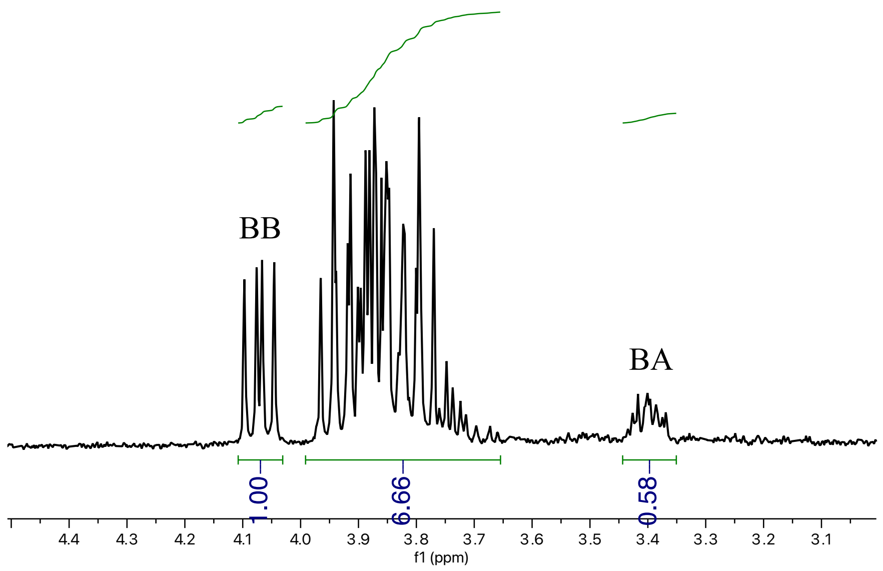

Figure S48. ^{1}H NMR regioselectivity signature region of the product mixture from cyclization of diepoxide **2** with anionic acid **1** and thiourea **6** at 20 V.

Regioselectivity analysis of diepoxide cyclization with anionic acid 1 and thiourea 6 at 0 V. A $CDCl_3$ solution containing substrate **2** (153 mM), mesitylene as internal standard (153 mM), anionic acid **1** (10 mol%), and thiourea **6** (10 mol%) was stirred at rt until it reached complete conversion after 1 h. Reaction followed by ^1H NMR. After reaching full conversion, 6 mg (12 mol%) extra of **1** was added to shift the signals of Bu_4NSO_4H and H_2O and allow integration of the cyclization products. Regioselectivity was assessed by comparing the integral of BA cyclization products' signal (3.4 ppm) to the integral of the isolated signal of BB product (4.1 ppm).

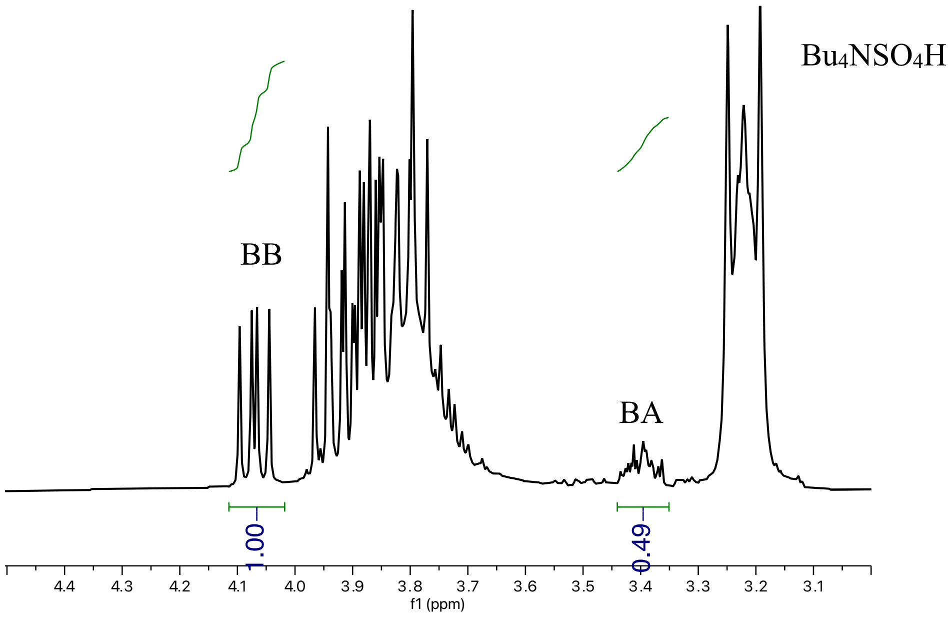

Figure S49. ^1H NMR regioselectivity signature region of the product mixture from cyclization of diepoxide **2** with anionic acid **1** and thiourea **6** at 0 V.

Regioselectivity analysis of diepoxide cyclization with anionic acid 1 and thiourea 14 at 20 V. The sample was prepared following the general procedure for diepoxide cyclization (3.2) using anionic acid **1** (0.25 mol%) and thiourea **14** (0.25 mol%) at 20 V. Regioselectivity was assessed by comparing the integral of BA cyclization products' signal (3.4 ppm) to the integral of the isolated signal of BB product (4.1 ppm).

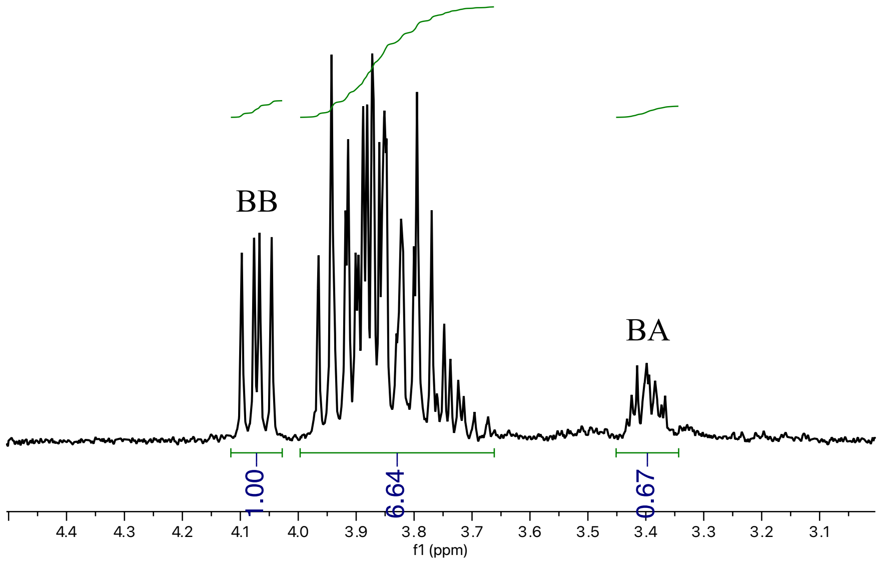

Figure S50. ^{1}H NMR regioselectivity signature region of the product mixture from cyclization of diepoxide **2** with anionic acid **1** and thiourea **14** at 20 V.

Regioselectivity analysis of diepoxide cyclization with anionic acid 1 and thiourea 14 at 0 V. A $CDCl_3$ solution containing substrate **2** (153 mM), mesitylene as internal standard (153 mM), anionic acid **1** (10 mol%) and thiourea **14** (10 mol%) was stirred at rt until it reached complete conversion after 1 h. Reaction followed by ^1H NMR spectroscopy. After reaching full conversion, 6 mg (12 mol%) extra of **1** was added to shift the signals of Bu_4NSO_4H and H_2O and allow integration of the cyclization products. Regioselectivity was assessed by comparing the integral of BA cyclization products' signal (3.4 ppm) to the integral of the isolated signal of BB product (4.1 ppm).

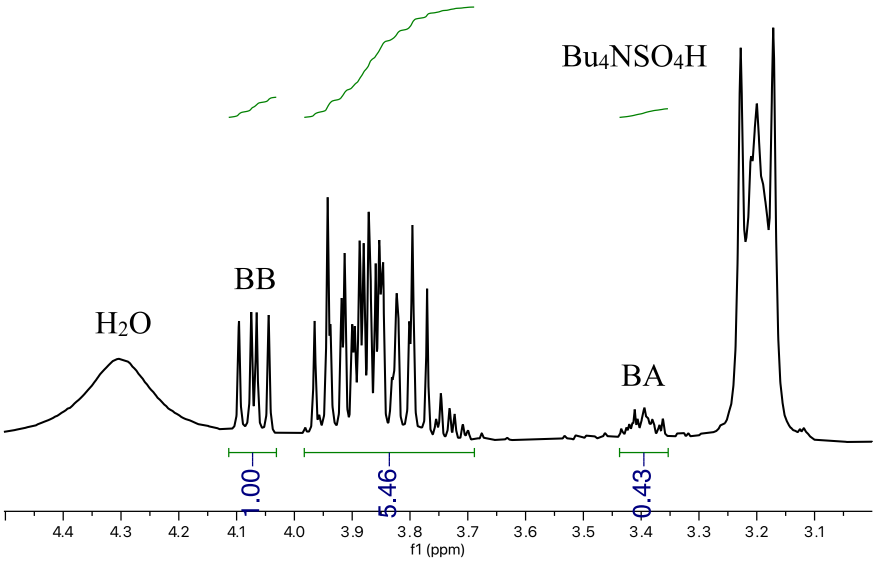

Figure S51. ^1H NMR regioselectivity signature region of the product mixture from cyclization of diepoxide **2** with anionic acid **1** and thiourea **14** at 0 V.

Regioselectivity analysis of diepoxide cyclization with anionic acid 1 and thiourea 11 at 20 V. The sample was prepared following the general procedure for diepoxide cyclization (3.2) using anionic acid **1** (0.25 mol%) and thiourea **11** (0.25 mol%) at 20 V. Regioselectivity was assessed by comparing the integral of BA cyclization products' signal (3.4 ppm) to the integral of the isolated signal of BB product (4.1 ppm).

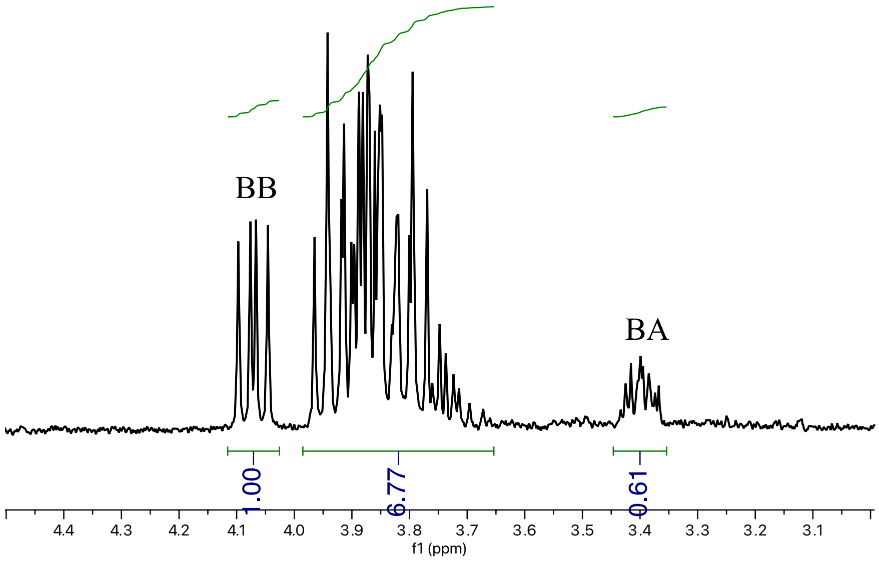

Figure S52. ^{1}H NMR regioselectivity signature region of the product mixture from cyclization of diepoxide **2** with anionic acid **1** and thiourea **11** at 20 V.

Regioselectivity analysis of diepoxide cyclization with anionic acid 1 and thiourea 12 at 20 V. The sample was prepared following the general procedure for diepoxide cyclization (3.2) using anionic acid **1** (0.25 mol%) and thiourea **12** (0.25 mol%) at 20 V. Regioselectivity was assessed by comparing the integral of BA cyclization products' signal (3.4 ppm) to the integral of the isolated signal of BB product (4.1 ppm).

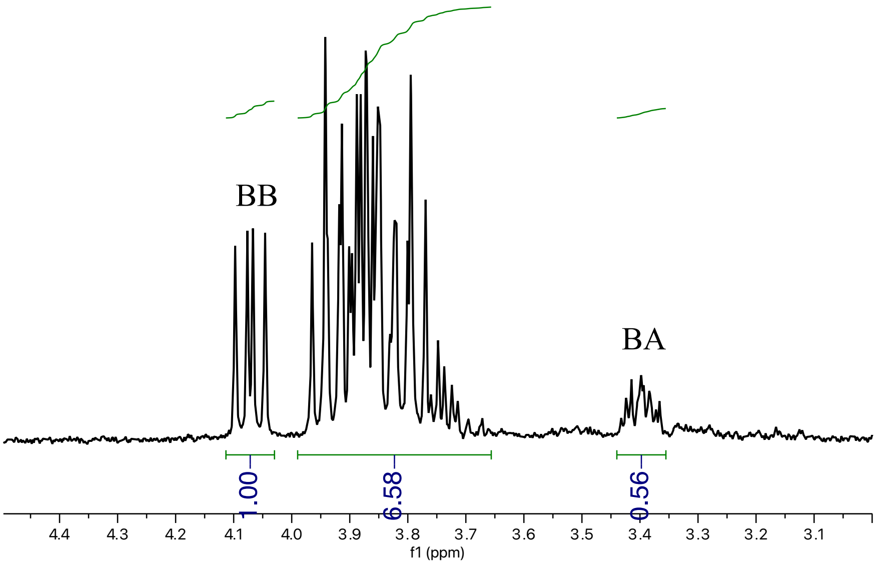

Figure S53. ^{1}H NMR regioselectivity signature region of the product mixture from cyclization of diepoxide **2** with anionic acid **1** and thiourea **12** at 20 V.

Regioselectivity analysis of diepoxide cyclization with anionic acid 1 and thiourea 13 at 20 V. The sample was prepared following the general procedure for diepoxide cyclization (3.2) using anionic acid **1** (0.25 mol%) and thiourea **13** (0.25 mol%) at 20 V. Regioselectivity was assessed by comparing the integral of BA cyclization products' signal (3.4 ppm) to the integral of the isolated signal of BB product (4.1 ppm).

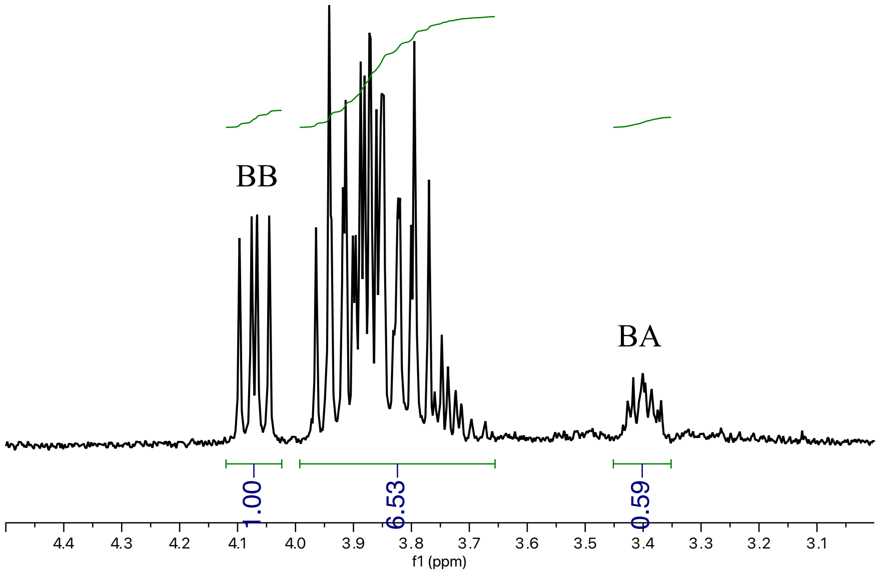

Figure S54. ^{1}H NMR regioselectivity signature region of the product mixture from cyclization of diepoxide **2** with anionic acid **1** and thiourea **13** at 20 V.

Table S19. EFC with substrate **2**, anionic acid **1** and with various thioureas.

Entry	Thiourea	S (mM)[c]	C (mol%)[d]	B (mol%)[e]	V (V)[f]	I (A)[g]	Y_m (%)[h]	BA
1[a]	-	153	0.25	-	20	0.000	54	0.43
2[b]	-	153	10	-	0	–	100	0.23
3[a]	**6**	153	0.25	0.25	20	0.000	94	0.58
4[b]	**6**	153	10	10	0	–	100	0.49
5[a]	**14**	153	0.25	0.25	20	0.000	97	0.67
6[b]	**14**	153	10	10	0	–	100	0.43
7[a]	**11**	153	0.25	0.25	20	0.000	99	0.61
8[a]	**12**	153	0.25	0.25	20	0.000	98	0.56
9[a]	**13**	153	0.25	0.25	20	0.000	97	0.59

[a]In microfluidic capacitor in $CDCl_3$, rt, flowrate 30 µL/min. [b]In a vial in $CDCl_3$ at rt. [c]Concentration of substrate **2**, in millimolar. [d]Concentration of catalyst **1**, in mol% of substrate. [e]Concentration of ion-pair breaker, in mol% of substrate. [f]Applied voltage, in volts. [g]Current measured, in amperes. [h]Microfluidic yield of total cyclization products, in percent.

3.7. Voltage Dependence of Triepoxide and Tetraepoxide Cyclizations

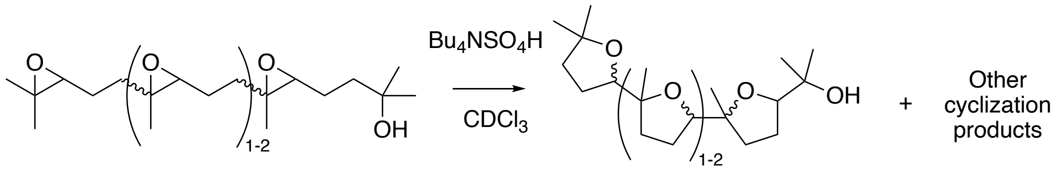

Scheme S13. Cyclization reaction of **20** and **22** catalyzed by EF with anionic acid **1**.

Without Ion-Pair Breaker. A $CDCl_3$ solution containing substrate **20** or **22** (29 mM), mesitylene as internal standard (29 mM) and anionic acid **1** (5 mol% for **20** and 10 mol% for **22**) was loaded in a syringe. The solution was infused at 30 μL/min using a syring pump into the microfluidic capacitor under constant voltage. One and a half reactor volumes (0.45 mL) were discarded before collecting each sample to ensure that a steady state of the system had been reached at the desired voltage. Samples of 150 μL were collected during a period of 5 min. Samples were diluted with additional $CDCl_3$, and ^1H NMR spectra were acquired immediately after each experiment. Yield was determined by comparing the integral of the epoxide signal to that of the internal standard. All experiments were conducted under I = 0.000 A. Triepoxides **20** and tetraepoxide **22** were prepared with 13% and 15% of cyclization product, respectively, which were not considered in the reported EFC reaction yields.

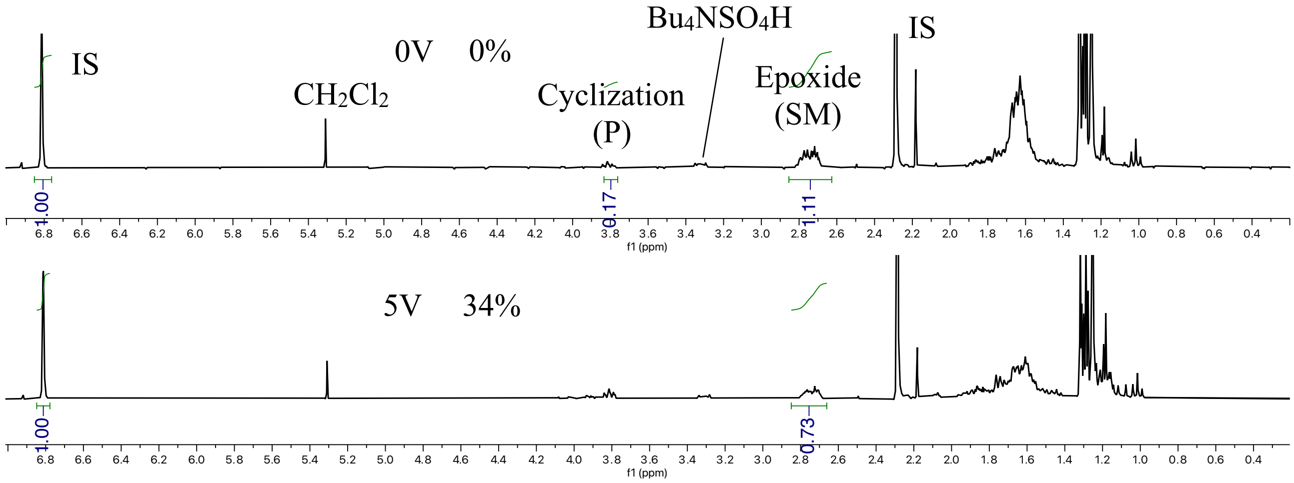

Figure S55. ^{1}H NMR spectra of the cyclization reaction mixture of **20** under EFC with anionic acid **1** in $CDCl_3$ at 0 and 5 V.

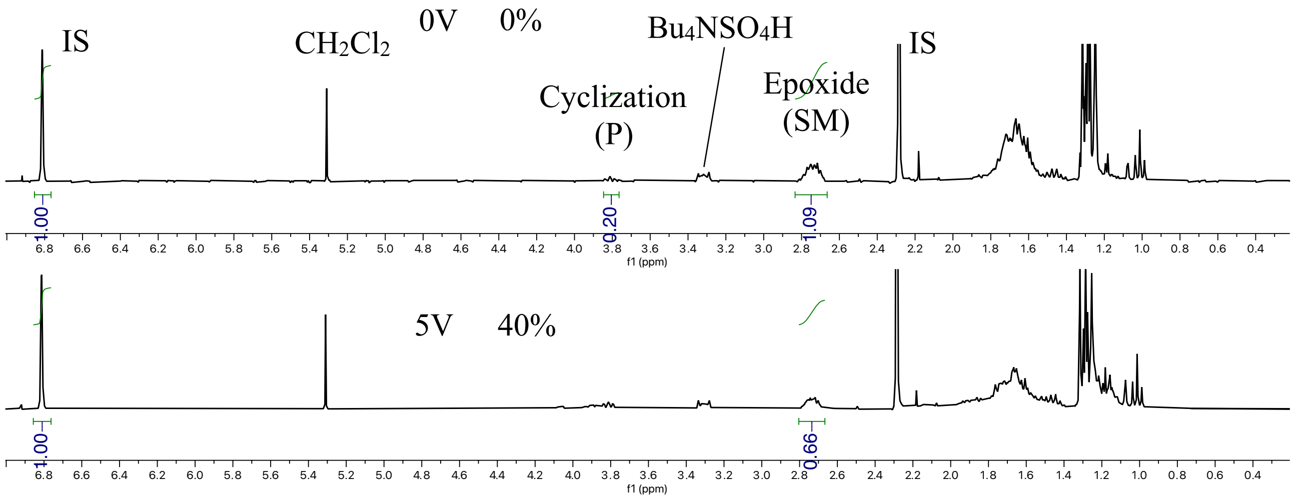

Figure S56. ^{1}H NMR spectra of the cyclization reaction mixture of **22** under EFC with anionic acid **1** in $CDCl_3$ at 0 and 5 V.

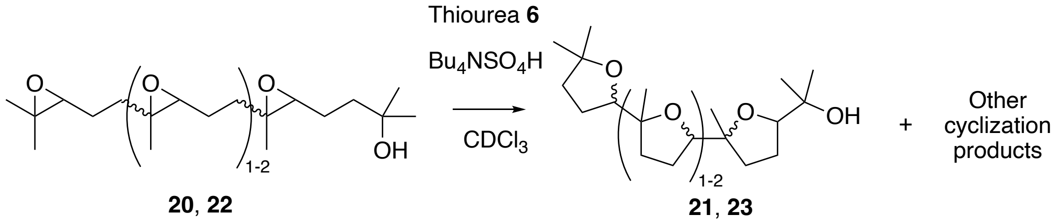

Scheme S14. Cyclization reaction of **20** and **22** catalyzed by EF with anionic acid **1** and thiourea **6**.

With Ion-Pair Breaker. A $CDCl_3$ solution containing substrate **20** or **22** (29 mM), mesitylene as internal standard (29 mM), anionic acid **1** (5 mol% for **20** and 10 mol% for **22**) and thiourea **6** (5 mol% for **20** and 10 mol% for **22**) was loaded in a single syringe. The solution was infused at 30 μL/min into the microfluidic capacitor under constant voltage. One and a half reactor volumes (0.45 mL) were discarded before collecting each sample to ensure that a steady state of the system had been reached at the desired voltage. Samples of 150 μL were collected during a period of 5 min. Samples were diluted with additional $CDCl_3$, and ^1H NMR spectra were acquired immediately after each experiment. Yield was determined by comparing the integral of the epoxide signal to that of the internal standard. All experiments were conducted under $I = 0.000$ A. Triepoxide **20** and tetraepoxide **22** were prepared with 13% and 15% of cyclization product, respectively, which were not considered in the reported EFC reaction yields.

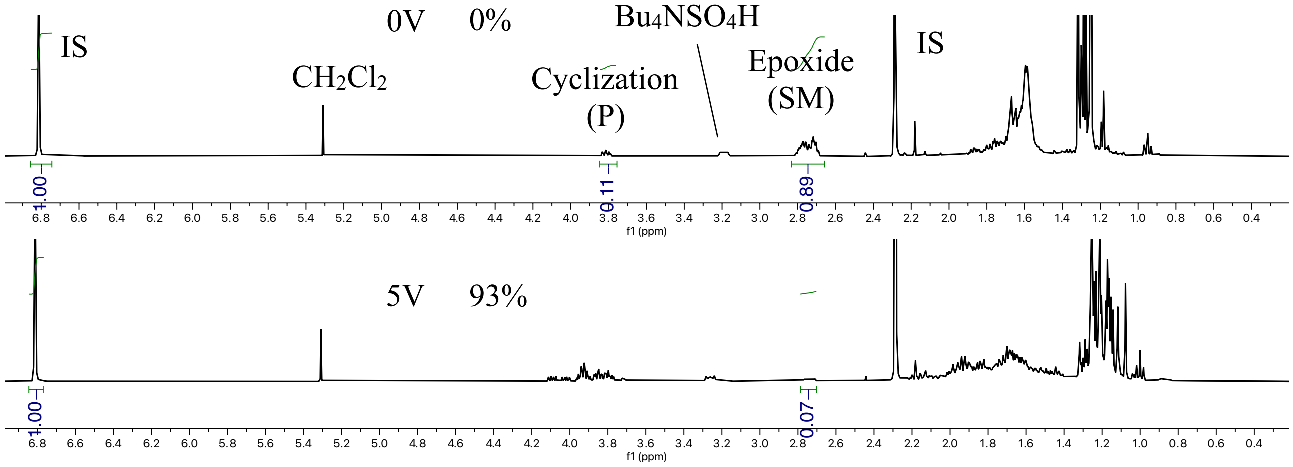

Figure S57. ^{1}H NMR spectra of the cyclization reaction of **20** under EFC with anionic acid **1** and thiourea **6** in $CDCl_3$ at 0 and 5 V.

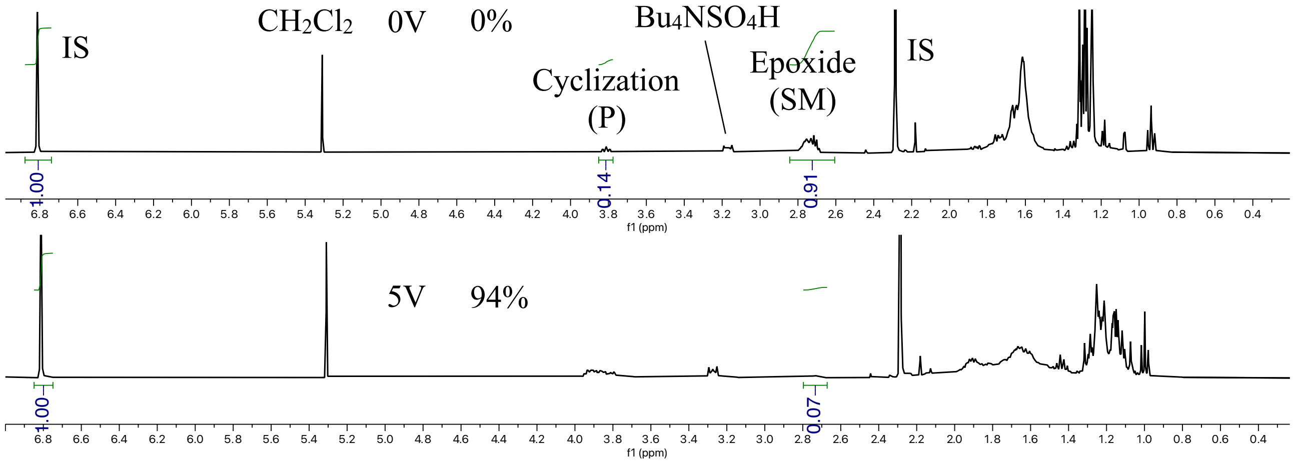

Figure S58. ^{1}H NMR spectra of the cyclization reaction of **22** under EFC with anionic acid **1** and thiourea **6** in $CDCl_3$ at 0 and 5 V.

Table S20. EFC with substrate **20** and **22**, anionic acid **1** and with and without thiourea **6**.[a]

Entry	Epoxide	S (mM)[b]	C (mol%)[c]	B (mol%)[d]	*V* (V)[e]	*I* (A)[f]	Y_m (%)[g]
1	**20**	29	5	-	0	0.000	0
2	**20**	29	5	-	5	0.000	34
3	**20**	29	5	5	0	0.000	0
4	**20**	29	5	5	5	0.000	93
5	**22**	29	10	-	0	0.000	0
6	**22**	29	10	-	10	0.000	40
7	**22**	29	10	10	0	0.000	0
8	**22**	29	10	10	10	0.000	94

[a]In microfluidic capacitor in $CDCl_3$, rt, flowrate 30 µL/min. [b]Concentration of substrate **20** and **22**, in millimolar. [c]Concentration of catalyst **1**, in mol% of substrate. [d]Concentration of ion-pair breaker thiourea **6**, in mol% of substrate. [e]Applied voltage, in volts. [f]Current measured, in amperes. [g]Microfluidic yield of total cyclization products, in percent.

4. Supporting References

(S1) Gutiérrez López, M. Á.; Ali, R.; Tan, M.-L.; Sakai, N.; Wirth, T.; Matile, S. *Sci. Adv.* **2023**, *9*, eadj5502.

(S2) Paraja, M.; Hao, X.; Matile, S. *Angew. Chem. Int. Ed.* **2020**, *59*, 15093–15097.

(S3) Basumatary, G. G.; Bez, G. *Synthesis* **2023**, *55*, 786–798.

(S4) Sibi, M. P.; Itoh, K. *J. Am. Chem. Soc.* **2007**, *129*, 8064–8065.

(S5) Akhtar, N.; Saha, A.; Kumar, V.; Pradhan, N.; Panda, S.; Morla, S.; Kumar, S.; Manna, D. *ACS Appl. Mater. Interfaces* **2018**, *10*, 33803–33813.

(S6) Yamada, T.; Kuwata, M.; Takakura, R.; Monguchi, Y.; Sajiki, H.; Sawama, Y. *Adv. Synth. Catal.* **2018**, *360*, 637–641.

(S7) Rampalakos, C.; Wulff, W. D. *Adv. Synth. Catal.* **2008**, *350*, 1785–1790.

(S8) Chen, H.; Li, T.-R.; Sakai, N.; Besnard, C.; Guenee, L.; Pupier, M.; Viger-Gravel, J.; Tiefenbacher, K.; Matile, S. *Chem. Sci.* **2022**, *13*, 10273–10280.

5. NMR Spectra

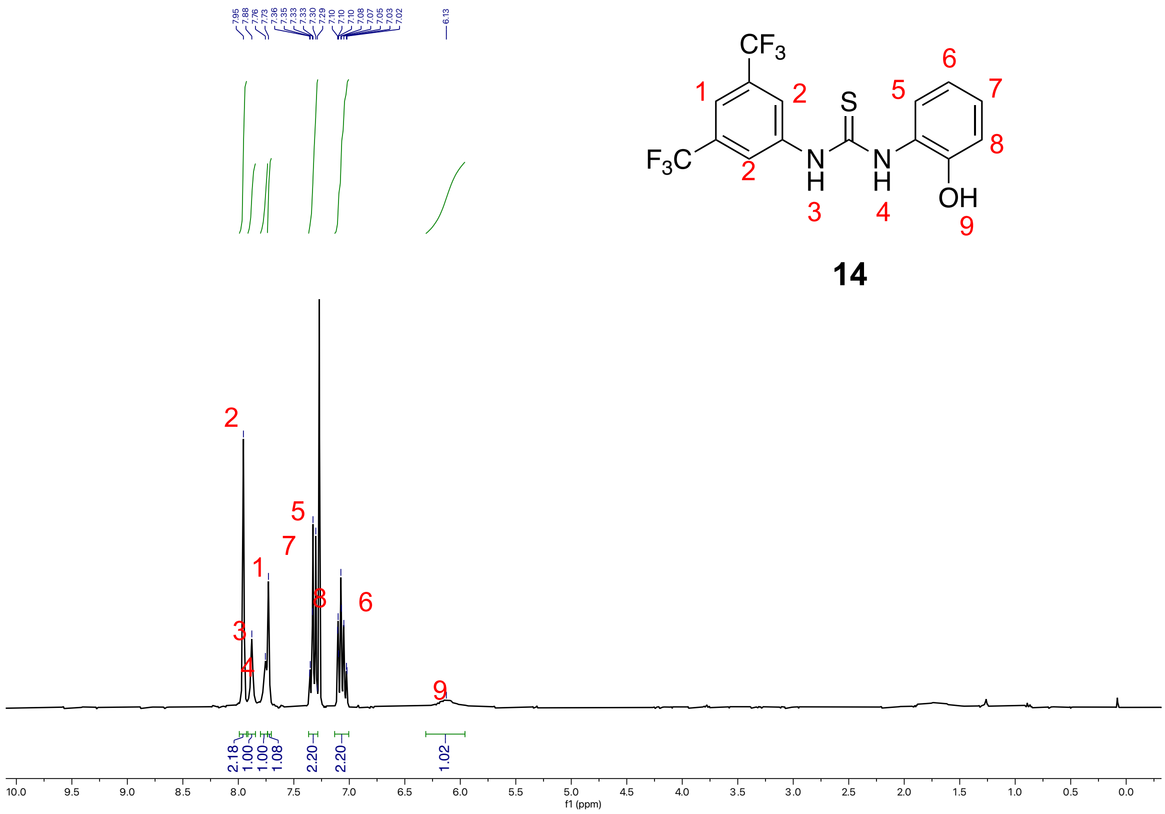

Figure S59. 300 MHz ^{1}H NMR spectra of thiourea **14** in $CDCl_3$.

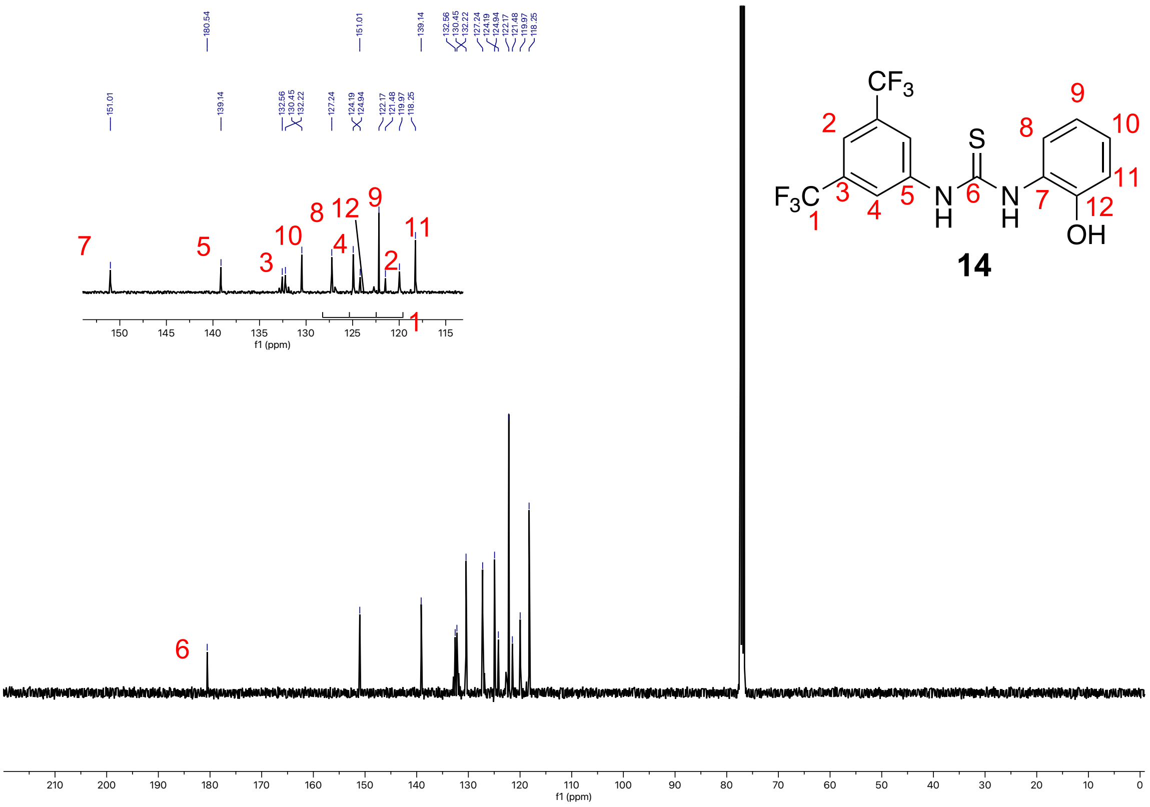

Figure S60. 101 MHz ^{13}C NMR spectra of thiourea **14** in $CDCl_3$.

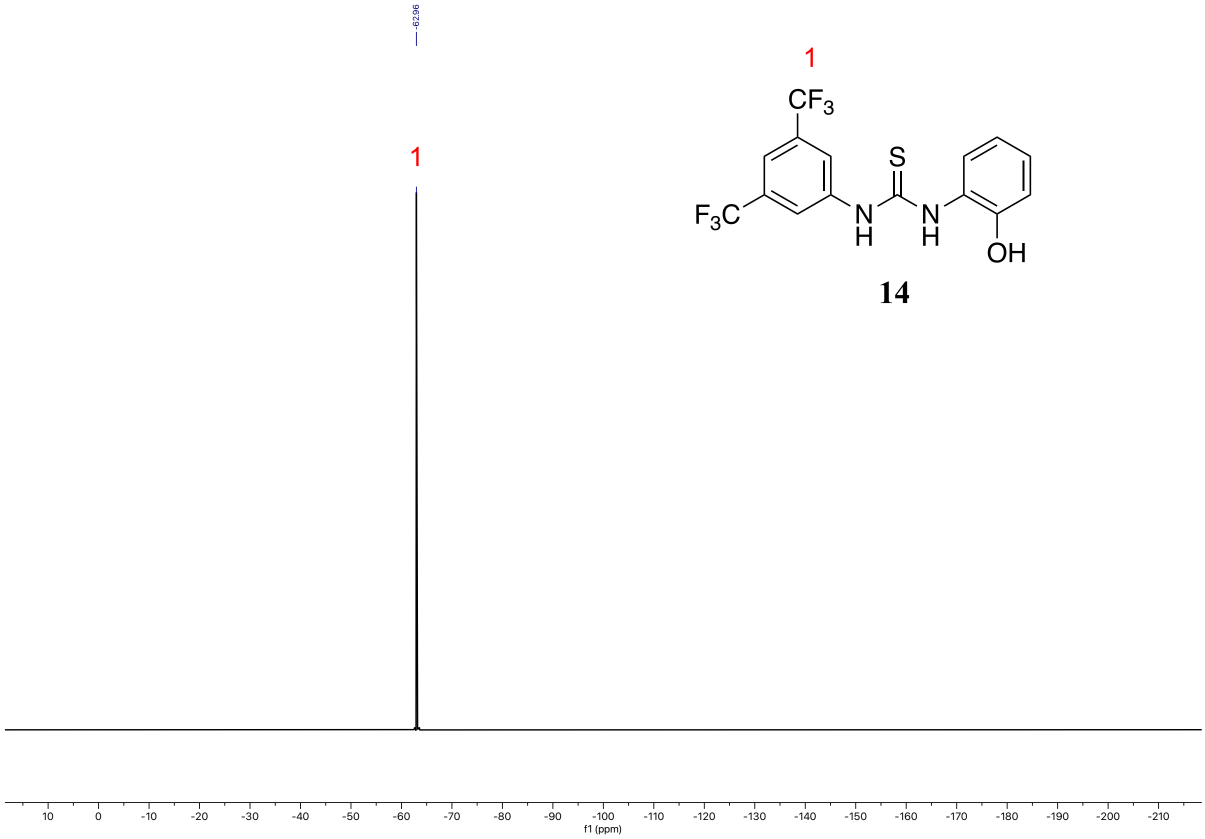

Figure S61. 282 MHz ^{19}F NMR spectra of thiourea **14** in $CDCl_3$.

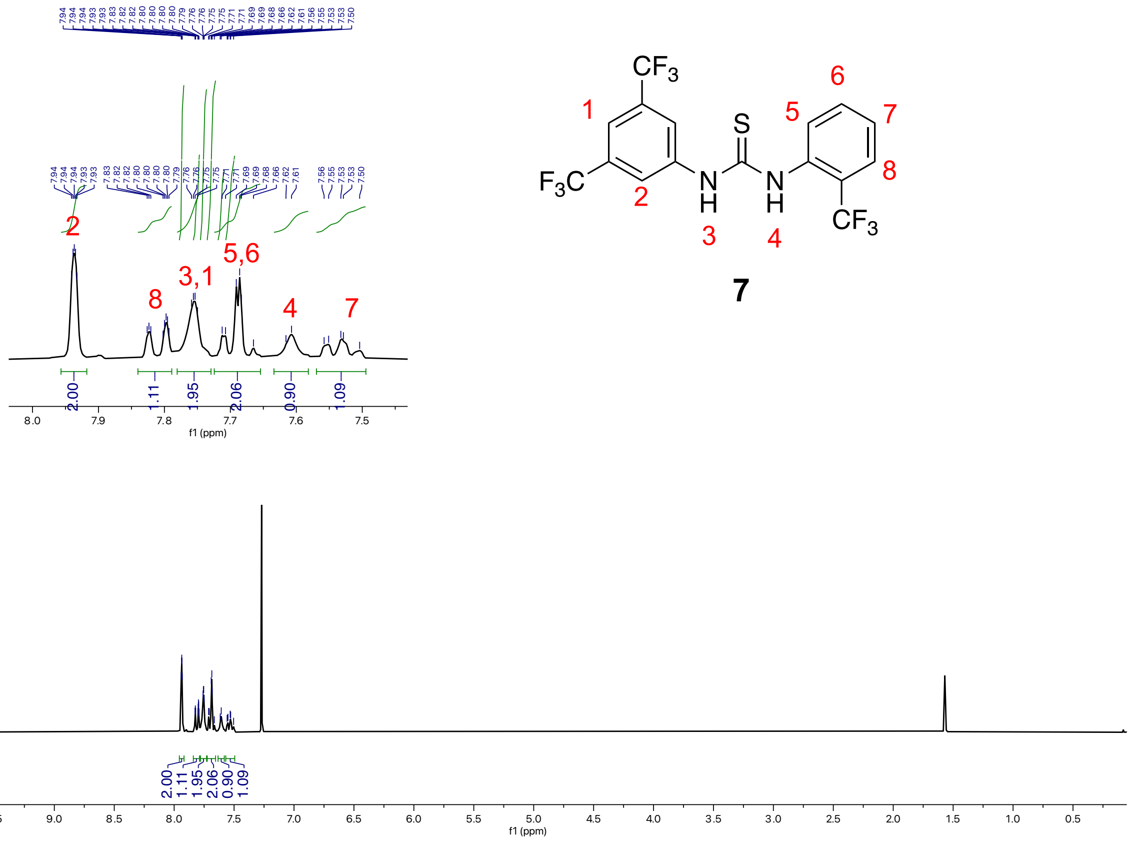

Figure S62. 300 MHz ^{1}H NMR spectra of thiourea **7** in $CDCl_3$.

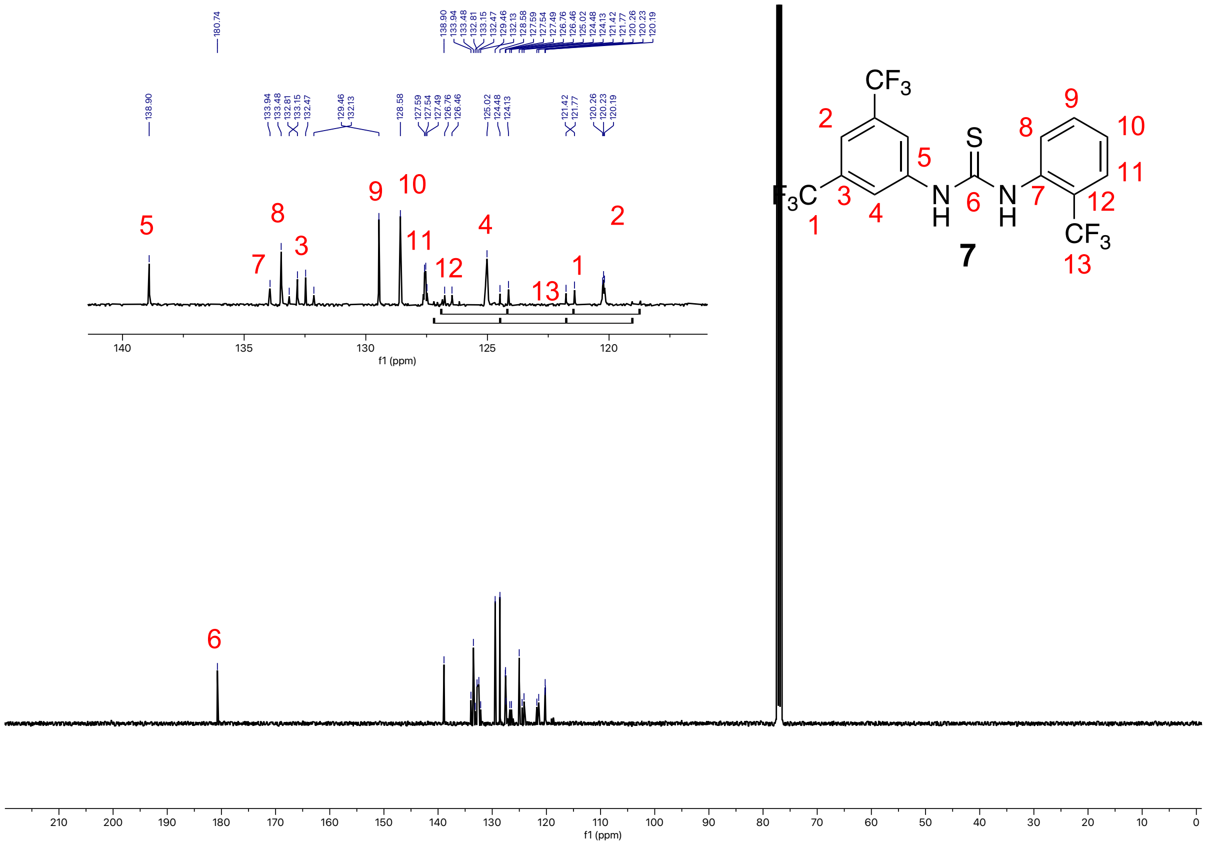

Figure S63. 101 MHz ^{13}C NMR spectra of thiourea **7** in $CDCl_3$.

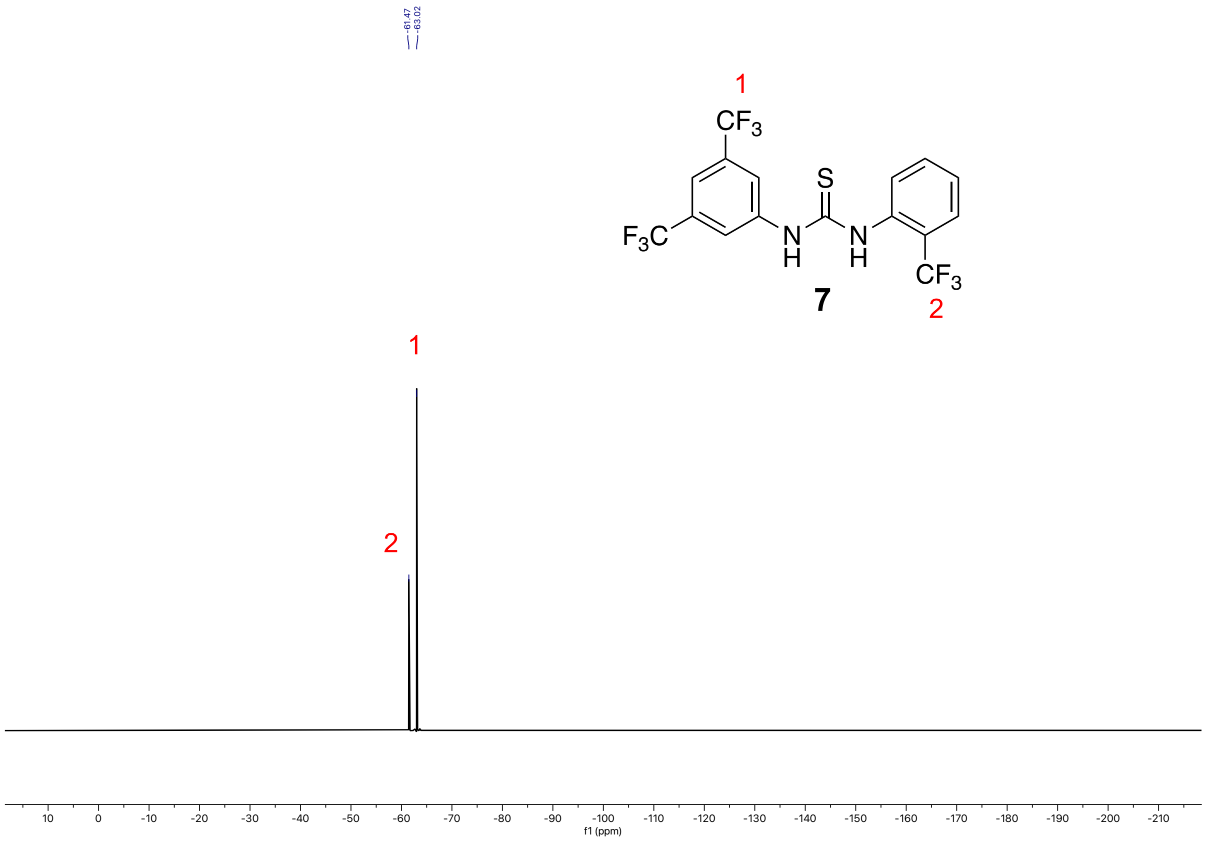

Figure S64. 282 MHz ^{19}F NMR spectra of thiourea **7** in $CDCl_3$.
